# Scaffold Fusion and SAR Transfer with a Chemical Language Model Generates Novel Liver X Receptor Modulators

**DOI:** 10.1021/acs.jmedchem.5c01551

**Published:** 2025-10-23

**Authors:** Nils Christiaan Bandomir, Tim Hörmann, Annette Kärcher, Astrid Kaiser, Daniel Merk, Pascal Heitel

**Affiliations:** † Institute of Pharmaceutical Chemistry, 9173 Goethe University Frankfurt, Max-von-Laue-Strasse 9, 60438 Frankfurt, Germany; ‡ Department of Pharmacy, 9183 Ludwig-Maximilians-Universität (LMU) München, Butenandtstrasse 5-13, 81377 Munich, Germany

## Abstract

Liver X receptors (LXRs) are promising targets for metabolic disorders including atherosclerosis and metabolic dysfunction-associated steatotic liver disease (MASLD). In this study, we employed a chemical language model (CLM) for LXR modulator design in an explorative fashion and observed that structural features from different LXR modulator templates were merged, and structure–activity relationship (SAR) knowledge was transferred. The generated computational designs demonstrated LXR modulation with diverse activity profiles and selective modulator properties, including a promising lipolytic activity of an inverse LXR agonist in an in vitro MASLD model that warrants its further development to improve ADME properties.

## Graphical abstract

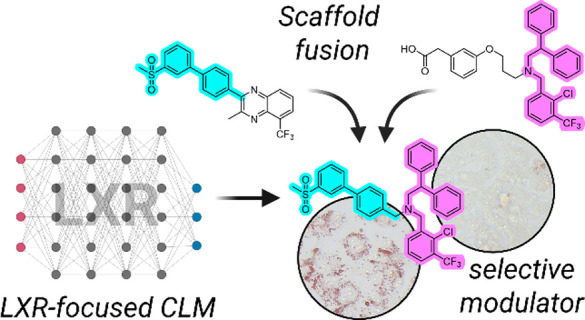

## Introduction

Liver X receptors (LXRs) are ligand-controlled transcription factors that play a critical role in cholesterol homeostasis, lipid metabolism, and inflammation.^1–4^ Both highly conserved LXR subtypes (LXRα, NR1H3; LXRβ, NR1H2)^5,6^ are activated by oxidized cholesterol metabolites, so-called oxysterols,^7,8^ to induce transcription of genes enhancing reverse cholesterol transport to the liver. Given this pivotal role in cholesterol balance, LXRs are considered as potential targets for the treatment of diseases associated with dysregulated cholesterol homeostasis such as atherosclerosis and Alzheimer’s disease. Several synthetic LXR ligands have been developed and studied in early clinical trials.^4,9^ Despite attractive effects, enhanced hepatic de novo lipogenesis due to LXRα-mediated induction of sterol regulatory element-binding protein 1c (SREBP1c) has hindered the progression of LXR agonists. Oppositely, inhibition of LXR in hepatic lipogenesis with inverse agonists has been identified as a promising approach for the treatment of metabolic dysfunction-associated steatotic liver disease (MASLD).^10^ To expand the collection of LXR modulator profiles and further explore the potential of LXR ligands in different indications, we here aimed to identify new LXR modulators using a chemical language model (CLM) as an innovative tool for medicinal chemistry.

CLMs^11,12^ are deep learning models trained with molecules in string format such as SMILES^13^ and are capable of navigating chemical space.^12^ A CLM is typically developed by pretraining a recurrent long short-term memory (LSTM)^14^ network with large corpora of SMILES and subsequent fine-tuning with a small collection of task-specific template molecules.^11,15–17^ This two-step procedure allows the development of a general CLM that captures the syntax of SMILES and its subsequent transfer to a specific task. Previous applications have demonstrated the ability of CLMs to design innovative new chemical entities^18–20^ with activity on intended targets in a data-driven fashion.^18,19,21,22^

Herein, we describe the identification of novel LXR modulators by the explorative application of CLM-driven de novo design. The obtained designs fused structural features of different LXR ligand scaffolds and exhibited innovative LXR modulator activity.

## Results and Discussion

### LXR Ligand Design by CLM

To develop novel LXR ligands, we used an existing CLM^19^ pretrained with 365,063 molecules from ChEMBL and performed fine-tuning with LXR modulators using 10-fold data augmentation (Figure 1a). In initial experiments, we used a small but chemically diverse collection of 12 manually selected LXR modulator templates for fine-tuning. However, the beam search designs^19^ of the resulting CLMs were enriched in pretraining artifacts such as phosphates and pyrophosphates (Chart S1), suggesting that the model could not sufficiently capture the chemical space of interest from the small collection. Therefore, we applied an augmented two-stage fine-tuning procedure using initially a larger set of 252 LXR ligands (EC_50_/IC_50_/*K*_d_ ≤ 1 μM) from BindingDB, followed by the 12 selected molecules. Eight fine-tuning epochs (5–12) for LXR ligand candidate design were selected for sampling based on the Tanimoto similarity (Morgan fingerprints, radius 2, 2048-bit) of the beam search (width 50) designs to the fine-tuning set. From each selected epoch, we sampled 2000 SMILES with temperature sampling (*T* = 0.2). Designs were then prioritized (Table S1) using the model’s sampling frequency as an intrinsic quality measure. Additionally, the 1000 most similar designs to the template set based on Morgan fingerprints (Table S2) were evaluated for their ability to interact with the intended target by docking to ligand-bound structures of both LXR isoforms (PDB ID: 3IPQ,^23^ 5JY3 ^24^) using smina.^25^ **1**-**3** were selected based on frequency and docking score as promising and synthetically accessible LXR modulator designs for prospective experimental evaluation.

**Figure 1:**
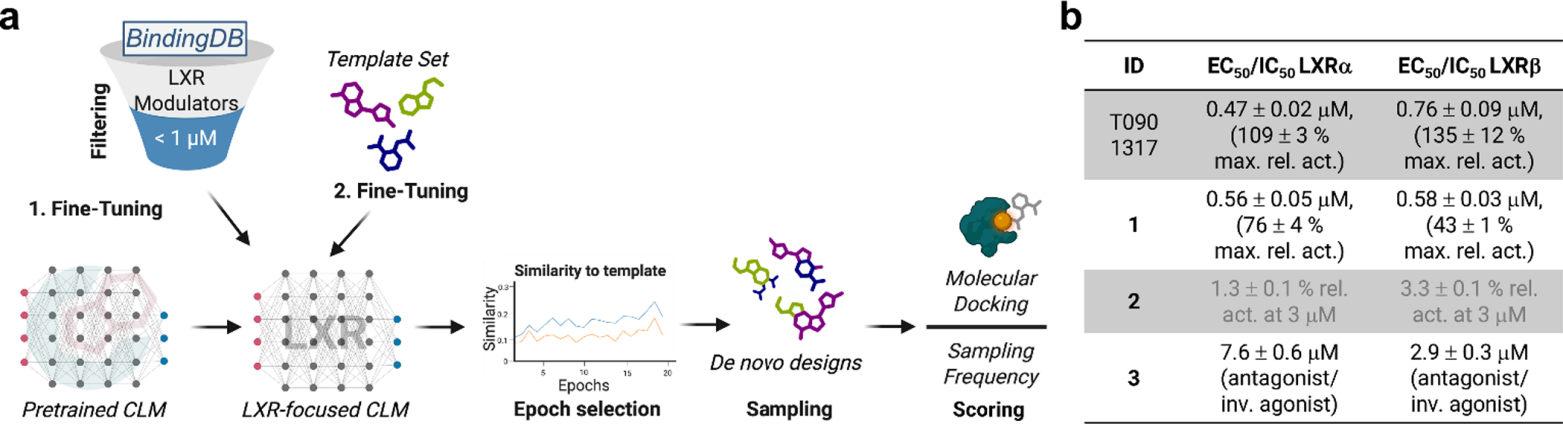
(a) Schematic overview of the explorative LXR modulator design with CLM. A CLM pretrained with 365,063 molecules from ChEMBL was fine-tuned in two stages, first with 252 LXR modulators from BindingDB (Table S3) and second with 12 manually selected LXR modulators (“Template Set”, Table S4). After epoch selection by similarity to the template molecules, de novo designs were sampled and ranked for experimental evaluation by sampling frequency and docking. (b) Evaluation of molecular designs **1**-**3** and the reference LXR agonist T0901317 in Gal4-LXR hybrid reporter gene assays. Relative activation is normalized to T0901317 at 1 μM; *N* ≥ 3. Unspecific binding events, inhibition of the reporter construct, and RXR-mediated effects of **3** were ruled out in control experiments using the ligand-independent constitutive transcriptional activator Gal4-VP16 ^54^ or a Gal4-RXRα hybrid reporter gene assay (Figure S2); inv., inverse; max. rel. act., maximum relative activation.

Compounds **1**-**3** were prepared according to Scheme 1. Electrophilic aromatic substitution of aniline **4** with hexafluoroacetone hydrate (**5**) under acidic conditions gave **6**.^26^ The intermediate was then reacted with benzoyl chloride (**7**) to form amide **1**.^27^ Compound **2** was synthesized in three steps: Chan-Lam coupling of boronic acid **8** and *N*-Boc-piperazine (**9**) provided **10**. Subsequent removal of the Boc group to **11** followed by Chan-Lam coupling of **11** and (4-chloro-3-[trifluoromethyl]­phenyl)­boronic acid (**12**) gave **2**. Compound **3** was obtained in a four-step procedure starting with the Suzuki coupling of (3-[methylsulfonyl]­phenyl)­boronic acid (**8**) with 4-bromobenzyl alcohol (**13**). The resulting alcohol **14** was converted into the corresponding bromide (**15**) with PBr_3_, and nucleophilic substitution of **15** with 2,2-diphenylethanamine (**16**) provided amine **17**. Compound **3** was then available from the reductive amination of **17** and benzaldehyde **18**.

**Scheme 1:**
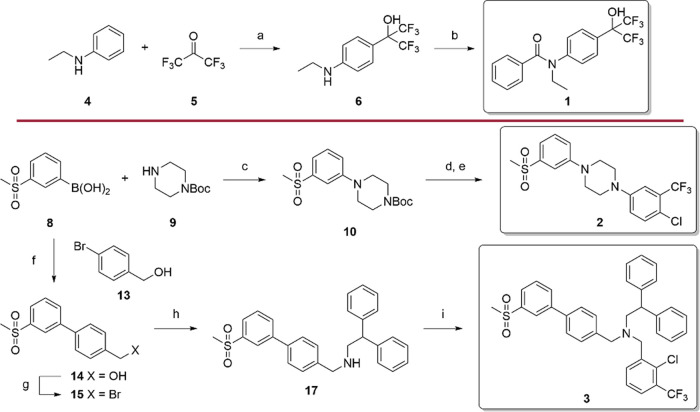
Synthesis of **1**-**3** ^a^

a Reagents and conditions: (a) *p*-TsOH, toluene, 120 °C, 3 h, 64%; (b) PhCOCl (**7**), NEt_3_, CH_2_Cl_2_, 0 °C to rt, 2 h, 67%; (c) Cu­(OAc)_2_, CH_2_Cl_2_, molecular sieves (4 Å), 45 °C, 16 h, 39%; (d) TFA, CH_2_Cl_2_, rt, 2 h, 99%; (e) (4-chloro-3-[trifluoromethyl]­phenyl)­boronic acid (**12**), Cu­(OAc)_2_, CH_2_Cl_2_, molecular sieves (4 Å), 45 °C, 16 h, 21%; (f) Pd­(PPh_3_)_4_, K_2_CO_3_, 1,4-dioxane, H_2_O, 110 °C, 2 h, 97%; (g) PBr_3_, CH_2_Cl_2_, 0 °C to rt, 2 h, 87%; (h) 2,2-diphenylethanamine (**16**), K_2_CO_3_, DMF, rt, 2 h, 66%; (i) (step 1) 2-chloro-3-(trifluoromethyl)­benzaldehyde (**18**), HOAc, DCE, rt, 2 h; (step 2) NaBH­(OAc)_3_, 16 h, 32%.

Compounds **1**-**3** were evaluated for their ability to modulate LXRs in hybrid reporter gene assays based on fusion proteins of the LXRα or LXRβ ligand binding domain (LBD) and the Gal4 DNA binding domain (from yeast).^28^ This in vitro test identified **1** and **2** as partial agonists on both LXR subtypes despite the weak efficacy of **2** (Figures 1b, S1, and S2). Compared with the reference agonist T0901317, design **1** was similarly potent and balanced on the two LXR subtypes. Design **3** did not activate LXRs but suppressed LXR activation by the reference agonist T0901317 ^29^ in competitive assays (Figures 1b, S1, and S2) with single-digit micromolar IC_50_ values and a slight preference for LXRβ. This initial in vitro profiling thus confirmed LXR modulation by all computational designs, corroborating the design approach.

The computational designs **1**-**3** displayed Tanimoto similarities of 0.35–0.51 to the most similar known LXR ligands (Figure 2a, Chart S2) and were found in t-distributed stochastic neighbor embedding (t-SNE, Figure 2b) in regions of the chemical space that are also populated by known LXR modulators. While **1** resembled the LXR agonist T0901317, closer inspection of **2** and **3** revealed that both designs inherited features from two LXR ligand scaffolds in the training data: **2** combined the scaffolds and substitution patterns of VTP-766 ^30^ and GW3965,^31^ whereas **3** represented a fusion of WYE-672 ^32^ and GW3965. These results indicated that the CLM was capable of extracting relevant chemical features for LXR modulation from the training data and reassembling them in a meaningful (i.e., active) way to create new ligand scaffolds. In a simulated virtual screening of the Enamine screening collection spiked with the de novo designs **1**-**3** using Tanimoto similarity computed on Morgan fingerprints to the fine-tuning set I (Table S3) as the ranking criterion, **1**-**3** were not ranked high, supporting their novelty (Chart S3). Nevertheless, each design displayed similarity to a single LXR ligand scaffold (Chart S4). Key physicochemical properties of designs **1** and **3** such as molecular weight, topological polar surface area, and the fraction of sp^3^ carbon atoms are similar to literature LXR modulators (Figure 2c). The high lipophilicity^33^ of design **3** is an unfavorable property for drug development, but LXR is known to bind more lipophilic compounds than other nuclear receptors (Figure S3). Nevertheless, compounds **1** and **3** displayed a high metabolic stability (>60 min) in rat liver microsomes (Figure 2d), rendering them suitable for further biological evaluation.

**Figure 2:**
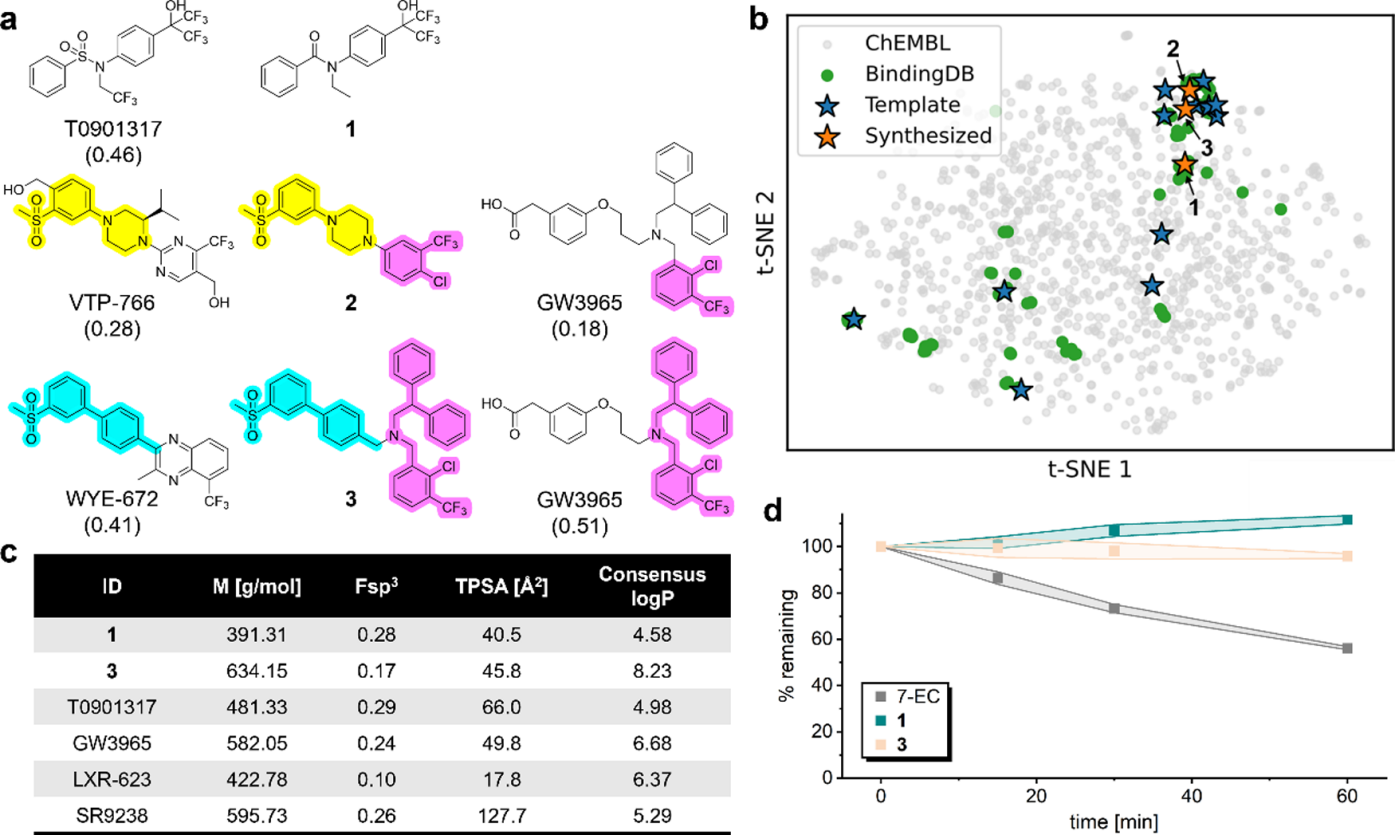
(a) Structural comparison of CLM designs to known LXR ligands used for fine-tuning. Numbers refer to the Tanimoto similarity computed on Morgan fingerprints. The colors denote the fused scaffolds. Comparison with the most similar fine-tuning molecules (stage 2) is shown in Chart S2. (b) t-distributed stochastic neighbor embedding (t-SNE) showing the distribution of LXR ligands in the training data (Binding DB – stage 1; Templates – stage 2) and computational designs **1**-**3**. 10,000 random molecules from ChEMBL as background. (c) Designs **1** and **3** resemble known LXR modulators in basic molecular properties (fraction of sp^3^ carbon, total polar surface area, and consensus log*P* as calculated from SwissADME^33^). However, design **3** is very lipophilic. (d) Designs **1** and **3** are stable for >1 h in liver microsomes from Sprague–Dawley rats. 7-Ethoxycoumarine (7-EC) served as a control. Data are the mean ± SD; *N* = 3.

### Characterization and Application of CLM-Designed LXR Modulators

Intrigued by the initial data on LXR modulation, we further investigated the mode-of-action of designs **1**-**3.** As the Gal4 hybrid reporter gene assay is artificial and overexpresses LXR, we next examined LXR modulation by **1**-**3** in a full-length LXR assay (Figure 3a)^34^ based on the endogenous expression of the LXR/retinoid X receptor (RXR) heterodimer (Figure S2). Upon activation, LXR binds to response elements within the ATP-binding cassette transporter A1 (ABCA1, an LXR target gene) promoter of the reporter. This setting displays basal LXR activation, allowing bidirectional modulation. In HEK293T and HepG2 cells, compounds **1** and **2** were confirmed as partial LXR agonists, while **3** robustly blocked LXR activation, classifying it as an antagonist or inverse agonist, depending on whether basal activity is an intrinsic property of the receptor or stems from the presence of endogenous ligands (oxysterols). The designs were next checked for antiproliferative effects that may compromise further in vitro characterization. At 10 μM (**1** and **2**) or 30 μM (**3**), the designs did not inhibit the proliferation of HEK293T or HepG2 cells (Figure 3b). HEK293T cells treated with design **2** at 10 μM displayed a slightly reduced growth rate. Therefore, and due to the weak efficacy of **2**, we focused further biological characterization on **1** and **3**.

**Figure 3:**
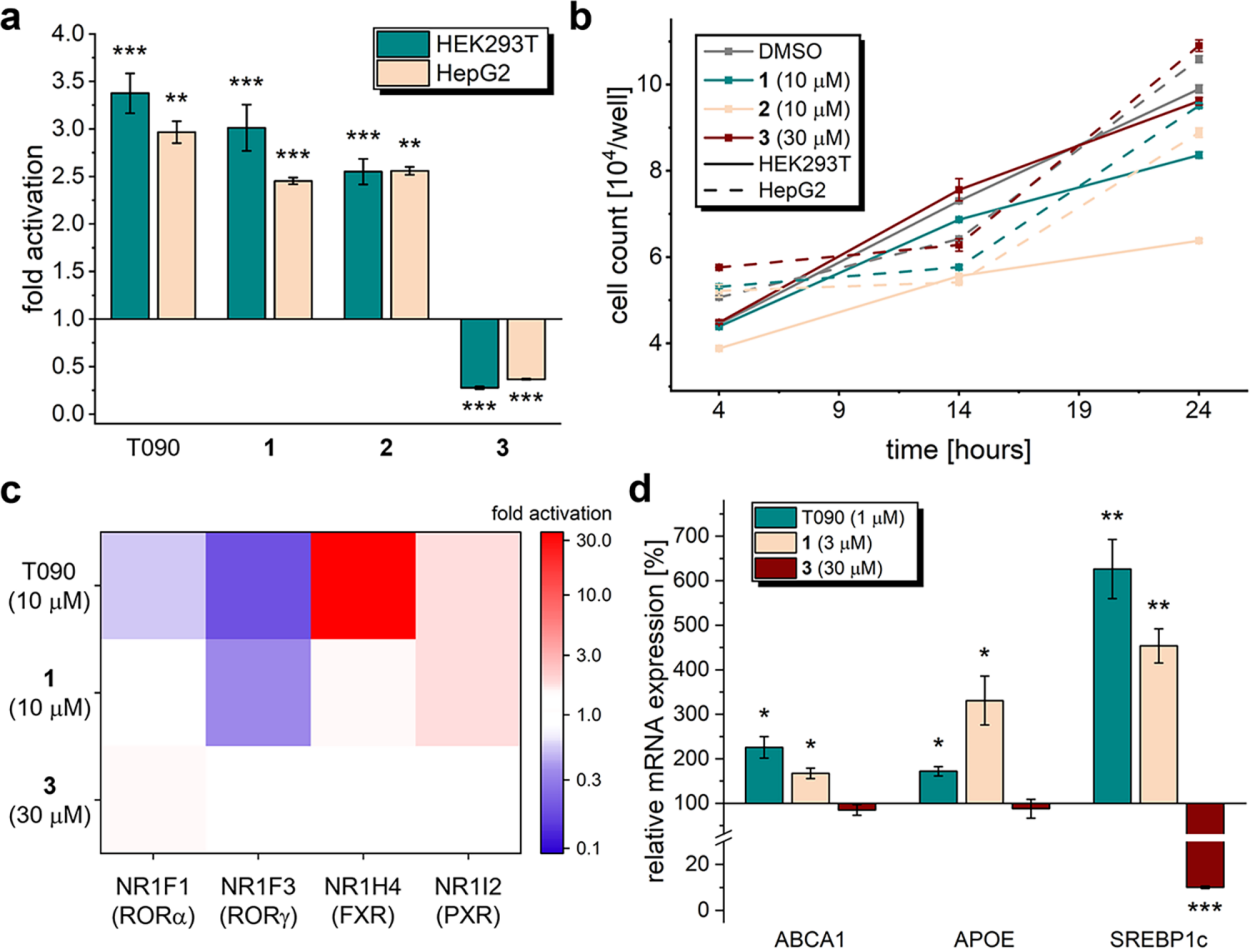
In vitro characterization of compounds **1**-**3**. (a) Effects of **1**-**3** in LXR-responsive ABCA1 reporter gene assays based on endogenous LXR protein levels in HEK293T and HepG2 cells. **1** and **2** displayed partial LXR agonism, whereas **3** was identified as an inverse agonist. The compounds were tested at 3 (**1**-**2**) or 10 μM (**3**). Data are expressed as mean ± SEM compared to the 0.1% DMSO control; T0901317 (T090, 1 μM) shown as reference; *N* = 3–6; ** *p* < 0.01, *** *p* < 0.001 (two-sided *t* test against 0.1% DMSO). (b) Designs **1**-**3** were not cytotoxic in a resazurin-based proliferation assay assessing metabolic activity of HEK293T and HepG2 cells after 4, 14, and 24 h. Data are the mean ± SEM; *N* = 3. (c) Off-target profiling of nuclear receptors known to be modulated by the promiscuous reference LXR agonist T090. **1** displayed improved selectivity compared to the structurally related T090, with no activity on RORα and FXR, and reduced activity on RORγ and PXR. **3** only activated RORα with low efficacy but was otherwise selective for LXRs. Data are the mean ± SEM; *N* = 3. (d) Quantification of LXR-regulated mRNA expression by qPCR in HepG2 cells after 24 h. Like the reference agonist T090 (1 μM), the partial agonist **1** (3 μM) induced LXR targets involved in reverse cholesterol transport (ABCA1, APOE) and lipogenesis (SREBP1c). Conversely, the inverse LXR agonist **3** (30 μM) repressed SREBP1c expression but did not significantly alter the expression of ABCA1 or APOE. Data are the mean ± SEM; *N* = 3–4; * *p* < 0.05, ** *p* < 0.01, *** *p* < 0.001 (two-sided *t* test against 0.1% DMSO).

Since several LXR modulators are known to exhibit off-target effects on a number of nuclear receptors, we also explored the selectivity profiles of the computational designs. Screening of the partial agonist **1** in Gal4 hybrid reporter gene assays for the known off-targets^35–37^ of the nonselective T0901317, RORα (NR1F1), RORγ (NR1F3), FXR (NR1H4), and PXR (NR1I2) revealed improved selectivity of **1**, despite the pronounced structural similarity (Figure 3c). Unlike T0901317, **1** did not affect RORα and FXR activity and was a weaker inverse RORγ agonist, while only PXR agonism was retained. The inverse LXR agonist **3** displayed weak RORα activation at 30 μM as the only off-target effect (Figure 3c).

For evaluation of LXR modulation by **1** and **3** in a more physiological setting, we determined their effects on the LXR-dependent expression of genes involved in reverse cholesterol transport (RCT), such as ABCA1 and apolipoprotein E (APOE), and in hepatic lipogenesis (SREBP1c). The reference LXR agonist T0901317 (1 μM) induced mRNA expression of all three genes in hepatocytes (HepG2; Figure 3d). The partial agonist **1** also stimulated ABCA1 and SREBP1c expression but to a lesser extent than the full agonist T0901317, while the induction of APOE exceeded the effect of T0901317. The inverse agonist **3** robustly suppressed SREBP1c (10% remaining mRNA expression) but, interestingly, had only a weak impact on RCT-related genes. LXR agonist-stimulated expression of genes involved in RCT holds therapeutic potential for diseases associated with elevated cholesterol levels such as atherosclerosis.^4^ However, the induction of hepatic lipogenesis via SREBP1c may be detrimental, as many patients with elevated cholesterol levels also suffer from other metabolic dysfunctions, and enhanced triglyceride synthesis in the liver can exacerbate hepatic steatosis. Conversely, the inhibition of LXR activity with inverse agonists may be beneficial for the treatment of MASLD by reducing lipogenesis but would suppress RCT, compromising therapeutic application. In this context, the partial agonist **1** and the inverse agonist **3** present attractive features. **1** mediated the induction of RCT genes without massive upregulation of SREBP1c as observed with the agonist T0901317, while **3** achieved robust SREBP1c suppression without detrimental effects on RCT genes.

In line with its effects on SREBP1c mRNA levels, the partial LXR agonist **1** led to the formation of less lipids in HepG2 cells than the structurally related full agonist T0901317 (Figure 4a,b), as confirmed by Oil Red O staining. In contrast, the inverse LXR agonist **3** did not induce lipid accumulation but significantly attenuated T0901317-induced lipogenesis. This histological experiment underscores the promising biological impact of inverse LXR agonism in MASLD. Therefore, we tested whether **3** also resolves existing lipid accumulation in a therapeutically more relevant approach. Indeed, design **3** displayed lipolytic, antisteatotic activity in HepG2 cells after steatosis induction by a 2:1 mixture of oleic acid and palmitic acid (Figure 4c,d). This promising phenotypic effect and the selective gene modulation by **3** may be valuable in MASLD, and its molecular basis deserves further attention. Nuclear receptor activity involves complex dimerization and coregulator interaction equilibria, which can respond differently to ligands. Subtle changes in the binding mode of a ligand may have impact on the recruitment of specific coregulators, which has been shown for other LXR ligands.^38^ Additionally, the expression pattern of coregulators can lead to tissue-specific ligand effects.^39^

**Figure 4:**
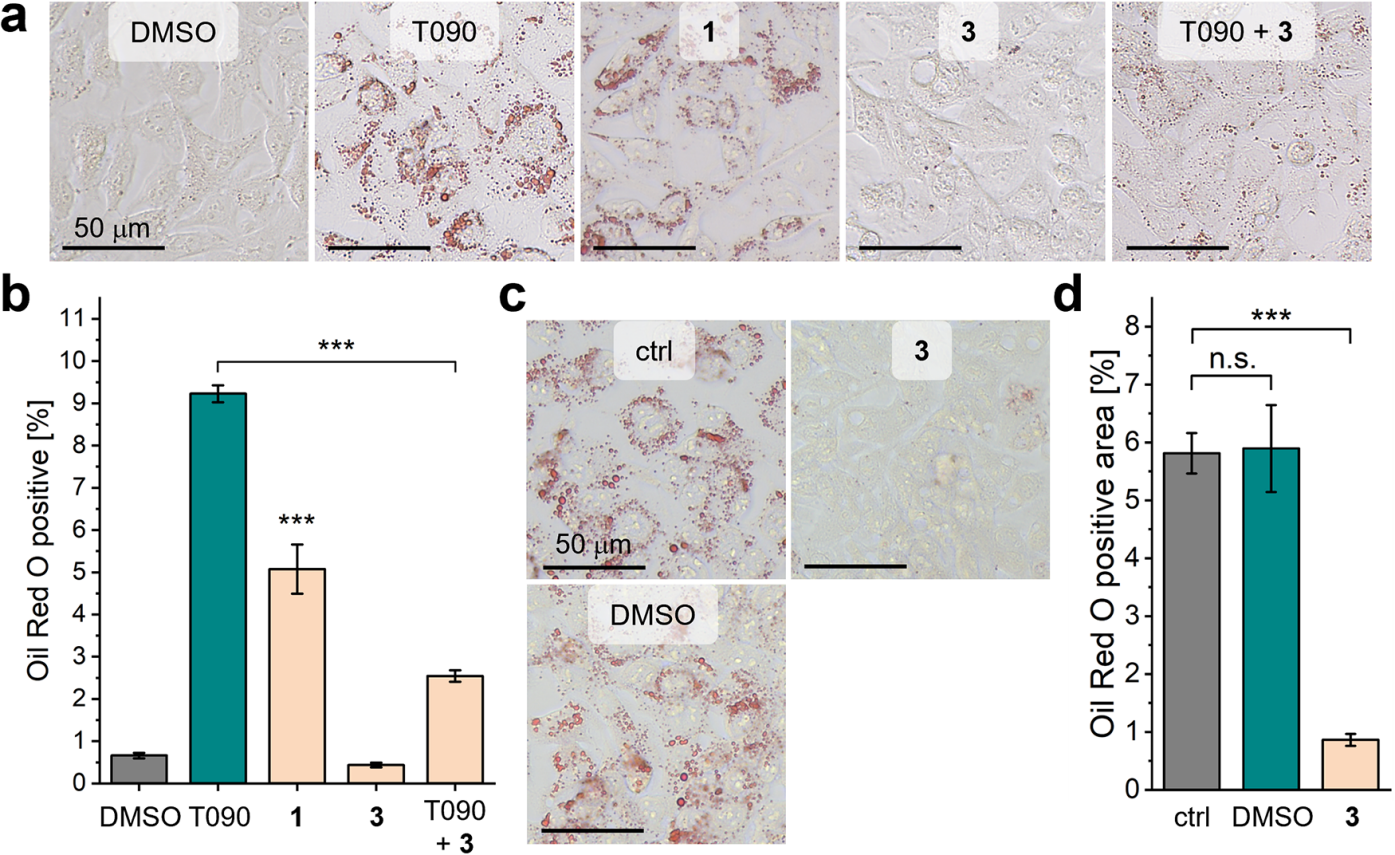
Lipogenic and lipolytic effects of the LXR modulators **1** and **3** in HepG2 cells. (a,b) Lipid accumulation assay in HepG2 cells (Oil Red O staining). Compared to LXR full agonist T0901317 (T090, 10 μM), **1** (10 μM) induced lipogenesis after 72 h less efficiently, whereas **3** (30 μM) did not stimulate lipid accumulation and mitigated T090-induced steatosis. Data in (b) are the mean ± SEM; *N* = 3; *** *p* < 0.001 (one-sided *t* test against 0.1% DMSO unless bars indicate differently). One representative from *N* = 3 displayed (complete set in Figure S4). (c,d) Compound **3** (30 μM, 72 h) stimulated lipolysis in HepG2 cells pretreated for 24 h with oleic acid (66.7 μM) and palmitic acid (33.3 μM) to induce steatosis (control). Data in (d) are the mean ± SEM; *N* = 3; *** *p* < 0.001 (one-sided *t* test against lipogenesis control). The images are one representative from *N* = 3 (complete set in Figure S5); ctrl, untreated control after steatosis induction.

## Conclusions

CLMs have shown promising potential in designing innovative new chemical entities with desired properties, including intended biological activities. The present explorative CLM application yielded novel LXR ligand scaffolds with attractive modulator profiles, underscoring the value of such models in drug design, e.g., by facilitating access to leads without the need to screen large compound libraries. The LXR ligands obtained in this study comprised structural features of different training templates and represented new chemotypes fusing existing structure–activity relationship data. In addition to a partial agonist analogue of the widely used reference agonist T0901317 with improved selectivity, this resulted in the discovery of an attractive inverse LXR agonist lead with a selective impact on target gene expression. In phenotypic settings, this LXR modulator displayed antilipogenic properties and resolved fatty acid-induced steatosis in a simple in vitro MASLD model. The optimization of its potency and physicochemical parameters may offer access to a new generation of LXR modulators and advance research on this promising metabolic target.

## Experimental Section

### Chemistry

#### General Chemistry

All chemicals and solvents were obtained from commercial sources of reagent grade and used without further purification. TLC was performed using TLC plates (silica gel 60 F254, 0.2 mm, Merck or Alugram Xtra Sil g/UV 0.2 mm, Macherey-Nagel) with detection under UV light (254 and 366 nm). Preparative column chromatography was performed using Silicagel 60 (Macherey-Nagel) and a solvent of technical grade. Reactions with air- or moisture-sensitive compounds were carried out under an argon atmosphere and in anhydrous solvents. NMR spectra were recorded on Bruker AM 250 XP, AV 300, AV 400, AV 500, or DRX600 spectrometers (Bruker Corporation, Billerica, MA, USA). Chemical shifts (δ) are reported in ppm relative to TMS, and coupling constants (*J*) in Hz. Multiplicity of signals is indicated as s for singlet, d for doublet, t for triplet, q for quartet, and m for multiplet. Aromatic signals resembling a triplet that stem from protons with two unequal neighbors but similar or equal coupling constants are denoted as doublet of doublets (dd). Mass spectra were obtained on a VG Platform II (Thermo Fischer Scientific, Inc., Waltham, MA, USA) using electrospray ionization (ESI). High-resolution mass spectra were recorded on a MALDI LTQ ORBITRAP XL instrument (Thermo Fisher Scientific). Accurate mass spectra were recorded with ESI on a Bruker MicrOTOF-Q II mass spectrometer (Bremen, Germany). Compound purity was analyzed on a Varian ProStar HPLC (SpectraLab Scientific Inc., Markham, ON, Canada) equipped with a MultoHigh100 Phenyl-5 μ 240 × 4 mm column (CS-Chromatographie Service GmbH, Langerwehe, Germany) using a gradient (H_2_O/MeOH 80:20 + 0.1% formic acid isocratic for 5 min to MeOH + 0.1% formic acid after additional 45 min and MeOH + 0.1% formic acid for additional 10 min) at a flow rate of 1 mL/min and UV detection at 254 and 280 nm. All final compounds for biological evaluation had a purity >95% according to the AUC at 254 and 280 nm UV detection.

#### 2-(4-[Ethylamino]­phenyl)-1,1,1,3,3,3-hexafluoropropan-2-ol (**6**)

Hexafluoroacetone hydrate (**5**, 332 μL, 2.38 mmol, 3.0 equiv) was added to a solution of *N*-ethylaniline (**4**, 100 μL, 795 μmol, 1.0 equiv) and *p*-toluenesulfonic acid (15 mg, 80 μmol, 0.10 equiv). The mixture was heated to 120 °C for 3 h according to a literature protocol.^40^ After the mixture was cooled to room temperature, the solvent was removed under reduced pressure. Purification by silica gel flash chromatography (10–20% EtOAc in hexane) afforded the product as a colorless solid (145 mg, 64%): *R*_f_ = 0.17 (hex/EtOAc 9:1); ^1^H NMR (400 MHz, CDCl_3_): δ_H_ 7.46 (d, *J* = 8.6 Hz, 2H), 6.66–6.56 (m, 2H), 3.76 (br s, 1H), 3.29 (br s, 1H), 3.18 (q, *J* = 7.2 Hz, 2H), 1.27 (t, *J* = 7.1 Hz, 3H); ^13^C NMR (101 MHz, CDCl_3_): δ_C_ 149.7, 127.7 (hep, *J* = 1.8 Hz), 123.0 (q, *J* = 287.0 Hz), 117.2, 112.3, 77.4, 38.3, 14.9; ^19^F NMR (377 MHz, CDCl_3_): δ_F_ −75.87; LRMS *m*/*z* (ESI) 288.08 [M + H]^+^.

#### *N*-Ethyl-*N*-(4-[1,1,1,3,3,3-hexafluoro-2-hydroxypropan-2-yl]­phenyl)­benzamide (**1**)

Triethylamine (86 μL, 0.62 mmol, 1.3 equiv) was added to a solution of **6** (137 mg, 476 μmol, 1.0 equiv) in 10 mL anhydrous CH_2_Cl_2_. The solution was cooled to 0 °C before adding benzoyl chloride (72 μL, 0.62 mmol, 1.3 equiv) dropwise. After complete addition, the reaction mixture was allowed to warm to room temperature and stirred for 2 h. The reaction was quenched with 5% HCl (aq.), 50 mL of EtOAc was added, and the product was washed with water (3 × 30 mL). The organic layer was dried over Na_2_SO_4_, filtered, and the solvents were evaporated in vacuo. The product was purified by flash silica gel column chromatography (20–33% EtOAc in hexane) and obtained as a colorless solid (124 mg, 67%): *R*_f_ = 0.23 (hex/EtOAc 4:1); ^1^H NMR (600 MHz, CDCl_3_): δ_H_ 7.58 (d, *J* = 8.5 Hz, 2H), 7.26–7.21 (m, 3H), 7.16–7.11 (m, 2H), 7.10–7.05 (m, 2H), 4.66 (s, 1H), 3.99 (q, *J* = 7.1 Hz, 2H), 1.23 (t, *J* = 7.1 Hz, 3H); ^13^C NMR (151 MHz, CDCl_3_): δ_C_ 170.5, 145.0, 135.7, 130.1, 128.8, 128.2, 127.9, 127.8, 127.7, 122.7 (q, *J* = 287.5 Hz), 77.1 (m), 45.6, 13.2; ^19^F NMR (282 MHz, CDCl_3_): δ_F_ −75.54; LRMS *m*/*z* (ESI) 392.00 [M + H]^+^, 413.99 [M + Na]^+^; HRMS *m*/*z* (MALDI) [Found: 392.10737, C_18_H_16_F_6_N_1_O_2_ requires [M + H]^+^ 392.10797].

#### *tert*-Butyl 4-(3-[Methylsulfonyl]­phenyl)­piperazine-1-carboxylate (**10**)

Anhydrous Cu­(OAc)_2_ (15 mg, 0.083 mmol, 0.1 equiv) and molecular sieves (4 Å) were added to a solution of (3-[methylsulfonyl]­phenyl)­boronic acid (**7**, 161 mg, 0.805 mmol, 1.5 equiv) and *tert*-butylpiperazine-1-carboxylate (**9**, 100 mg, 0.536 mmol, 1.0 equiv) in 7 mL of CH_2_Cl_2_. The resulting suspension was heated to 45 °C under reflux and was stirred for 16 h. Subsequently, water (15 mL) was added, and the organic components were extracted with EtOAc (3 × 15 mL). The combined organic layers were dried over MgSO_4_, and the solvents were evaporated under reduced pressure. The crude product was purified by flash silica gel column chromatography (50% EtOAc in hexane) to obtain a brown oil (71 mg, 0.21 mmol, 39%): *R*_f_ = 0.25 (hex/EtOAc 3:2); ^1^H NMR (300 MHz, CDCl_3_): δ_H_ 7.51–7.36 (m, 3H), 7.23–7.16 (m, 1H), 3.69–3.55 (m, 4H), 3.31–3.19 (m, 4H), 3.04 (s, 3H), 1.49 (s, 9H); ^13^C NMR (75 MHz, CDCl_3_): δ_C_ 154.8, 151.3, 141.7, 130.5, 121.1, 118.6, 114.4, 80.5, 49.0, 49.0, 44.6, 28.5; LRMS *m*/*z* (ESI) 362.96 [M + Na]^+^.

#### 4-(3-[Methylsulfonyl]­phenyl)­piperazin-1-ium Trifluoroacetate (**11**)

Trifluoroacetic acid (1 mL) was added to a solution of *tert*-butyl 4-(3-[methylsulfonyl]­phenyl)­piperazine-1-carboxylate (**10**, 71 mg, 0.21 mmol, 1.0 equiv) in CH_2_Cl_2_ (3 mL) and stirred at room temperature for 2 h. The solvents were evaporated to give the product as a brown oil (74 mg, 99%): *R*_f_ = 0.13 (CH_2_Cl_2_/MeOH 9:1); ^1^H NMR (300 MHz, DMSO-*d*_6_): δ_H_ 8.95 (s, 2H), 7.51 (dd, *J* = 8.0, 8.0 Hz, 1H), 7.47–7.40 (m, 1H), 7.40–7.29 (m, 2H), 3.55–3.40 (m, 4H), 3.33–3.14 (m, 7H); ^13^C NMR (75 MHz, DMSO-*d*_6_): δ_C_ 158.2 (m), 150.3, 141.8, 130.2, 120.4, 117.4, 115.2 (m), 113.1, 45.0, 43.4, 42.6; LRMS *m*/*z* (ESI) 241.14 [M-CF_3_COO]^+^.

#### 1-(4-Chloro-3-[trifluoromethyl]­phenyl)-4-(3-[methylsulfonyl]­phenyl)­piperazine (**2**)

A mixture of **11** (68 mg, 0.28 mmol, 1.0 equiv), 4-chloro-3-(trifluoromethyl)­phenylboronic acid (**12**, 95 mg, 0.42 mmol, 1.5 equiv), anhydrous Cu­(OAc)_2_ (51 mg, 0.28 mmol, 1.0 equiv), pyridine (68 μL, 0.85 mmol, 3.0 equiv), and molecular sieves (4 Å) in dry CH_2_Cl_2_ (8 mL) was heated to 45 °C under reflux and exclusion of water for 16 h. After cooling to room temperature, the mixture was acidified with 5% HCl, brine (30 mL) was added, and the organic components were extracted with CH_2_Cl_2_ (3 × 30 mL). The combined organic layers were dried over Na_2_SO_4_, and the solvents were evaporated in vacuo. Purification by flash silica gel chromatography (40–60% EtOAc in hexane) afforded the product as a yellow solid (25 mg, 21%): *R*_f_ = 0.38 (hex/EtOAc 1:1); ^1^H NMR (500 MHz, CDCl_3_): δ_H_ 7.50–7.43 (m, 2H), 7.41 (ddd, *J* = 7.7, 1.3, 1.3 Hz, 1H), 7.37 (d, *J* = 8.8 Hz, 1H), 7.22 (d, *J* = 3.0 Hz, 1H), 7.18 (dd, *J* = 8.1, 2.2 Hz, 1H), 7.02 (d, *J* = 8.9, 3.0 Hz, 1H), 3.48–3.40 (m, 4H), 3.40–3.32 (m, 4H), 3.06 (s, 3H); ^13^C NMR (126 MHz, CDCl_3_): δ_C_ 151.5, 149.5, 141.7, 132.2, 130.5, 128.9 (q, *J* = 30.9 Hz), 123.1 (q, *J* = 273.4 Hz), 122.3 (q, *J* = 2.0 Hz), 120.6, 120.0, 118.1, 115.0 (q, *J* = 5.5 Hz), 114.0, 48.8, 48.5, 44.6; ^19^F NMR (471 MHz, CDCl_3_): δ_F_ −62.67; LRMS *m*/*z* (ESI) 440.87 [M + Na]^+^; Accurate mass *m*/*z* (ESI) [Found: 419.0806, C_18_H_19_ClF_3_N_2_O_2_S requires [M + H]^+^ 419.0802].

#### (3′-[Methylsulfonyl]-[1,1′-biphenyl]-4-yl)­methanol (**14**)

A two-neck flask was charged with (3-[methylsulfonyl]­phenyl)­boronic acid (**7**, 259 mg, 1.30 mmol, 1.2 equiv), (4-bromophenyl)­methanol (**13**, 202 mg, 1.08 mmol, 1.0 equiv), tetrakis­(triphenylphosphine)­palladium(0) (63 mg, 54 μmol, 0.05 equiv), and sodium carbonate (343 mg, 3.24 mmol, 3.0 equiv). 1,4-Dioxane (6.0 mL) and water (1.5 mL) were added under argon, and the solvents were degassed. The mixture was heated to 110 °C under reflux for 2 h. After the mixture cooled to room temperature, brine (30 mL) was added, and the organic components were extracted with EtOAc (3 × 40 mL). The combined organic layers were dried over Na_2_SO_4_, and the solvent was evaporated under reduced pressure. The crude product was purified by flash silica gel column chromatography (50–60% EtOAc in hexane) to obtain a yellow solid (275 mg, 97%): *R*_f_ = 0.22 (hex/EtOAc 1:1); ^1^H NMR (300 MHz, CDCl_3_): δ_H_ 8.13 (s, 1H), 7.93–7.79 (m, 2H), 7.67–7.55 (m, 3H), 7.46 (d, *J* = 8.0 Hz, 2H), 4.74 (s, 2H), 3.08 (s, 3H), 2.18 (s, 1H); ^13^C NMR (75 MHz, CDCl_3_): δ_C_ 142.5, 141.4, 141.2, 138.2, 132.2, 130.0, 127.7, 127.4, 126.0, 125.8, 64.8, 44.6; LRMS *m*/*z* (ESI) 285.00 [M + Na]^+^.

#### 4′-(Bromomethyl)-3-(methylsulfonyl)-1,1′-biphenyl (**15**)

Phosphorus tribromide (286 μL, 1.57 mmol, 1.5 equiv) was added to a solution of **14** (275 mg, 1.05 mmol, 1.0 equiv) in CH_2_Cl_2_ (8.0 mL) at 0 °C and stirred in an argon atmosphere for 2 h. Water (2 mL) was added to quench the reaction. EtOAc (30 mL) was added, and the organic layer was washed with saturated NaHCO_3_ solution (3 × 30 mL). The organic layer was dried over Na_2_SO_4_, and the solvents were evaporated under reduced pressure. The crude product was purified by flash silica gel column chromatography (25–33% EtOAc in hexane) to obtain the product as an off-white solid (298 mg, 87%): *R*_f_ = 0.26 (hex/EtOAc 3:1); ^1^H NMR (300 MHz, CDCl_3_): δ_H_ 8.14 (s, 1H), 7.92 (d, *J* = 7.8 Hz, 1H), 7.85 (d, *J* = 7.8 Hz, 1H), 7.69–7.54 (m, 3H), 7.49 (d, *J* = 8.1 Hz, 2H), 4.54 (s, 2H), 3.09 (s, 3H); ^13^C NMR (75 MHz, CDCl_3_): δ_C_ 142.0, 141.4, 139.1, 138.1, 132.2, 130.0, 129.9, 127.7, 126.3, 125.8, 44.6, 33.0; LRMS *m*/*z* (ESI) 346.84 [M + Na]^+^, 348.76 [M + Na]^+^.

#### *N*-([3′-{Methylsulfonyl}-{1,1′-biphenyl}-4-yl]­methyl)-2,2-diphenylethan-1-amine (**17**)

A mixture of **15** (290 mg, 890 μmol, 1.0 equiv), 2,2-diphenylethanamine (**16**, 194 mg, 979 μmol, 1.1 equiv), and potassium carbonate (246 mg, 1.78 mmol, 2.0 equiv) in dry DMF (7.0 mL) was stirred at room temperature for 2 h. EtOAc (30 mL) was added, and the organic layer was washed with brine (3 × 30 mL). The organic layer was dried over Na_2_SO_4_, and the solvents were evaporated under reduced pressure. The crude product was purified by flash silica gel column chromatography (50–67% EtOAc in hexane) to obtain the product as a pale yellow oil (258 mg, 66%): *R*_f_ = 0.15 (hex/EtOAc 1:1); ^1^H NMR (300 MHz, CDCl_3_): δ_H_ 8.16 (d, *J* = 1.6 Hz, 1H), 7.96–7.81 (m, 2H), 7.63 (dd, *J* = 7.8, 7.8 Hz, 1H), 7.56 (d, *J* = 7.8 Hz, 2H), 7.41–7.16 (m, 12H), 4.25 (t, *J* = 7.6 Hz, 1H), 3.89 (s, 2H), 3.27 (d, *J* = 7.6 Hz, 2H), 3.09 (s, 3H); ^13^C NMR (75 MHz, CDCl_3_): δ_C_ 142.9, 142.6, 141.3, 140.7, 137.7, 132.2, 130.0, 128.9, 128.7, 128.1, 127.3, 126.7, 125.9, 125.8, 53.9, 53.4, 51.4, 44.7; LRMS *m*/*z* (ESI) 441.97 [M + H]^+^.

#### *N*-(2-Chloro-3-[trifluoromethyl]­benzyl)-*N*-([3′-{methylsulfonyl}-{1,1′-biphenyl}-4-yl]­methyl)-2,2-diphenylethan-1-amine (**3**)

A solution of **17** (249 mg, 563 μmol, 1.0 equiv), 2-chloro-3-(trifluoromethyl)­benzaldehyde (**18**, 129 mg, 620 μmol, 1.1 equiv), and acetic acid (65 μL, 1.1 mmol, 2.0 equiv) in 1,2-dichloroethane (7.0 mL) was stirred at room temperature for 2 h. Sodium triacetoxyborohydride (164 mg, 620 μmol, 2.0 equiv) was added, and the mixture was stirred for a further 16 h. More sodium triacetoxyborohydride (164 mg, 620 μmol, 2.0 equiv) was added, and the mixture was stirred for another hour, after which, the reaction was quenched by the addition of water (5 mL). EtOAc (30 mL) was added, and the organic layer was washed with saturated NaHCO_3_ (3 × 30 mL). The organic layer was then dried over Na_2_SO_4_, and the solvents were evaporated under reduced pressure. The crude product was purified by flash silica gel column chromatography (25–100% EtOAc in hexane), followed by reversed-phase flash silica gel column chromatography (5–95% (MeCN + 2% HOAc) in (H_2_O + 2% HOAc)) to obtain the product as a pale yellow oil (116 mg, 32%): *R*_f_ = 0.38 (hex/EtOAc 3:1); ^1^H NMR (500 MHz, CDCl_3_): δ_H_ 8.14 (dd, *J* = 1.6, 1.6 Hz, 1H), 7.91 (ddd, *J* = 7.8, 1.6, 1.6 Hz, 1H), 7.85 (ddd, *J* = 7.8, 1.6, 1.6 Hz, 1H), 7.64 (dd, *J* = 7.8, 7.8 Hz, 1H), 7.53 (dd, *J* = 7.8, 1.7 Hz, 1H), 7.49 (d, *J* = 7.8 Hz, 2H), 7.32–7.18 (m, 9H), 7.18–7.08 (m, 5H), 4.26 (t, *J* = 7.8 Hz, 1H), 3.82 (s, 2H), 3.76 (s, 2H), 3.17 (d, *J* = 7.9 Hz, 2H), 3.10 (s, 3H); ^13^C NMR (126 MHz, CDCl_3_): δ_C_ 143.2, 142.6, 141.4, 139.6, 139.4, 137.9, 133.9, 132.2, 131.6, 130.0, 129.7, 128.7, 128.50, 128.45, 127.1, 126.6, 126.4, 126.1 (q, *J* = 5.8 Hz), 126.0, 125.9, 123.2 (q, *J* = 273.0 Hz), 60.1, 58.9, 55.5, 49.7, 44.7; ^19^F NMR (471 MHz, CDCl_3_): δ_F_ −62.24; LRMS *m*/*z* (ESI) 633.88 [M + H]^+^; Accurate mass *m*/*z* (ESI) [Found: 634.1811, C_36_H_32_ClF_3_NO_2_S requires [M + H]^+^ 634.1789].

### Biological Evaluation

#### Tissue Culture

HEK293T and HepG2 cells (German Collection of Microorganisms and Cell Cultures, DSMZ) were grown in Dulbecco’s modified Eagle’s medium (DMEM) high glucose supplemented with 10% fetal bovine serum (FBS), sodium pyruvate (1 mM), penicillin (100 U/mL), and streptomycin (100 μg/mL) at 37 °C and 5% CO_2_.

#### Plasmids

For the hybrid reporter gene assays, the previously reported Gal4-fusion receptor plasmids pFA-CMV-hRORα-LBD,^19^ pFA-CMV-hRORβ-LBD,^19^ pFA-CMV-hRORγ-LBD,^19^ pFA-CMV-hLXRα-LBD,^28^ pFA-CMV-hLXRβ-LBD,^28^ pFA-CMV-hFXR-LBD,^41^ pFA-CMV-hPXR-LBD,^42^ and pFA-CMV-hRXRα-LBD,^42^ coding for the hinge region, and LBD of the canonical isoform of the respective nuclear receptor were used. pFR-Luc (Stratagene) was used as the reporter plasmid, and pRL-SV40 (Promega) was used in all assays for the normalization of transfection efficiency and cell growth. pECE-SV40-Gal4-VP16 ^54^ was a gift from Lea Sistonen (Addgene plasmid #71728). The LXR-responsive reporter ABCA1-pGL3 ^34^ was used for the full-length reporter gene assay.

#### Reporter Gene Assays

The day before transfection, HEK293T cells were seeded in 96-well plates (4 × 10^4^ cells/well). Before transfection, the medium was changed to Opti-MEM without any supplements. Transient transfection was carried out using Lipofectamine LTX reagent (Invitrogen) according to the manufacturer’s protocol with (a) pFR-Luc (Stratagene), pRL-SV40 (Promega), and the corresponding Gal4-fusion nuclear receptor plasmid or (b) the LXR-responsive reporter ABCA1-pGL3 and pRL-SV40 (Promega).^34^ Five hours after transfection, the medium was changed to Opti-MEM supplemented with penicillin (100 U/mL) and streptomycin (100 μg/mL), and now additionally containing 0.1% DMSO and the respective test compound or 0.1% DMSO alone as untreated control. Each concentration was tested in duplicate, and each experiment was repeated in at least three biologically independent repeats. Following overnight (14–16 h) incubation with the test compounds, cells were assayed for luciferase activity using a Dual-Glo Luciferase Assay System (Promega) according to the manufacturer’s protocol. Luminescence was measured with a Spark 10 M luminometer (Tecan Deutschland GmbH). Normalization of transfection efficiency and cell growth was done by division of firefly luciferase data by renilla luciferase data multiplied by 1000, resulting in relative light units (RLU). Fold activation was obtained by dividing the mean RLU of the tested compound at a respective concentration by the mean RLU of the untreated 0.1% DMSO control. Relative activation was obtained by dividing the fold activation of the tested compound at a respective concentration by the fold activation of the respective reference agonist (RORα: 10 μM SR1001, RORγ: 1 μM SR1001, LXRα/β: 1 μM T0901317, FXR: 1 μM GW4064, PXR: 1 μM SR12813, and RXRα: 1 μM bexarotene). Separate control experiments to exclude nonspecific reporter gene activation or VP16-mediated effects were performed following the same procedure in the absence of a nuclear receptor expression plasmid (pFR-Luc, pRL-SV40, and pECE-SV40-Gal4-VP16 only). EC_50_/IC_50_ values and the standard errors thereof were calculated with the mean fold activation values by OriginPro, Version 2025 (OriginLab Corporation, Northampton, MA, USA), fitting a dose–response curve with a variable Hill slope (Levenberg–Marquardt algorithm). All assays were validated with the reference agonists mentioned above, which yielded EC_50_/IC_50_ values in agreement with the literature.

#### Metabolic Stability Assay

The dissolved test compound (5 μL, final concentration 10 μM) was preincubated at 37 °C in 432 μL of phosphate buffer (0.1 M, pH 7.4) together with a 50 μL NADPH regenerating system (30 mM glucose-6-phosphate, 4 U/mL glucose-6-phosphate dehydrogenase, 10 mM NADP, and 30 mM MgCl_2_). After 5 min, the reaction was started by the addition of 13 μL of microsome mix from the liver of Sprague–Dawley rats (Invitrogen; 20 mg protein/mL in 0.1 M phosphate buffer) in a shaking water bath at 37 °C. The reaction was stopped by the addition of 500 μL of ice-cold methanol at 0, 15, 30, and 60 min. The samples were centrifuged at 5000 *g* for 5 min at 4 °C. The supernatants were analyzed, and the test compound was quantified by HPLC: The composition of the mobile phase is adapted tot the test compound in a range of 40-90% acetonitrile and 10-60% water (0.1% formic acid); flow rate: 1 mL/min; stationary phase: PurospherⓇ STAR, RP18, 5 μm, 125 × 4, precolumn: PurospherⓇ STAR, RP18, 5 μm, 4 × 4; detection wavelength: 254 and 280 nm; injection volume: 50 μL. Control samples were performed to check the test compound’s stability in the reaction mixture: the first control was without NADPH, which is needed for the enzymatic activity of the microsomes, the second control was with inactivated microsomes (incubated for 20 min at 90 °C), and the third control was without test compound (to determine the baseline). The amounts of the test compound were quantified by an external calibration curve. Data are expressed as the mean ± SEM remaining compound from three independent experiments.

#### Proliferation Assay

HepG2 or HEK293T cells were seeded in 96-well plates (4 × 10^4^ cells per well). After 24 h, the cells were incubated with the test compounds dissolved in culture medium with 0.1% DMSO or medium with 0.1% DMSO alone as untreated control. After 4, 14, and 24 h, the medium was aspirated, and the cells were incubated with 100 μM resazurin solution in Opti-MEM (prepared from a 0.15 mg/mL resazurin stock in DPBS) for 60 min. The metabolic activity was assessed by conversion of resazurin to resorufin, which was quantified by measuring absorbance at 570 nm and a reference wavelength of 600 nm. The cell count was calculated from a calibration curve with 0–10^5^ cells per well in steps of 2 × 10^4^.

#### Target Gene Quantification

HepG2 cells were seeded in six-well plates (1 × 10^6^ per well). After 24 h, the medium was changed to Opti-MEM supplemented with 1% charcoal-stripped fetal bovine serum (FBS), penicillin (100 U/mL), streptomycin (100 μg/mL), and l-glutamine (2 mM). After an additional 24 h, cells were incubated with the test compounds dissolved in the same medium with 0.1% DMSO or medium with 0.1% DMSO alone as untreated control for 24 h, harvested, washed with cold phosphate-buffered saline (PBS), and then directly used for the next step. Total RNA was extracted from HepG2 cells using the total RNA Mini Kit (R6834–02, Omega Bio-Tek, Inc., Norcross, GA, USA), and the same amount of RNA per sample was then reverse-transcribed into cDNA with the High-Capacity cDNA Reverse Transcription Kit (4368814, Thermo Fischer Scientific, Inc.). LXR-regulated gene expression was then studied by quantitative real-time PCR (qRT-PCR) analysis with a StepOnePlus System (Life Technologies, Carlsbad, CA, USA) using PowerSYBRGreen (Life Technologies). Each sample was set up in duplicates and repeated in four independent experiments. Data were analyzed by the comparative ΔΔ*c_t_* method with glyceraldehyde 3-phosphate dehydrogenase (GAPDH) as the reference gene. The following primers were used (human genes): hGAPDH fwd: 5′-ATA TGA TTC CAC CCA TGG CA-3′, rev: 5′-GAT GAT GAC CCT TTT GGC TC-3′; hABCA1 fwd: 5′-TTC GCT CTG AGA TGA GCA CCA-3′, rev: 5′-TTT CAA GCG GGC ATA GAA CCA-3′; hAPOE fwd: 5′-TTC GCT CTG AGA TGA GCA CCA-3′, rev: 5′-TTT CAA GCG GGC ATA GAA CCA-3′; hLXRa fwd: 5′-TGG ACA CCT ACA TGC GTC GCA A-3′, rev: 5′-CAA GGA TGT GGC ATG AGC CTG T-3′, hLXRb fwd: 5′-CTT CGC TAA GCA AGT GCC TGG T-3′, rev: 5′-CAC TCT GTC TCG TGG TTG TAG C-3′; hSREBP1c fwd: 5′-GGA GGG GTA GGG CCA ACG GCC T-3′, rev: 5′-CAT GTC TTC GAA AGT GCA ATC C-3′.

#### Lipid Accumulation Assay

HepG2 cells were seeded in collagen-coated 96-well plates at a density of 10^4^ cells per well and incubated for 24 h. For the lipolytic mode, the cells were pretreated for additional 24 h with 66.7 μM oleic acid and 33.3 μM palmitic acid in culture medium with 1% bovine serum albumin (BSA). The cells were then treated with DMSO (0.1%) alone or in combination with test compounds in the culture medium. After 72 h, the cells were washed with PBS (2 × 50 μL) and fixed with 10% formalin solution (50 μL) for 15 min. After removal of the fixative, the cells were washed with H_2_O (2 × 50 μL), followed by 50 μL of 60% *i*PrOH and incubated with 50 μL Oil Red O solution (0.3% in 60% *i*PrOH) for 10 min to stain neutral lipids. The cells were washed quickly with 50 μL of 60% *i*PrOH and 2 × 50 μL of PBS. Microscopic images were taken using a 40x objective on a BZ-X810 microscope (KEYENCE, Frankfurt, Germany). Images were analyzed using ImageJ software (v1.54) according to a literature method.^43^

### Computational Methods

#### Software

Computations were performed using Python (v3.9.10) with the following packages: RDKit (v2021.09, DOI 10.5281/zenodo.591637), TensorFlow (v2.9.0, DOI 10.5281/zenodo.4724125), scikit-learn (v1.4.2 ^44^), matplotlib (v3.8.3 ^45^), and seaborn (v0.13.2 ^46^).

#### Data Processing

Molecules were encoded as canonical SMILES using RDKit (v2021.09, www.rdkit.org) and standardized in Python (v3.9.10, www.python.org) by removing isotopes, stereochemistry, salts, and duplicates. SMILES strings exceeding 140 characters were excluded from the data set.

#### Fingerprints

Morgan fingerprints (radius 2, 2048-bit)^47^ were computed with RDKit (v2021.09, www.rdkit.org).

#### Data Sets

For t-SNE projection background and similarity analysis, we used a previously published data set^19^ derived from ChEMBL (v24 ^48^), comprising 365,063 molecules. This data set was also employed for pretraining the chemical language model. For the first fine-tuning round, 252 LXR modulators were extracted from BindingDB (v2022.01 ^49^). We selected all compounds annotated with EC_5__0_, IC_5__0_, or *K*_d_ values below 1 μM and with “*Homo sapiens*” listed as the source organism (fine-tuning set I, Table S3). The second fine-tuning round was performed with 12 selected LXR modulators (fine-tuning set II, Table S4).

#### Chemical Language Model

We used a previously published pretrained model^19^ as the base CLM. The CLM was implemented in Python (v3.9.7) using TensorFlow (v2.9.0) and the Keras API (www.keras.io). The model architecture included six layers totaling 8,444,003 parameters: layer 1: BatchNormalization, layer 2: LSTM^14^ (1024 units), layer 3: LSTM (512 units), layer 4: LSTM (256 units), layer 5: BatchNormalization, and layer 6: Dense (71 units). The CLMs were trained on SMILES using the Adam optimizer^50^ (pretraining learning rate = 10^–3^) and categorical cross-entropy loss.

#### Fine-Tuning

Fine-tuning was conducted for 20 epochs using a learning rate of 10^–^^4^ and a batch size of 8. A learning rate reduction on plateau (factor 0.5, patience 3, and minimum learning rate 5 × 10^–^^5^) was applied. Each LSTM layer included a dropout^51^ rate of 0.4. During the first fine-tuning stage, the LSTM layer was frozen. In the second stage, both the first and second LSTM layers were frozen. 10-fold SMILES augmentation^52^ was applied during both stages. The first fine-tuning round was conducted on the fine-tuning set I, and the second round was conducted on the fine-tuning set II.

#### Epoch Selection and Beam Search

To select epoch checkpoints for downstream tasks, representative molecules were sampled using a beam search at each epoch and compared to the fine-tuning set II using Tanimoto similarity on Morgan fingerprints. Beam search was conducted with a beam width of *k* = 50. At each time step *t*, the algorithm retained the top-*k* partial sequences *y*_1:*t*_, ranked by their cumulative log-probability:

$$\mathrm{score}\left(y_{1:t}\right)=\overset{t}{\underset{i=1}{\sum }}\log\;P\left(y_{i}|y_{1:i-1}\right)$$

where *y_i_* is the *i*-th token in the output sequence. No length normalization was applied. Generation continued until a complete SMILES was formed, or the maximum length of 140 characters was reached. For the second stage of fine-tuning, the checkpoint from epoch 19 of the first fine-tuning round was used. For temperature-based sampling, checkpoints from epochs 5–12 of the second fine-tuning stage were selected.

#### Temperature Sampling

SMILES were sampled with a maximum length of 140 characters using softmax sampling with temperature *T* = 0.2. The probability of the *i*-th character to be sampled from the CLM was calculated as

$$q_{i}=\frac{\exp\left(\frac{z_{i}}{T}\right)}{\underset{j}{\sum }\exp\left(\frac{z_{j}}{T}\right)}$$

where *z*_*i*_ is the model output (logit) for character *i*, and *q*_*i*_ is the sampling probability of character *i*. For each selected epoch from the second fine-tuning round, 2000 molecules were sampled.

#### Molecular Docking

Ligand docking simulations were performed using smina (v2021.12.01 ^25^). Ligands and protein structures were prepared using obabel,^53^ including the addition of hydrogens and generation of 3D coordinates. The ligand-bound X-ray structures of LXRα (PDB ID: 3IPQ^23^) and LXRβ (PDB ID: 5JY3 ^24^) were used as templates. The docking grid was defined as an autobox around the bound ligand with a box size of 8 and an exhaustiveness of 16. The final score was computed as the geometric mean of the docking scores for LXRα and LXRβ. Redocking (5 repeats) of the cocrystallized ligands resulted in high scores and low RMSD (LXRβ (5JY3): score = −15.1 ± 0.0, RMSD = 1.528 ± 0.004; LXRα (3IPQ) = −14.0 ± 0.0, RMSD = 0.706 ± 0.057).

#### Stochastic Neighbor Embedding

The t-SNE projection was performed with scikit-learn (v1.4.2 ^44^) in Python (v3.9.7). Morgan fingerprints were first reduced to 50 dimensions using Truncated SVD. The Jaccard distance was used as the distance metric. The t-SNE projection used a perplexity of 30, with all other parameters left at their default values.

#### Virtual Screening Simulation

The Enamine screening collection (>4.6 million compounds, August 2025, enamine.net) was processed with RDKit (v2021.09, www.rdkit.org), excluding 29,330 entries that could not be parsed into valid molecular structures. The de novo designs **1–3** were appended to the processed library. Molecular similarities were computed as Tanimoto similarity based on Morgan fingerprints between fine-tuning set I and the augmented library. For each query molecule, the top 1000 most similar compounds were retained, and a consensus ranking was obtained by averaging the ranks across all queries.

## Supplementary Material





## Notes

Dose-response curves, control experiments, designs **1**-**3** and comparison to known LXR ligands, top designs by frequency and docking, fine-tuning sets, NMR spectra and HPLC traces (PDF)
Molecular formula strings of **1**-**3** and associated activity data (CSV)

## Abbreviations

ABCA1: ATP-binding cassette transporter A1
CLM: chemical language model
DIPEA: *N*,*N*-diisopropylethylamine
DMEM: Dulbecco’s modified Eagle’s medium
EtOAc: ethyl acetate
FBS: fetal bovine serum
FXR: farnesoid X receptor
GAPDH: glyceraldehyde 3-phosphate dehydrogenase
LXR: liver X receptor
LSTM: long short-term memory
MASLD: metabolic dysfunction-associated steatotic liver disease
PXR: pregnane X receptor
RCT: reverse cholesterol transport
RLU: relative light units
ROR: retinoic acid receptor-related orphan receptor
RXR: retinoid X receptor
SEM: standard error of the mean
SMILES: Simplified Molecular Input Line Entry System
SNE: stochastic neighbor embedding
SREBP1c: sterol-regulatory element-binding protein 1c
