# Supplementary material for: Scaffold Fusion and SAR Transfer with a Chemical Language Model Generates Novel Liver X Receptor Modulators
Source: J Med Chem. 2025 Oct 23;68(21):22588–98. doi: 10.1021/acs.jmedchem.5c01551 (PMC12621175; doi:10.1021/acs.jmedchem.5c01551)
Supplement: Supplementary file 1 [file jm5c01551_si_001.pdf]

Supporting Information

Scaffold fusion and SAR transfer with a chemical language model generates novel liver X receptor modulators

Nils Christiaan Bandomir[1], Tim Hörmann[2], Annette Kärcher[1], Astrid Kaiser[1], Daniel Merk[2]*, and Pascal Heitel[1]*

[1] Institute of Pharmaceutical Chemistry, Goethe University Frankfurt, Max-von-Laue-Strasse 9, 60438 Frankfurt, Germany
[2] Department of Pharmacy, Ludwig-Maximilians-Universität (LMU) München, Butenandtstrasse 5-13, 81377 Munich, Germany

*Daniel Merk, email: daniel.merk@cup.lmu.de,
Pascal Heitel, email: heitel@pharmchem.uni-frankfurt.de

Table of Contents

1. Supporting Figures and Charts S3

2. Supporting Tables S8

3. NMR Spectra & HPLC Chromatograms S22

2-(4-[Ethylamino]phenyl)-1,1,1,3,3,3-hexafluoropropan-2-ol (**6**) S22

N-Ethyl-*N*-(4-[1,1,1,3,3,3-hexafluoro-2-hydroxypropan-2-yl]phenyl)benzamide (**1**) S24

tert-Butyl 4-(3-[methylsulfonyl]phenyl)piperazine-1-carboxylate (**10**) S27

4-(3-[Methylsulfonyl]phenyl)piperazin-1-ium trifluoroacetate (**11**) S28

1-(4-Chloro-3-[trifluoromethyl]phenyl)-4-(3-[methylsulfonyl]phenyl)piperazine (**2**) S29

(3'-[Methylsulfonyl]-[1,1'-biphenyl]-4-yl)methanol (**14**) S32

4'-(Bromomethyl)-3-(methylsulfonyl)-1,1'-biphenyl (**15**) S33

N-([3'-{Methylsulfonyl}-{1,1'-biphenyl}-4-yl]methyl)-2,2-diphenylethan-1-amine (**17**) S34

N-(2-Chloro-3-[trifluoromethyl]benzyl)-*N*-([3'-{methylsulfonyl}-{1,1'-biphenyl}-4-yl]methyl)-2,2-diphenylethan-1-amine (**3**) S35

4. Supplementary References S38

1. Supporting Figures and Charts

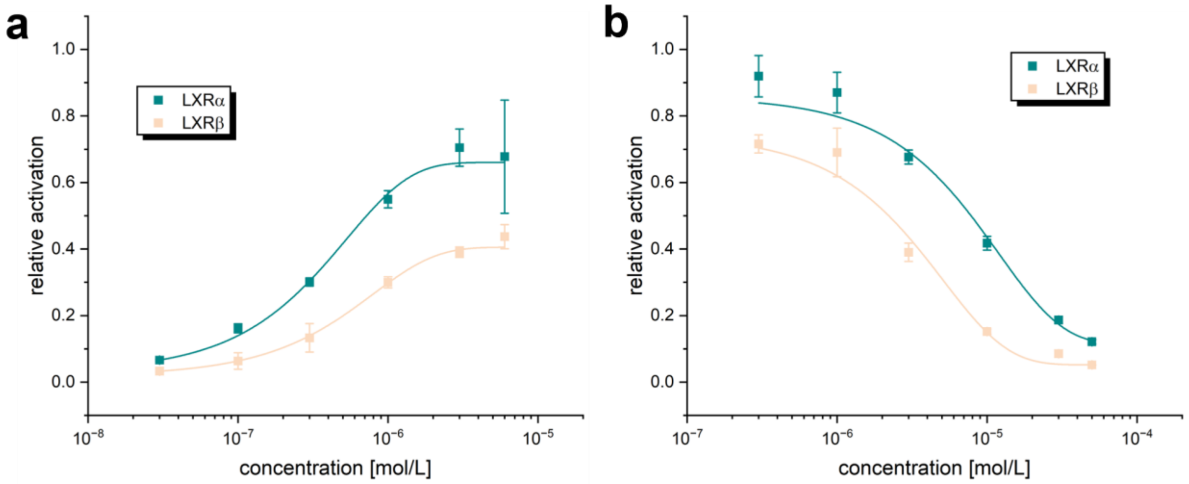

Figure S1. Dose-response curves for compounds **1** (a) and **3** (b) in the LXR-Gal4 hybrid reporter gene assay. Compound **3** was tested competitively against 1 µM reference agonist T0901317. Data are the mean ± standard error of the mean (SEM) from N ≥ 3. The activation of 1 µM reference agonist T0901317 was defined as 1.

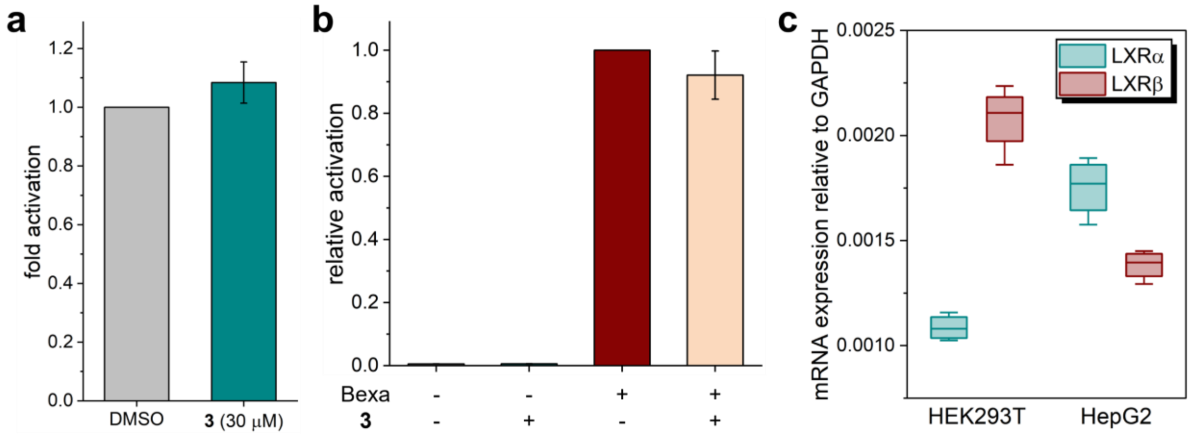

Figure S2. Control experiments. (a) Control experiment with the ligand-independent transcriptional activator Gal4-VP16.[1] In comparison to 0.1 % DMSO alone, compound **3** at 30 µM had no influence on Gal4-VP16-mediated luciferase reporter expression, thus ruling out unspecific inhibition of the reporter gene in the LXR-Gal4 hybrid assay. Data are the mean ± SEM; N = 3. (b) Control experiment for retinoid X receptor (RXR) modulation. In an RXRα-Gal4 hybrid reporter gene assay, design **3** at 30 µM neither activated RXR nor competed with literature RXR agonist bexarotene (Bexa, 1 µM), thus excluding RXR-mediated inhibition of the LXR/RXR heterodimer. Data are the mean ± SEM in relation to bexarotene at 1 µM; N = 3. (c) Quantification of LXR expression on mRNA level in HEK293T and HepG2 cell lines using qRT-PCR. Expression levels were calculated according to the $2^{-\Delta Ct}$ method relative to GAPDH. The mean Ct values were 20.7 (HepG2), 22.3 (HEK293T) for LXRα and 21.1 (HepG2), 21.4 (HEK293T) for LXRβ. N = 4.

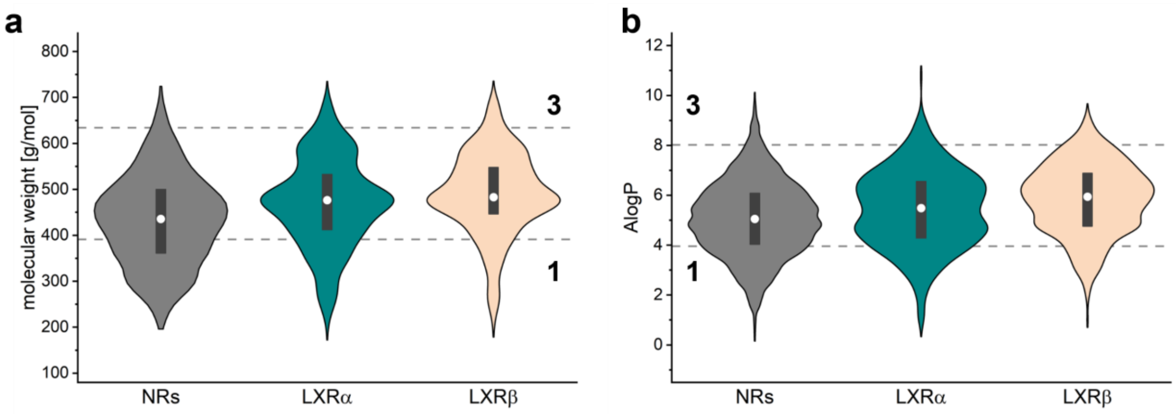

Figure S3. Violin plots of molecular weight (a) and AlogP[2] (b) distribution of known LXR modulators and nuclear receptor ligands from CHEMBL database[3] with annotated cellular bioactivity (pCHEMBL ≥ 4). LXRα (CHEMBL2808) and LXRβ (CHEMBL4093) ligands include more high molecular weight (> 500 g/mol) and highly lipophilic molecules than the average nuclear receptor ligands. The molecular weight and AlogP of the designs **1** and **3** (calculated from https://vcclab.org/web/alogps) are represented by dashed lines. Bars denote the first and third quantile, dots show the median.

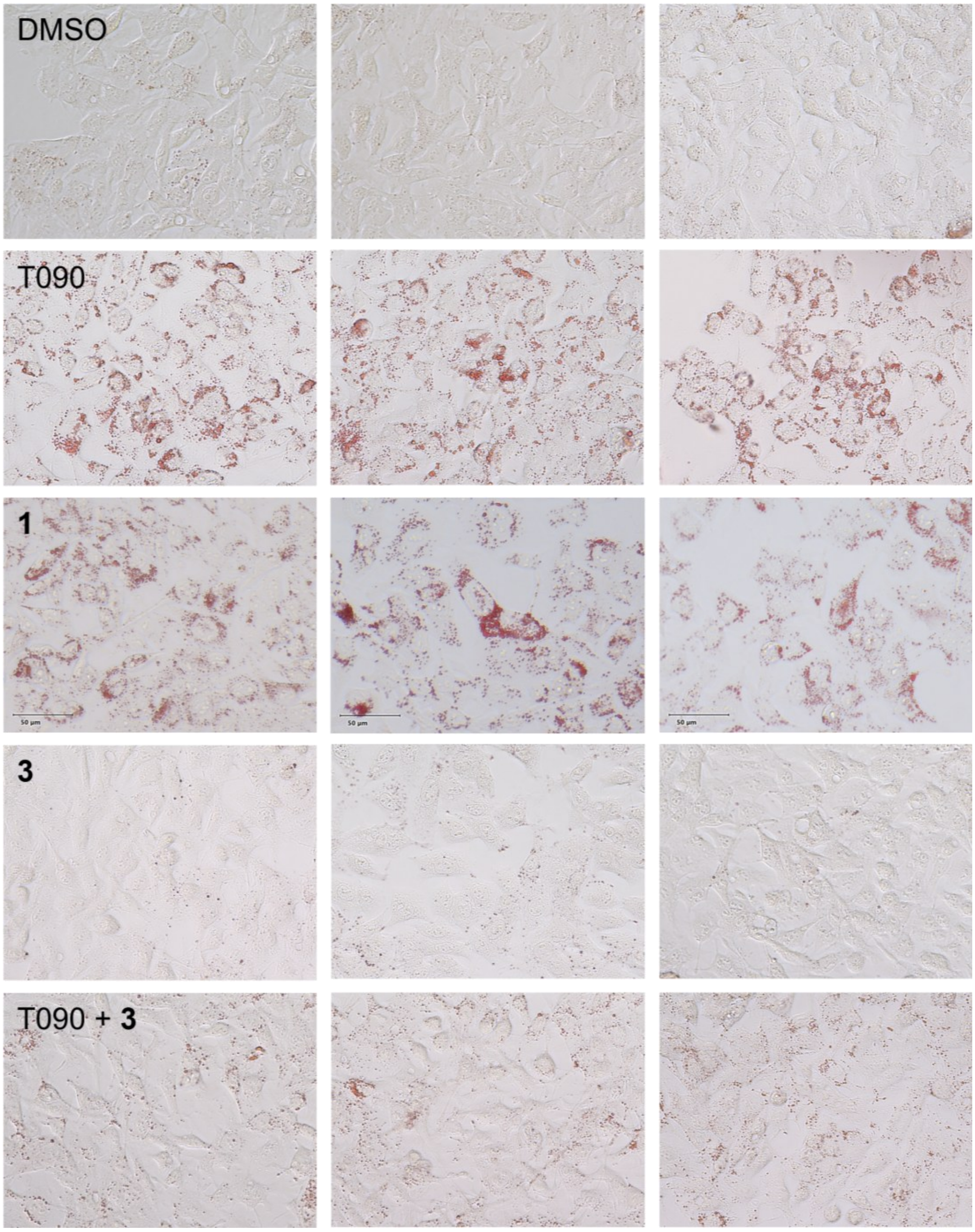

Figure S4. Complete set of images (40x magnification) from lipid staining with Oil Red O in HepG2 cells after 72-hour incubation with the indicated test compounds or 0.1 % DMSO alone: T0901317 (T090, 10 µM), compound **1** (10 µM), compound **3** (30 µM). N = 3.

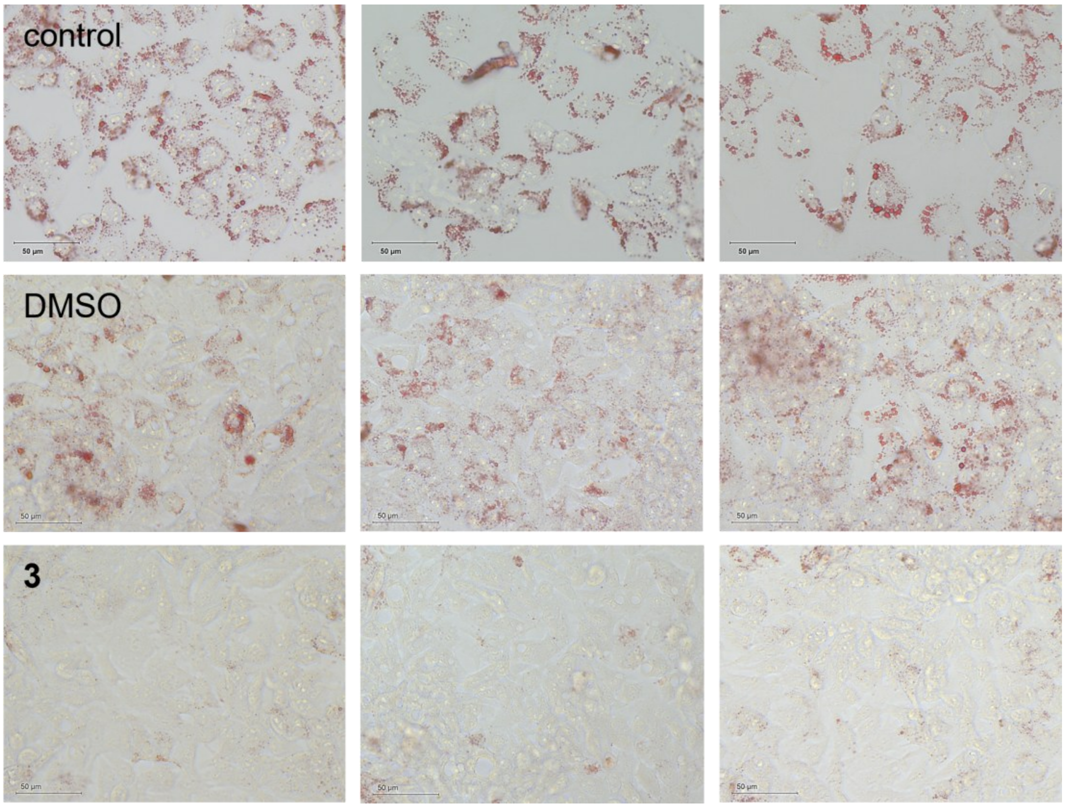

Figure S5. Complete set of images (40x magnification, Oil Red O staining) from the lipolysis assay. HepG2 cells were pre-treated with oleic acid (66.7 μM) and palmitic acid (33.3 μM) to induce lipid accumulation (control). After 24 hours, the lipogenic medium was replaced with 0.1 % DMSO or compound **3** (30 μM) and incubated for another 72 hours. N = 3.

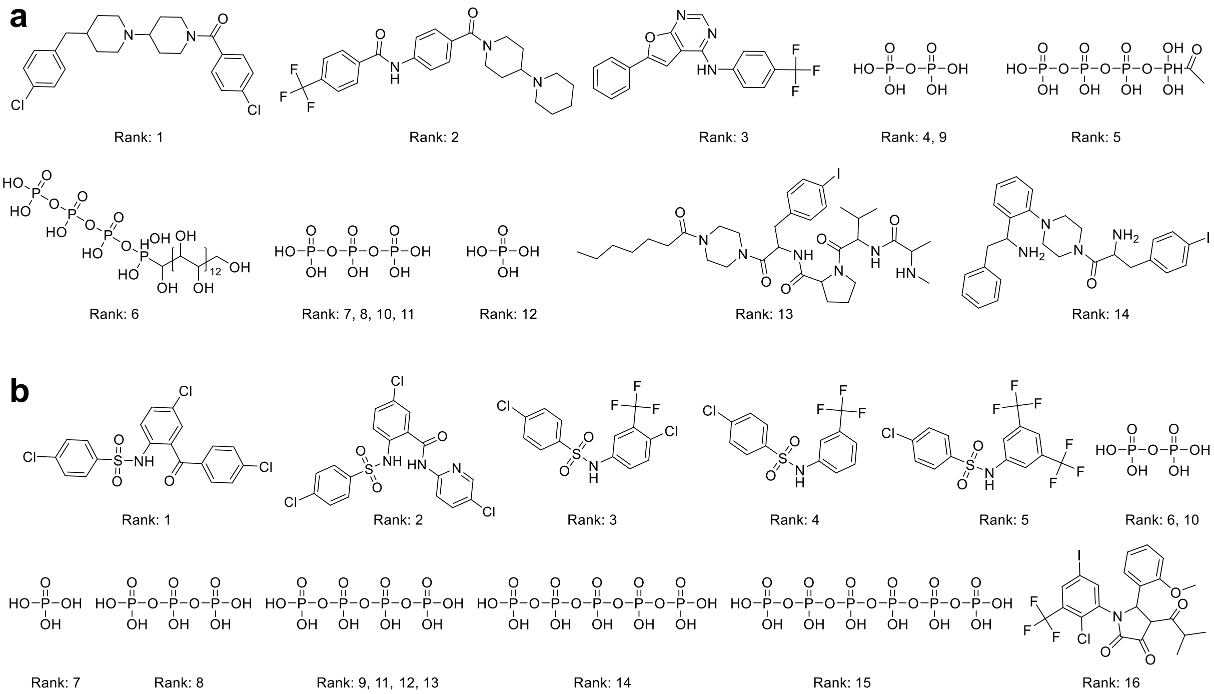

Chart S1. Exemplary beam search results[4] at epochs 8 (a) and 31 (b) from CLM fine-tuning with 12 selected LXR modulators (fine-tuning set I; cf. Table S3) showing the limitations of the initial direct fine-tuning approach with a small training set. Although the fine-tuning molecules lacked phosphorous-containing groups, the designs were enriched in phosphates and pyrophosphates likely representing pre-training artefacts. The small fine-tuning set was apparently insufficient to correct this pre-training bias even after adjusting hyperparameters (batch size, learning rate, number of epochs).

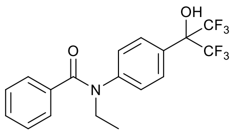

1

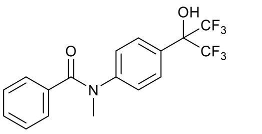

Most similar in first fine-tuning round
(0.68)

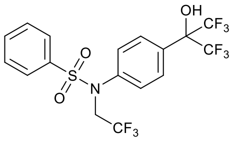

Most similar in template-set
(0.46)

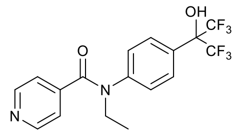

Most similar in ChEMBL pretraining set
(0.76)

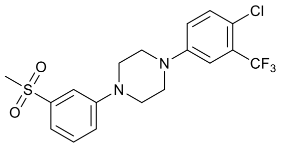

2

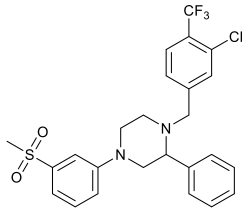

Most similar in first fine-tuning round
(0.53)

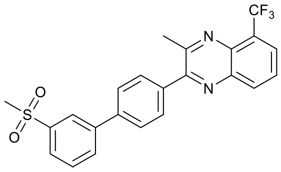

Most similar in template-set
(0.35)

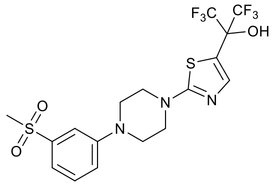

Most similar in ChEMBL pretraining set
(0.49)

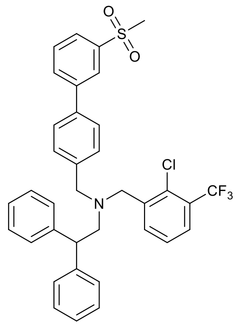

3

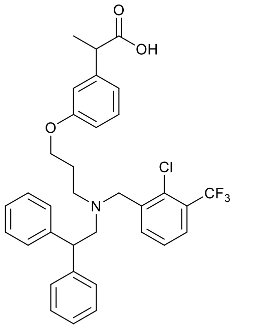

Most similar in first fine-tuning round
(0.51)

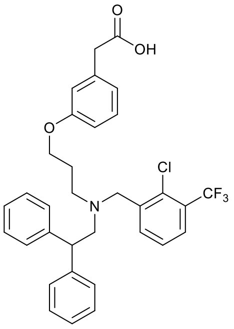

Most similar in template-set
(0.51)

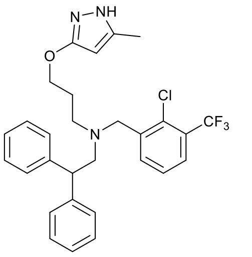

Most similar in ChEMBL pretraining set
(0.51)

Chart S2. Comparison of the selected designs **1**-**3** with the most similar molecules (Tanimoto coefficient) in the fine-tuning set I, the fine-tuning set II and the ChEMBL pretraining set.

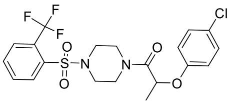

Rank: 1

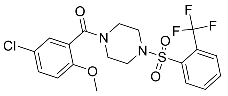

Rank: 2

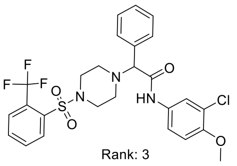

Rank: 3

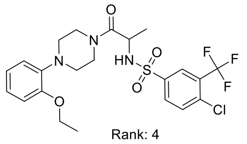

Rank: 4

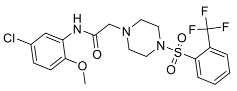

Rank: 5

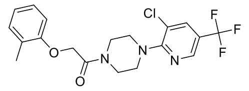

Rank: 6

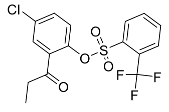

Rank: 7

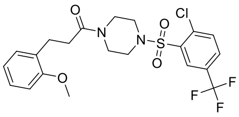

Rank: 8

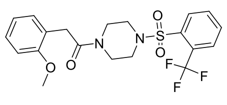

Rank: 9

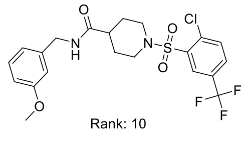

Rank: 10

Chart S3. Results (top 10) of a simulated virtual screening using Tanimoto similarity computed on Morgan fingerprints as ranking criterion and the twelve selected LXR agonists (fine-tuning set I; cf. Table S3) as query molecules. ~ 4 Mio. molecules of the Enamine purchasable compound collection were ranked for similarity to each template and the individual ranks were aggregated by the rank sum. Compounds **1**-**3** were added to the screening set and were not ranked in top 1000 (**1**: rank 132438, **2**: rank 6603, **3**: rank 3403).

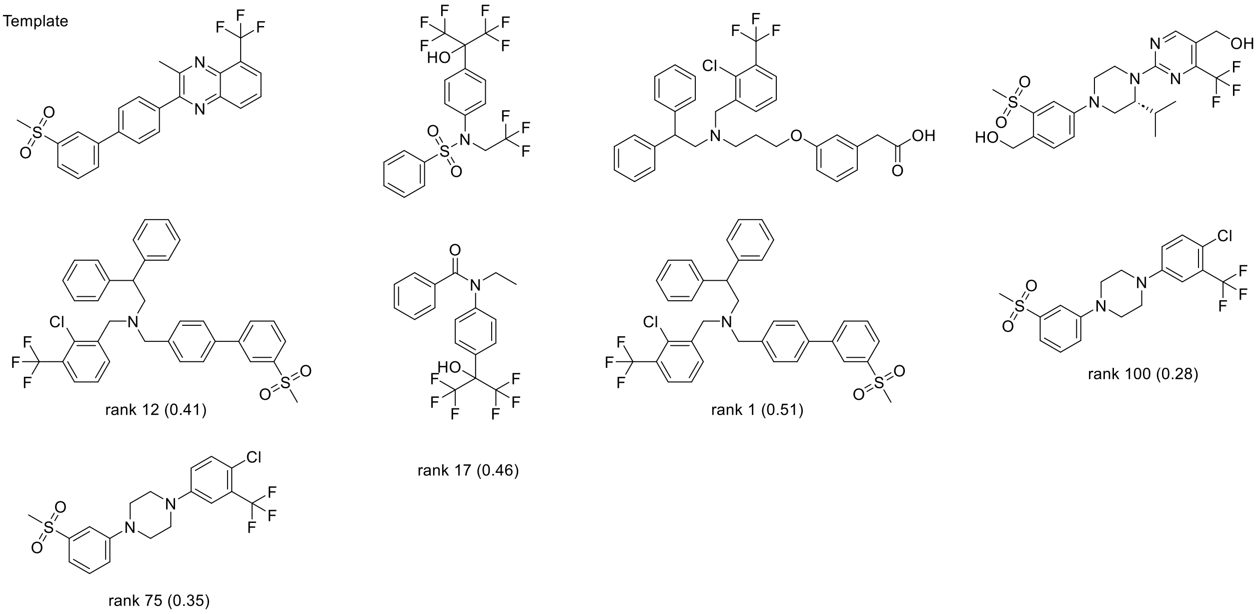

Chart S4. The designs **1-3** would be discoverable (if they were commercially available) by virtual screening but only using the most similar LXR ligands as single query molecules (first row).

2. Supporting Tables

Table S1. Top 200 designs by frequency. The top 30 designs are based on three chemical scaffolds.

Rank	SMILES	Scaffold	Count across selected epochs (5-12)	Max. similarity to fine-tuning set II
1 (Design **1**)	CCN(C(=O)c1ccccc1)c1ccc(C(O)(C(F)(F)F)C(F)(F)F)cc1	1	1788	0.46
2	OC(c1cccc(Cl)c1)(C(F)(F)F)C(F)(F)F	1	1356	0.30
3	OC(c1ccccc1Cl)(C(F)(F)F)C(F)(F)F	1	804	0.28
4	CCN(c1ccc(C(O)(C(F)(F)F)C(F)(F)F)cc1)c1cccc(C(=O)O)c1	1	437	0.40
5	O=C(O)Cc1ccc(OCCCN(Cc2cccc(C(F)(F)F)c2Cl)CC(c2ccccc2)c2ccccc2)cc1	2	333	0.84
6	OC(c1cc(Cl)ccc1Cl)(C(F)(F)F)C(F)(F)F	1	307	0.22
7	O=C(O)Cc1cccc(OCCCN(Cc2ccc(C(F)(F)F)c(Cl)c2)CC(c2ccccc2)c2ccccc2)c1	2	280	0.75
8	O=C(O)Cc1cccc(OCCCN(Cc2ccc(Cl)c(C(F)(F)F)c2)CC(c2ccccc2)c2ccccc2)c1	2	177	0.75
9	CCN(c1ccc(C(O)(C(F)(F)F)C(F)(F)F)cc1)c1cccc(C(N)=O)c1	1	176	0.37
10	O=C(O)Cc1ccc(OCCCN(Cc2ccc(C(F)(F)F)c(Cl)c2)CC(c2ccccc2)c2ccccc2)cc1	2	169	0.63
11	CCN(c1ccc(C(O)(C(F)(F)F)C(F)(F)F)cc1)c1ccc(C(O)(C(F)(F)F)C(F)(F)F)cc1	1	169	0.43
12	CCN(Cc1ccccc1)c1ccc(C(O)(C(F)(F)F)C(F)(F)F)cc1	1	163	0.45
13	O=C(O)Cc1cccc(OCCCN(Cc2ccccc2)Cc2ccc(C(F)(F)F)c(Cl)c2)c1	2	160	0.63
14	O=C(O)Cc1cccc(OCCCN(Cc2ccc(C(F)(F)F)cc2)CC(c2ccccc2)c2ccccc2)c1	2	155	0.70
15	OC(c1cc(Cl)cc(Cl)c1Cl)(C(F)(F)F)C(F)(F)F	1	150	0.22
16	O=C(O)Cc1cccc(OCCCN(Cc2ccc(C(F)(F)F)cc2)Cc2ccc(C(F)(F)F)c(Cl)c2)c1	2	127	0.58
17	CCN(CC(c1ccccc1)c1ccccc1)c1ccc(C(O)(C(F)(F)F)C(F)(F)F)cc1	1	126	0.43
18	CCN(c1ccc(C(O)(C(F)(F)F)C(F)(F)F)cc1)C(c1ccccc1)c1ccccc1	1	119	0.44
19	O=C(O)Cc1cccc(OCCCN(Cc2ccc(C(F)(F)F)c(Cl)c2)Cc2ccc(Cl)c(C(F)(F)F)c2)c1	2	103	0.59
20	O=C(O)Cc1cccc(OCCCN(Cc2cccc(C(F)(F)F)c2)CC(c2ccccc2)c2ccccc2)c1	2	101	0.70
21	O=C(O)Cc1ccc(OCCCN(Cc2ccc(C(F)(F)F)cc2)CC(c2ccccc2)c2ccccc2)cc1	2	94	0.57
22	CCN(C(=O)C1CCCCC1)c1ccc(C(O)(C(F)(F)F)C(F)(F)F)cc1	1	83	0.36

23	NC(=O)Cc1cccc(OCCCN(Cc2ccc(C(F)(F)F)cc2)CC(c2ccccc2)c2ccccc2)c1	2	75	0.61
24	O=C(O)Cc1cccc(OCCCN(Cc2cccc(C(F)(F)F)c2Cl)Cc2cccc(C(F)(F)F)c2Cl)c1	2	74	0.84
25 (Design **2**)	CS(=O)(=O)c1cccc(N2CCN(c3ccc(Cl)c(C(F)(F)F)c3)CC2)c1	3	71	0.35
26	O=C(O)Cc1ccc(OCCCN(Cc2ccc(Cl)c(C(F)(F)F)c2)CC(c2ccccc2)c2ccccc2)cc1	1	66	0.63
27	CCN(c1ccc(C(O)(C(F)(F)F)C(F)(F)F)cc1)c1cccc(C(=O)N(C)C)c1	2	63	0.36
28	O=C(O)Cc1cccc(OCCCN(Cc2ccc(C(F)(F)F)cc2)Cc2cccc(C(F)(F)F)c2Cl)c1	1	61	0.75
29	CCN(C(=O)c1ccccc1)c1ccc(C(O)(C(F)(F)F)C(F)(F)F)c(Cl)c1	2	56	0.32
30	COC(=O)CN(C(=O)c1ccccc1)c1ccc(C(O)(C(F)(F)F)C(F)(F)F)cc1	1	55	0.42
31	O=C(O)Cc1cccc(OCCCN(Cc2ccc(C(F)(F)F)cc2)Cc2ccc(C(F)(F)F)cc2)c1		51	0.57
32	O=C(O)Cc1ccc(OCCCN(Cc2cccc(C(F)(F)F)c2)CC(c2ccccc2)c2ccccc2)cc1		50	0.60
33	NC(=O)Cc1cccc(OCCCN(Cc2ccc(C(F)(F)F)c(Cl)c2)CC(c2ccccc2)c2ccccc2)c1		50	0.66
34	CCN(c1ccc(C(O)(C(F)(F)F)C(F)(F)F)cc1)c1cccc(S(C)(=O)=O)c1		48	0.47
35	O=C(O)Cc1cccc(OCCCN(Cc2ccc(C(F)(F)F)cc2)Cc2ccc(Cl)c(C(F)(F)F)c2)c1		48	0.58
36	CCN(C(=O)c1ccc(F)cc1)c1ccc(C(O)(C(F)(F)F)C(F)(F)F)cc1		47	0.40
37	O=C1CCCc2cc(OCCCN(Cc3ccc(C(F)(F)F)c(Cl)c3)CC(c3ccccc3)c3ccccc3)ccc21		42	0.47
38	CCN(C(=O)C(C)C)c1ccc(C(O)(C(F)(F)F)C(F)(F)F)cc1		37	0.39
39	O=C(O)Cc1cccc(OCCCN(Cc2ccc(Cl)c(Cl)c2Cl)CC(c2ccccc2)c2ccccc2)c1		37	0.75
40	CC(C)(C)c1ccc(CN(CCCOc2cccc(CC(=O)O)c2)CC(c2ccccc2)c2ccccc2)cc1		36	0.63
41	O=C(O)Cc1cccc(OCCCN(Cc2ccccc2)Cc2ccc(Cl)c(C(F)(F)F)c2)c1		35	0.63
42	O=C(O)Cc1cccc(OCCCN(Cc2ccc(C(F)(F)F)c(Cl)c2)Cc2cccc(C(F)(F)F)c2Cl)c1		31	0.74
43	NC(=O)Cc1cccc(OCCCN(Cc2ccc(C(F)(F)F)cc2)Cc2ccc(C(F)(F)F)c(Cl)c2)c1		29	0.49
44	O=C(O)Cc1cccc(OCCCN(Cc2ccccc2)Cc2cccc(C(F)(F)F)c2Cl)c1		29	0.83
45	COC(=O)CN(C(=O)C1CCCCC1)c1ccc(C(O)(C(F)(F)F)C(F)(F)F)cc1		29	0.33
46	O=C(O)Cc1ccc(OCCCN(Cc2ccc(C(F)(F)F)cc2)Cc2ccc(C(F)(F)F)c(Cl)c2)cc1		26	0.46
47	CN(C)C(=O)c1ccc(N2CCN(c3ccc(Cl)c(C(F)(F)F)c3)CC2)cc1		26	0.41
48	FC(F)(F)c1cccc(CN(CCCOc2ccc3c(c2)CCCC3)CC(c2ccccc2)c2ccccc2)c1Cl		26	0.66
49	CC(C)(C)c1cc(N2CCN(c3cccc(S(C)(=O)=O)c3)CC2)cc(C(F)(F)F)c1Cl		26	0.34
50	CC(C)C1CN(c2cccc(S(C)(=O)=O)c2)CCN1c1ccc(Cl)c(C(F)(F)F)c1		25	0.43
51	O=C(O)Cc1cccc(OCCCN(Cc2ccc(Cl)c(C(F)(F)F)c2)Cc2ccc(Cl)c(C(F)(F)F)c2)c1		25	0.62
52	NC(=O)Cc1cccc(OCCCN(Cc2ccc(Cl)c(C(F)(F)F)c2)CC(c2ccccc2)c2ccccc2)c1		23	0.66
53	CS(=O)(=O)c1cccc(N2CCC(C(=O)N3CCN(c4ccc(Cl)c(C(F)(F)F)c4)CC3)CC2)c1		22	0.33
54	O=C(O)Cc1cccc(OCCCN(Cc2ccccc2)Cc2ccc(C(F)(F)F)cc2)c1		22	0.58
55	CCN(Cc1cccc(C(F)(F)F)c1Cl)c1ccc(C(O)(C(F)(F)F)C(F)(F)F)cc1		22	0.34
56	CCN(c1ccc(C(O)(C(F)(F)F)C(F)(F)F)cc1)c1cccc(C(F)(F)F)c1Cl		22	0.36
57	O=C(O)Cc1cccc(OCCCN(Cc2ccc(C(F)(F)F)c(Cl)c2Cl)CC(c2ccccc2)c2ccccc2)c1		21	0.89
58	CC1CCc2cc(OCCCN(Cc3ccc(C(F)(F)F)c(Cl)c3)CC(c3ccccc3)c3ccccc3)ccc21		20	0.47
59	CCN(c1ccc(C(O)(C(F)(F)F)C(F)(F)F)cc1)C(C(=O)N(C)C)c1ccccc1		19	0.41
60	O=C(O)Cc1ccc(OCCCN(Cc2ccc(Cl)c(Cl)c2Cl)CC(c2ccccc2)c2ccccc2)cc1		19	0.60
61	O=C(O)Cc1ccc(OCCCN(CCCOc2ccc3c(c2)CCCC3)Cc2ccc(C(F)(F)F)c(Cl)c2)cc1		18	0.45
62	CN(c1ccc(C(O)(C(F)(F)F)C(F)(F)F)cc1)S(=O)(=O)c1ccc(F)cc1		18	0.53
63	CCN(CC(=O)OC)c1ccc(C(O)(C(F)(F)F)C(F)(F)F)cc1		18	0.38
64	CN(C)C(=O)C1CCCc2cc(OCCCN(Cc3ccc(C(F)(F)F)c(Cl)c3)c3ccccc3)ccc21		18	0.34
65	NC(=O)Cc1cccc(OCCCN(Cc2ccc(C(F)(F)F)c(Cl)c2)Cc2cccc(C(F)(F)F)c2Cl)c1		17	0.63
66	FC(F)(F)c1ccc(CN(CCCOc2ccc3c(c2)CCCC3)CC(c2ccccc2)c2ccccc2)cc1		17	0.45
67	O=C1CCCc2cc(OCCCN(Cc3cccc(C(F)(F)F)c3)CC(c3ccccc3)c3ccccc3)ccc21		17	0.45
68	O=C(O)C1CCCc2cc(OCCCN(Cc3ccc(C(F)(F)F)c(Cl)c3)CC(c3ccccc3)c3ccccc3)ccc21		16	0.49

69	CN(C)C(=O)N(CCCOc1ccc2c(c1)CCCC2)Cc1ccc(C(F)(F)F)cc1	16	0.29
70	O=C1CCCc2cc(OCCCN(Cc3ccc(Cl)c(C(F)(F)F)c3)CC(c3ccccc3)c3ccccc3)ccc21	16	0.47
71	NC(=O)Cc1cccc(OCCCN(Cc2ccc(Cl)c(Cl)c2Cl)CC(c2ccccc2)c2ccccc2)c1	16	0.65
72	O=C(O)C1CCc2cc(OCCCN(Cc3cccc(C(F)(F)F)c3Cl)c3ccccc3)ccc2C1	15	0.46
73	OC(c1ccc(N2CCN(c3ccc(Cl)c(C(F)(F)F)c3)CC2)cc1)(C(F)(F)F)C(F)(F)F	15	0.31
74	O=C(O)Cc1ccc(OCCCN(Cc2ccc(C(F)(F)F)c(Cl)c2Cl)CC(c2ccccc2)c2ccccc2)cc1	15	0.72
75	O=C(O)Cc1cccc(OCCCN(Cc2ccc(Cl)c(C(F)(F)F)c2)Cc2cccc(C(F)(F)F)c2Cl)c1	15	0.74
76	O=C1CCc2cc(OCCCN(Cc3cccc(C(F)(F)F)c3Cl)CC(c3ccccc3)c3ccccc3)ccc2N1	15	0.62
77	COC(=O)CN(Cc1ccccc1)c1ccc(C(O)(C(F)(F)F)C(F)(F)F)cc1	14	0.42
78	CC(C)(C)c1cccc(CN(CCCOc2cccc(CC(=O)O)c2)CC(c2ccccc2)c2ccccc2)c1	14	0.63
79	CS(=O)(=O)c1cccc(N2CCN(c3ccc(C(F)(F)F)c(Cl)c3)CC2)c1	14	0.35
80	O=C(O)Cc1cccc(OCCCN(CCCOc2ccc3c(c2)CCCC3)Cc2ccc(C(F)(F)F)c(Cl)c2)c1	14	0.54
81	CC(C)(C)c1ccc(CN(CCCOc2ccc(CC(=O)O)cc2)CC(c2ccccc2)c2ccccc2)cc1	14	0.50
82	O=C(O)Cc1cccc(OCCCN(Cc2cccc(C(F)(F)F)c2Cl)Cc2ccc(Cl)c(Cl)c2Cl)c1	14	0.77
83	FC(F)(F)c1cccc(CN(CCCOc2ccc3c(c2)CCCC3)c2ccccc2)c1Cl	13	0.47
84	CC(C)C1CN(c2ccc(Cl)c(C(F)(F)F)c2)CCN1c1nccc(C(F)(F)F)n1	13	0.45
85	NC(=O)Cc1cccc(OCCCN(Cc2cccc(C(F)(F)F)c2Cl)Cc2cccc(C(F)(F)F)c2Cl)c1	13	0.71
86	O=C(O)Cc1cccc2c(OCCCN(Cc3cccc(C(F)(F)F)c3Cl)CC(c3ccccc3)c3ccccc3)cccc12	13	0.70
87	CS(=O)(=O)c1cccc(-c2ccc(CN(Cc3ccc(C(F)(F)F)c(Cl)c3)CC(c3ccccc3)c3ccccc3)cc2)c1	12	0.40
88	CC(C)CN(c1ccc(C(O)(C(F)(F)F)C(F)(F)F)cc1)S(=O)(=O)c1ccc(F)cc1	12	0.59
89	O=C1CCCc2cc(OCCCN(Cc3cccc(C(F)(F)F)c3Cl)CC(c3ccccc3)c3ccccc3)ccc21	12	0.63
90	NC(=O)Cc1cccc(OCCCN(CCCOc2ccc(CC(=O)O)cc2)Cc2cccc(C(F)(F)F)c2Cl)c1	12	0.73
91	NC(=O)Cc1cccc(OCCCN(CCCOc2cccc(CC(=O)O)c2)Cc2ccc(C(F)(F)F)cc2)c1	12	0.53
92	CCN(Cc1ccc(C(F)(F)F)c(Cl)c1)c1ccc(C(O)(C(F)(F)F)C(F)(F)F)cc1	12	0.32
93	CC(C)(C)c1cccc(CN(CCCOc2ccc(CC(=O)O)cc2)CC(c2ccccc2)c2ccccc2)c1	11	0.54
94	OCc1c(C(F)(F)F)nc(N2CCN(c3ccc(Cl)c(C(F)(F)F)c3)CC2)nc1C(F)(F)F	11	0.34
95	CCN(CC(=O)N(C)C)c1ccc(C(O)(C(F)(F)F)C(F)(F)F)cc1	11	0.38
96	CS(=O)(=O)c1cccc(N2CCN(c3ccc(Cl)c(C(F)(F)F)c3)C(c3ccccc3)C2)c1	11	0.31
97	O=C(O)Cc1cccc(OCCCN(Cc2ccc(C(F)(F)F)c(Cl)c2)Cc2ccc(C(F)(F)F)c(Cl)c2)c1	11	0.62
98	O=C(O)Cc1cccc(OCCCN(Cc2ccc(C(F)(F)F)cc2)Cc2cccc(C(F)(F)F)c2)c1	11	0.54
99	COC(=O)CN(C(=O)C(C)C)c1ccc(C(O)(C(F)(F)F)C(F)(F)F)cc1	10	0.36
100	O=C(O)Cc1ccc(OCCCN(CCCOc2cccc3ccccc23)Cc2cccc(C(F)(F)F)c2Cl)cc1	10	0.61
101	O=C(O)Cc1cccc(OCCCN(CCCOc2cccc3c2CCCC3)Cc2ccc(C(F)(F)F)c(Cl)c2)c1	10	0.51
102	CCN(c1ccc(C(=O)N(C)C)cc1)c1ccc(C(O)(C(F)(F)F)C(F)(F)F)cc1	10	0.38
103	CS(=O)(=O)c1cccc(N2CCN(c3ncc(CO)c(C(F)(F)F)n3)CC2)c1	10	0.55
104	CS(=O)(=O)c1cc(N2CCC(C(=O)N3CCN(c4ccc(Cl)c(C(F)(F)F)c4)CC3)CC2)ccc1CO	10	0.45
105	O=C(O)Cc1ccc(OCCCN(Cc2ccc(C(F)(F)F)cc2)Cc2ccc(Cl)c(C(F)(F)F)c2)cc1	10	0.46
106	CC(C)C1CN(c2ncc(CO)c(C(F)(F)F)n2)CCN1c1ccc(CO)c(S(C)(=O)=O)c1	10	0.88
107	CS(=O)(=O)c1cccc(N2CCN(c3ccc(C(O)(C(F)(F)F)C(F)(F)F)cc3)CC2)c1	10	0.38
108	CN1c2ccc(OCCCN(Cc3ccc(C(F)(F)F)cc3)CC(c3ccccc3)c3ccccc3)cc2CCC1C(=O)O	10	0.42
109	CN1c2ccc(OCCCN(Cc3cccc(C(F)(F)F)c3Cl)CC(c3ccccc3)c3ccccc3)cc2CCC1C(=O)O	10	0.62
110	CC(C)CN(C(=O)c1ccccc1)c1ccc(C(O)(C(F)(F)F)C(F)(F)F)cc1	10	0.43
111	O=C(O)Cc1ccc(OCCCN(CCCOc2cccc3c2CCCC3)Cc2ccc(C(F)(F)F)c(Cl)c2)cc1	10	0.43
112	O=C(O)Cc1ccc(N2CCN(c3ccc(Cl)c(C(F)(F)F)c3)CC2)cc1	10	0.30
113	O=S(=O)(c1ccc(F)cc1)N(CC(F)(F)F)c1ccc(C(O)(C(F)(F)F)C(F)(F)F)cc1	10	0.80
114	O=C(O)Cc1cccc(OCCCN(Cc2cccc(C(F)(F)F)c2)Cc2cccc(C(F)(F)F)c2Cl)c1	9	0.75
115	CC(C)C1CN(c2cccc(S(C)(=O)=O)c2)CCN1c1ccc(C(F)(F)F)c(Cl)c1	9	0.43

116	CC(C)CN(C(=O)C(C)C)c1ccc(C(O)(C(F)(F)F)C(F)(F)F)cc1	9	0.38
117	CC(C)(C)c1ccccc1CN(CCCOc1ccc(CC(=O)O)cc1)CC(c1ccccc1)c1ccccc1	9	0.60
118	OC(c1ccc(-c2ccccc2)cc1)(C(F)(F)F)C(F)(F)F	9	0.44
119	O=C(O)Cc1ccc(OCCCN(Cc2ccccc2)Cc2ccc(C(F)(F)F)c(Cl)c2)cc1	9	0.53
120	O=C(O)Cc1ccc(OCCCN(Cc2ccc(C(F)(F)F)cc2)Cc2cccc(C(F)(F)F)c2Cl)cc1	9	0.63
121	CCN(CC(C(C)C)N(CC)c1ccc(C(O)(C(F)(F)F)C(F)(F)F)cc1)c1cccc(S(C)(=O)=O)c1	9	0.40
122	CC(C)C1CN(c2ccc(C(O)(C(F)(F)F)C(F)(F)F)cc2)CCN1S(=O)(=O)c1ccc(F)cc1	9	0.33
123	NC(=O)Cc1cccc(OCCCN(CCCOc2cccc(CC(=O)O)c2)Cc2cccc(C(F)(F)F)c2Cl)c1	9	0.78
124	NC(=O)Cc1cccc(OCCCN(Cc2ccccc2)Cc2ccc(C(F)(F)F)c(Cl)c2)c1	9	0.54
125	O=C(O)Cc1cccc(OCCCN(Cc2ccccc2)CC(c2ccccc2)c2ccccc2)c1	9	0.69
126	CC(C)C(=O)N(c1ccc(C(O)(C(F)(F)F)C(F)(F)F)cc1)C(C)C	9	0.41
127	CCN(c1ccc(C(O)(C(F)(F)F)C(F)(F)F)cc1)C(C)(C)c1ccccc1	9	0.48
128	O=C(O)Cc1cccc(OCCCN(Cc2ccccc2)Cc2ccc(Cl)c(Cl)c2Cl)c1	9	0.61
129	CS(=O)(=O)c1cccc(-c2ccc(CN(Cc3cccc(C(F)(F)F)c3Cl)CC(c3ccccc3)c3ccccc3)cc2)c1	8	0.51
130	NC(=O)Cc1ccc(OCCCN(Cc2cccc(C(F)(F)F)c2Cl)CC(c2ccccc2)c2ccccc2)cc1	8	0.73
131	O=C(O)C1CCCc2cc(OCCCN(Cc3ccc(C(F)(F)F)c(Cl)c3)c3ccccc3)ccc21	8	0.36
132	O=C(O)C1CCCc2cc(OCCCN(Cc3ccc(Cl)c(C(F)(F)F)c3)CC(c3ccccc3)c3ccccc3)ccc21	8	0.49
133	CC(C)C1CN(S(=O)(=O)c2ccc(F)cc2)CCN1c1ccc(C(O)(C(F)(F)F)C(F)(F)F)cc1	8	0.31
134	FC(F)(F)c1ccc(CN(CCCOc2ccc3c(c2)CC[CH]3)CC(c2ccccc2)c2ccccc2)cc1Cl	8	0.47
135	OC(c1cc(Cl)c(Cl)c(Cl)c1Cl)(C(F)(F)F)C(F)(F)F	8	0.22
136	CC(C)(C)c1cc(N2CCN(c3ccc(CO)c(S(C)(=O)=O)c3)CC2)cc(C(F)(F)F)c1Cl	8	0.47
137	O=C(O)Cc1cccc(OCCCN(CCCOc2ccc3c(c2)CCCC3)Cc2ccc(C(F)(F)F)cc2)c1	7	0.49
138	O=C(O)Cc1ccc(OCCCN(Cc2ccc(C(F)(F)F)c(Cl)c2)Cc2ccc(Cl)c(C(F)(F)F)c2)cc1	7	0.47
139	NC(=O)Cc1cccc(OCCCN(Cc2ccc(C(F)(F)F)cc2)Cc2ccc(Cl)c(C(F)(F)F)c2)c1	7	0.49
140	NC(=O)Cc1cccc(OCCCN(CCCOc2ccc3c(c2)CCCC3)Cc2ccc(C(F)(F)F)c(Cl)c2)c1	7	0.46
141	O=C(O)Cc1ccc(OCCCN(CCCOc2cccc3ccccc23)Cc2ccc(C(F)(F)F)c(Cl)c2)cc1	7	0.47
142	CN1C(=O)C(c2ccccc2)=C(c2ccccc2)C1=O	7	0.23
143	O=C(O)C1CCc2cc(OCCCN(Cc3ccc(C(F)(F)F)cc3)c3ccccc3)ccc2C1	7	0.31
144	NC(=O)Cc1cccc(OCCCN(Cc2ccc(Cl)c(C(F)(F)F)c2)Cc2cccc(C(F)(F)F)c2Cl)c1	7	0.63
145	CC(C)CC(C(=O)N1CCN(c2cccc(S(C)(=O)=O)c2)CC1)c1ccccc1	7	0.25
146	NC(=O)Cc1cccc(OCCCN(CCCOc2ccc(CC(=O)O)cc2)Cc2ccc(C(F)(F)F)cc2)c1	7	0.51
147	FC(F)(F)c1ccc(CN(CCCOc2ccc3c(c2)CC[CH]3)CC(c2ccccc2)c2ccccc2)cc1	7	0.43
148	CCN(c1ccc(C(O)(C(F)(F)F)C(F)(F)F)cc1)C(c1ccccc1)C(C)C	7	0.42
149	OC(c1ccccc1)(C(F)(F)F)C(F)(F)F	7	0.45
150	O=C(O)Cc1cccc(OCCCN(CCCOc2cccc3ccccc23)Cc2cccc(C(F)(F)F)c2Cl)c1	7	0.74
151	CC(C)(C)c1ccccc1CN(CCCOc1cccc(CC(=O)O)c1)CC(c1ccccc1)c1ccccc1	7	0.75
152	OC(c1ccc(Cl)c(Cl)c1)(C(F)(F)F)C(F)(F)F	7	0.30
153	O=C(c1ccccc1)N(CC(F)(F)F)c1ccc(C(O)(C(F)(F)F)C(F)(F)F)cc1	7	0.55
154	CS(=O)(=O)c1cccc(N2CCN(c3ccc(C(F)(F)F)cc3)CC2)c1	6	0.33
155	O=C(O)Cc1cccc(OCCCN(CCCOc2cccc3c2CCCC3)Cc2ccc(C(F)(F)F)cc2)c1	6	0.47
156	CS(=O)(=O)c1cccc(-c2ccc(-c3ccc(C(F)(F)F)cc3)cc2)c1	6	0.54
157	O=C(O)Cc1cccc(OCCCN(CCCOc2cccc3c2CCCC3)Cc2cccc(C(F)(F)F)c2Cl)c1	6	0.67
158	FC(F)(F)c1ccc(CN(CCCOc2ccc3c(c2)CCCC3)CCCOc2ccc3c(c2)CCCC3)cc1	6	0.31
159	CC(C)(C)C(=O)N1CCN(c2ccc(C(O)(C(F)(F)F)C(F)(F)F)cc2Cl)CC1	6	0.35
160	O=C(O)Cc1ccc(OCCCN(Cc2ccccc2)CC(c2ccccc2)c2ccccc2)cc1	6	0.56
161	O=C1CCc2cc(OCCCN(Cc3ccc(C(F)(F)F)cc3)CC(c3ccccc3)c3ccccc3)ccc2N1	6	0.43
162	CC(C)C1CCCN(c2ccc(C(O)(C(F)(F)F)C(F)(F)F)cc2)C1	6	0.28

163	CC(C)C1CN(c2cccc(S(C)(=O)=O)c2)CCN1c1ncc(CO)c(C(F)(F)F)n1	6	0.78
164	CS(=O)(=O)c1cccc(N2CCN(c3nccc(C(F)(F)F)n3)CC2)c1	6	0.34
165	OCc1cnc(N2CCN(c3ccc(Cl)c(C(F)(F)F)c3)CC2)nc1C(F)(F)F	6	0.47
166	O=S(=O)(c1ccc(F)cc1)N1CCN(c2ccc(C(O)(C(F)(F)F)C(F)(F)F)cc2)CC1	6	0.36
167	O=C(O)C1CCc2cc(OCCCN(Cc3cccc(C(F)(F)F)c3Cl)c3ccccc3)ccc2N1Cc1ccccc1	6	0.47
168	CS(=O)(=O)c1cccc(-c2ccc(-c3ccc(C(O)(C(F)(F)F)C(F)(F)F)cc3)cc2)c1	6	0.47
169	O=C(O)Cc1cccc(OCCCN(Cc2ccc(C(F)(F)F)cc2)C(=O)c2cccc(Cl)c2Cl)c1	6	0.50
170	O=C(O)Cc1ccc(OCCCN(Cc2ccccc2)Cc2cccc(C(F)(F)F)c2Cl)cc1	6	0.70
171	O=C(O)Cc1cccc(OCCCN(CCCOc2cccc(CC(=O)O)c2)Cc2ccc(C(F)(F)F)cc2)c1	6	0.57
172	Clc1ccc(CN(CCCOc2ccc3c(c2)CCCC3)CC(c2ccccc2)c2ccccc2)c(Cl)c1Cl	6	0.46
173	O=C(O)C1CCCc2cc(OCCCN(Cc3cccc(C(F)(F)F)c3)CC(c3ccccc3)c3ccccc3)ccc21	6	0.46
174	O=C1CCCc2cc(OCCCN(Cc3ccc(C(F)(F)F)cc3)CC(c3ccccc3)c3ccccc3)ccc21	6	0.43
175	CC(C)(C)c1cc(N2CCN(c3ccc(CC(=O)O)cc3)CC2)cc(C(F)(F)F)c1Cl	6	0.30
176	O=C(O)Cc1cccc(OCCCN(CCCOc2cccc3ccccc23)Cc2ccc(C(F)(F)F)c(Cl)c2)c1	6	0.55
177	OC(c1ccc(N(Cc2ccccc2)CC(c2ccccc2)c2ccccc2)cc1)(C(F)(F)F)C(F)(F)F	6	0.42
178	O=C(c1ccccc1)N1CCN(c2ccc(Cl)c(C(F)(F)F)c2)C(c2ccccc2)C1	6	0.25
179	FC(F)(F)c1cc(CN(CCCOc2ccc3c(c2)CC[CH]3)CC(c2ccccc2)c2ccccc2)ccc1Cl	6	0.47
180	O=C(O)Cc1ccc(OCCCN(CCCOc2cccc(CC(=O)O)c2)Cc2ccc(C(F)(F)F)c(Cl)c2)cc1	6	0.59
181	O=C(O)Cc1cccc(OCCCN(Cc2ccccc2)Cc2ccc(C(F)(F)F)c(Cl)c2Cl)c1	6	0.73
182	CCN(c1cccc(C(=O)O)c1)c1ccc(C(O)(C(F)(F)F)C(F)(F)F)c(Cl)c1	6	0.29
183	CS(=O)(=O)c1cccc(N2CCCN(c3ccc(C(O)(C(F)(F)F)C(F)(F)F)cc3)CC2)c1	5	0.36
184	CS(=O)(=O)c1cccc(N2CCC(C(=O)N3CCN(c4cccc(C(F)(F)F)c4)CC3)CC2)c1	5	0.29
185	O=C(O)Cc1ccc(OCCCN(Cc2ccc(C(F)(F)F)c(Cl)c2)Cc2cccc(C(F)(F)F)c2Cl)cc1	5	0.64
186	NC(=O)Cc1cccc(OCCCN(Cc2ccccc2)Cc2cccc(C(F)(F)F)c2Cl)c1	5	0.72
187	CN(C)C(=O)CN(C(=O)c1ccccc1)c1ccc(C(O)(C(F)(F)F)C(F)(F)F)cc1	5	0.43
188	CC(C)(C)c1ccc(CN(CCCOc2ccc(CC(=O)O)cc2)CC(c2ccccc2)c2ccccc2)c(Cl)c1Cl	5	0.60
189	CC1CCc2cc(OCCCN(Cc3ccc(C(F)(F)F)cc3)CC(c3ccccc3)c3ccccc3)ccc21	5	0.43
190	CC(C)(C)c1ccc(CN(CCCOc2cccc(CC(N)=O)c2)CC(c2ccccc2)c2ccccc2)cc1	5	0.54
191	CC(C)C1CN(Cc2cccc(S(C)(=O)=O)c2)CCN1c1cccc(C(F)(F)F)c1	5	0.35
192	CC(C)C1CN(c2cccc(S(C)(=O)=O)c2)CCN1c1nccc(C(F)(F)F)n1	5	0.52
193	FC(F)(F)c1ccc(-c2nc(-c3ccccc3)n[nH]2)cc1	5	0.21
194	NC(=O)Cc1cccc(OCCCN(CCCOc2cccc(CC(N)=O)c2)Cc2ccc(C(F)(F)F)cc2)c1	5	0.47
195	CN(C)C(=O)C1(c2ccccc2)CCN(c2cccc(S(C)(=O)=O)c2)CC1	5	0.23
196	FC(F)(F)c1cc(N2CCN(c3ncc(Cl)c(C(F)(F)F)n3)CC2)ccc1Cl	5	0.33
197	O=C(O)Cc1ccc(OCCCN(Cc2ccc(C(F)(F)F)c(Cl)c2)Cc2ccc(C(F)(F)F)c(Cl)c2)cc1	5	0.49
198	CCN(c1ccccc1)c1ccc(C(O)(C(F)(F)F)C(F)(F)F)cc1	5	0.49
199	O=C(O)C1CCCc2cc(OCCCN(Cc3cccc(C(F)(F)F)c3Cl)CC(c3ccccc3)c3ccccc3)ccc21	5	0.64
200	CC(C)C1CN(c2cccc(C(F)(F)F)c2)CCN1c1ncc(Cl)c(C(F)(F)F)n1	5	0.53

Table S2. Top 200 designs based on docking. The co-crystallized ligands were redocked in 5 repeats for validation.

Rank	SMILES	Count across selected epochs (5-12)	Max. similarity to fine-tuning set II	Dock	Dock 5jy3[5]	Dock 3ipq[6]
BMS-852927	OC(C)(C1=CN(C(C(C2=C(C=CC=C2Cl)Cl)(C)C)=N1)C3=CC=C(C=C3F)C4=CC(S(=O)(C)=O)=C(C(F)=C4)CO)C				-15.1 ± 0.0<br>RMSD 1.528 ± 0.004	-
GW3965	O=C(O)CC1=CC=CC(OCCCN(CC2=CC=CC(C(F)(F)F)=C2Cl)CC(C3=CC=CC=C3)C4=CC=CC=C4)=C1				-	-14.0 ± 0.0<br>RMSD 0.706 ± 0.057
1	CS(=O)(=O)c1cccc(-c2ccc(CN(Cc3ccc(F)c(C(F)(F)F)c3Cl)CC(c3ccccc3)c3ccccc3)cc2)c1	1	0.38	14.44	-13.69	-15.23
2	CS(=O)(=O)c1cc(-c2ccc(CN(Cc3cccc(C(F)(F)F)c3Cl)CC(c3ccccc3)c3ccccc3)cc2)ccc1Cl	1	0.50	14.44	-13.93	-14.97
3<br>**(Design 3)**	CS(=O)(=O)c1cccc(-c2ccc(CN(Cc3cccc(C(F)(F)F)c3Cl)CC(c3ccccc3)c3ccccc3)cc2)c1	8	0.51	14.37	-13.03	-15.84
4	CS(=O)(=O)c1cccc(-c2ccc(CN(Cc3ccc(C(F)(F)F)cc3)CC(c3ccccc3)c3ccccc3)cc2)c1	1	0.39	14.31	-13.23	-15.47

5	CS(=O)(=O)c1cc(-c2ccc(CN(Cc3cccc(C(F)(F)F)c3Cl)CC(c3ccccc3)c3ccccc3)cc2)ccc1CO	1	0.50	14.15	-13.22	-15.15
6	CS(=O)(=O)c1cccc(-c2ccc(C(=O)N(Cc3ccc(Cl)c(C(F)(F)F)c3)CC(c3ccccc3)c3ccccc3)cc2)c1	1	0.37	13.93	-13.46	-14.42
7	CN(C)C(=O)c1cccc(-c2ccc(CN(Cc3ccc(C(F)(F)F)c(Cl)c3)CC(c3ccccc3)c3ccccc3)cc2)c1	1	0.40	13.80	-13.04	-14.61
8	OC(c1ccc(N(Cc2ccc(F)c(C(F)(F)F)c2)CC(c2ccccc2)c2ccccc2)cc1)(C(F)(F)F)C(F)(F)F	2	0.38	13.80	-12.99	-14.67
9	O=C1CCc2cc(OCCCN(Cc3cccc(C(F)(F)F)c3)CC(c3ccccc3)c3ccccc3)ccc2N1	3	0.44	13.78	-13.10	-14.50
10	O=C1CCCc2cc(OCCCN(Cc3ccc(C(F)(F)F)c(Cl)c3Cl)CC(c3ccccc3)c3ccccc3)ccc21	2	0.55	13.74	-12.75	-14.82
11	CN1C(=O)CCc2cc(OCCCN(Cc3cccc(C(F)(F)F)c3Cl)CC(c3ccccc3)c3ccccc3)ccc2C1=O	1	0.59	13.74	-12.71	-14.86
12	FC(F)(F)c1ccc(CN(CCCOc2cccc3ccccc23)CC(c2ccccc2)c2ccccc2)cc1Cl	1	0.47	13.71	-12.77	-14.72
13	FC(F)(F)c1cccc(CN(CCCOc2cccc3ccccc23)CC(c2ccccc2)c2ccccc2)c1Cl	1	0.61	13.70	-12.59	-14.90
14	FC(F)(F)c1cccc(CN(Cc2ccc(-c3ccccc3)cc2)CC(c2ccccc2)c2ccccc2)c1Cl	1	0.56	13.66	-12.50	-14.93
15	FC(F)(F)c1cccc(CN(CCCOc2ccc3c(c2)CCCC3)CC(c2ccccc2)c2ccccc2)c1	3	0.47	13.63	-13.24	-14.03
16	CS(=O)(=O)c1cccc(-c2ccc(-c3ccc(CN(Cc4ccc(C(F)(F)F)c(Cl)c4)CC(c4ccccc4)c4ccccc4)cc3)cc2)c1	1	0.40	13.63	-13.75	-13.51
17	O=C(O)Cc1cccc(OCCCC(CC(c2ccccc2)c2ccccc2)c2ccc(Cl)c(C(F)(F)F)c2)c1	1	0.56	13.62	-13.08	-14.17
18	O=C1CCc2c(cccc2OCCCN(Cc2cccc(C(F)(F)F)c2Cl)CC(c2ccccc2)c2ccccc2)N1	1	0.55	13.60	-12.40	-14.92
19	O=C(O)Cc1cccc2c(OCCCN(Cc3cccc(C(F)(F)F)c3Cl)CC(c3ccccc3)c3ccccc3)cccc12	13	0.70	13.60	-12.98	-14.24
20	FC(F)(F)c1cc(CN(CCCOc2cccc3ccccc23)CC(c2ccccc2)c2ccccc2)ccc1Cl	1	0.47	13.58	-12.75	-14.48
21	O=C(O)C1CCc2cc(OCCCN(Cc3ccc(Cl)c(C(F)(F)F)c3)CC(c3ccccc3)c3ccccc3)ccc2C1	1	0.48	13.58	-12.51	-14.73
22	O=C1CCCc2cc(OCCCN(Cc3ccc(C(F)(F)F)c(Cl)c3)CC(c3ccccc3)c3ccccc3)ccc21	42	0.47	13.55	-12.49	-14.70
23	O=C1CCCc2c(OCCCN(Cc3cccc(C(F)(F)F)c3)CC(c3ccccc3)c3ccccc3)cccc21	1	0.40	13.55	-12.63	-14.54
24	OC(c1ccc(N(Cc2ccc(C(F)(F)F)c(Cl)c2)CC(c2ccccc2)c2ccccc2)cc1)(C(F)(F)F)C(F)(F)F	1	0.35	13.54	-12.87	-14.25
25	FC(F)(F)c1ccc(CN(CCCOc2cccc3ccccc23)CC(c2ccccc2)c2ccccc2)cc1	1	0.41	13.54	-12.70	-14.43
26	FC(F)(F)c1ccc(CN(CCCOc2cccc3c2[CH]CC3)CC(c2ccccc2)c2ccccc2)cc1Cl	1	0.43	13.47	-12.44	-14.57
27	FC(F)(F)c1ccc(CN(CCCOc2cccc3c2CC[CH]3)CC(c2ccccc2)c2ccccc2)cc1Cl	3	0.44	13.46	-12.62	-14.37
28	O=C(O)C1CCCc2ccc(OCCCN(Cc3ccc(C(F)(F)F)c(Cl)c3Cl)CC(c3ccccc3)c3ccccc3)cc21	1	0.55	13.46	-12.52	-14.48
29	O=C1CCCc2c(OCCCN(Cc3ccc(C(F)(F)F)c(Cl)c3)CC(c3ccccc3)c3ccccc3)cccc21	2	0.43	13.45	-12.32	-14.68
30	FC(F)(F)c1ccc(CN(CCCOc2cccc3c2CCCC3)CC(c2ccccc2)c2ccccc2)cc1Cl	2	0.44	13.44	-12.47	-14.50
31	CS(=O)(=O)c1cccc(-c2ccc(CN(Cc3ccc(C(F)(F)F)c(Cl)c3)CC(c3ccccc3)c3ccccc3)cc2)c1	12	0.40	13.43	-12.03	-14.99
32	CS(=O)(=O)c1cc(-c2ccc(CN(Cc3ccc(C(F)(F)F)c(Cl)c3)CC(c3ccccc3)c3ccccc3)cc2)ccc1CO	1	0.37	13.41	-12.48	-14.41
33	O=C1CCc2cc(OCCCN(Cc3ccc(C(F)(F)F)c(Cl)c3)CC(c3ccccc3)c3ccccc3)ccc2N1	1	0.46	13.38	-12.17	-14.71
34	CN1c2ccc(OCCCN(Cc3cccc(C(F)(F)F)c3Cl)CC(c3ccccc3)c3ccccc3)cc2CCC1C(=O)O	10	0.62	13.37	-12.37	-14.45
35	O=C(O)C1CCc2cc(OCCCN(Cc3cccc(C(F)(F)F)c3Cl)c3ccccc3)ccc2C1	15	0.46	13.35	-13.21	-13.49
36	FC(F)(F)c1cccc(CN(CCCOc2cccc3c2CC[CH]3)CC(c2ccccc2)c2ccccc2)c1Cl	1	0.57	13.34	-12.13	-14.67
37	O=C1CCCc2cc(OCCCN(Cc3ccc(Cl)c(C(F)(F)F)c3)CC(c3ccccc3)c3ccccc3)ccc21	16	0.47	13.34	-12.26	-14.52
38	O=C1CCc2cc(OCCCN(Cc3ccc(Cl)c(C(F)(F)F)c3)CC(c3ccccc3)c3ccccc3)ccc2N1	1	0.46	13.34	-12.28	-14.49
39	O=C1CCCc2cc(OCCCN(Cc3cccc(C(F)(F)F)c3Cl)CC(c3ccccc3)c3ccccc3)ccc21	12	0.63	13.34	-11.99	-14.84
40	O=C(O)C1CCc2cc(OCCCN(Cc3ccc(C(F)(F)F)c(Cl)c3)CC(c3ccccc3)c3ccccc3)ccc2N1	1	0.47	13.32	-12.20	-14.54
41	FC(F)(F)c1ccc(CN(CCCOc2cccc3c2CCCC3)CC(c2ccccc2)c2ccccc2)cc1	4	0.38	13.31	-12.35	-14.34
42	CC(C)(C)c1cccc(CN(CCCOc2cccc3c2CCCC3)CC(c2ccccc2)c2ccccc2)c1	1	0.35	13.29	-12.30	-14.36
43	O=C1CCc2ccc(OCCCN(Cc3cccc(C(F)(F)F)c3Cl)CC(c3ccccc3)c3ccccc3)cc2N1	1	0.62	13.28	-11.98	-14.71
44	Clc1ccc(CN(CCCOc2ccc3c(c2)CCCC3)CC(c2ccccc2)c2ccccc2)c(Cl)c1Cl	6	0.46	13.25	-12.39	-14.17
45	O=C1CCc2cc(OCCCN(Cc3cccc(C(F)(F)F)c3Cl)CC(c3ccccc3)c3ccccc3)ccc2N1	15	0.62	13.23	-11.81	-14.83
46	O=C1CCc2cc(OCCCN(Cc3ccc(Cl)c(Cl)c3Cl)CC(c3ccccc3)c3ccccc3)ccc2N1	2	0.44	13.23	-12.16	-14.40
47	O=C(O)Cc1cccc(OCCCN(Cc2ccc(-c3ccc(Cl)c(C(F)(F)F)c3)cc2)CC(c2ccccc2)c2ccccc2)c1	1	0.71	13.23	-12.88	-13.58
48	CS(=O)(=O)c1cccc(Oc2cccc(-c3ccc(CN(Cc4cccc(C(F)(F)F)c4Cl)CC(c4ccccc4)c4ccccc4)cc3)c2)c1	1	0.52	13.21	-12.52	-13.95
49	O=C1CCc2cc(OCCCN(Cc3cccc(C(F)(F)F)c3Cl)CC(c3ccccc3)c3ccccc3)ccc21	2	0.65	13.21	-12.11	-14.42
50	NC(=O)Cc1ccc(OCCCN(Cc2ccc(Cl)c(C(F)(F)F)c2)CC(c2ccccc2)c2ccccc2)cc1	3	0.55	13.21	-12.72	-13.71
51	O=C(O)Cc1cccc2c(OCCCN(Cc3ccc(C(F)(F)F)c(Cl)c3)CC(c3ccccc3)c3ccccc3)cccc12	3	0.57	13.20	-12.64	-13.78
52	CN1C(=O)Cc2cc(OCCCN(Cc3ccc(C(F)(F)F)c(Cl)c3Cl)CC(c3ccccc3)c3ccccc3)ccc2C1=O	1	0.52	13.19	-12.28	-14.16
53	CC1(c2ccc(C(F)(F)F)cc2)C(=O)N(CCCOc2cccc(CC(=O)O)c2)CC1c1ccccc1	1	0.37	13.18	-12.66	-13.71
54	FC(F)(F)c1ccc(CN(CCCOc2ccc3c(c2)CC[CH]3)CC(c2ccccc2)c2ccccc2)cc1Cl	8	0.47	13.17	-12.33	-14.07
55	FC(F)(F)c1cc(CN(CCCOc2cccc3c2CC[CH]3)CC(c2ccccc2)c2ccccc2)ccc1Cl	1	0.44	13.17	-12.15	-14.28
56	O=C(O)C1CCCc2cc(OCCCN(Cc3ccc(Cl)c(C(F)(F)F)c3)CC(c3ccccc3)c3ccccc3)ccc21	8	0.49	13.17	-12.48	-13.90
57	O=C(O)Cc1cccc(OCCCN(CC(c2ccccc2)c2cccc(C(F)(F)F)c2)c2ccccc2)c1	1	0.59	13.16	-12.71	-13.62
58	O=C(O)C1CCc2cc(OCCCN(Cc3ccc(C(F)(F)F)c(Cl)c3)CC(c3ccccc3)c3ccccc3)ccc21	2	0.51	13.16	-11.93	-14.51
59	FC(F)(F)c1cccc(CN(CCCOc2ccc3c(c2)CC[CH]3)CC(c2ccccc2)c2ccccc2)c1Cl	4	0.63	13.14	-12.11	-14.26
60	CN1c2ccc(OCCCN(Cc3ccc(Cl)c(C(F)(F)F)c3)CC(c3ccccc3)c3ccccc3)cc2CCC1C(=O)O	4	0.47	13.13	-12.62	-13.67
61	CN1c2cc(OCCCN(Cc3ccc(C(F)(F)F)cc3)CC(c3ccccc3)c3ccccc3)ccc2CCC1C(=O)O	1	0.42	13.12	-12.16	-14.17
62	O=C(O)Cc1cccc(OCCCN(Cc2ccc(C(F)(F)F)c(Cl)c2)CC(c2ccccc2)c2ccccc2)c1	280	0.75	13.12	-12.46	-13.82
63	O=C(c1ccccc1)N(CCCOc1cccc2c1CCCC2)Cc1ccc(C(F)(F)F)c(Cl)c1Cl	1	0.37	13.12	-12.87	-13.37
64	COC(=O)C(C)(c1cccc(-c2ccc(CN(Cc3ccc(C(F)(F)F)c(Cl)c3)CC(c3ccccc3)c3ccccc3)cc2)c1)C(C)C	1	0.38	13.12	-12.56	-13.70
65	FC(F)(F)c1cccc(CN(CCCOc2ccc3c(c2)CCCC3)CC(c2ccccc2)c2ccccc2)c1Cl	26	0.66	13.12	-11.90	-14.46
66	CN(C)C(=O)c1ccc(CN(Cc2ccc(Cl)c(C(F)(F)F)c2)CC(c2ccccc2)c2ccccc2)cc1	1	0.41	13.11	-12.42	-13.85
67	O=C1CCCc2cc(OCCCN(Cc3cccc(C(F)(F)F)c3)CC(c3ccccc3)c3ccccc3)ccc21	17	0.45	13.11	-11.81	-14.56
68	O=C(O)Cc1cccc(OCCCN(Cc2ccc(Cl)c(C(F)(F)F)c2Cl)CC(c2ccccc2)c2ccccc2)c1	3	0.78	13.11	-12.19	-14.09
69	O=C(O)Cc1cccc(OCCCN(Cc2ccc(F)c(C(F)(F)F)c2Cl)CC(c2ccccc2)c2ccccc2)c1	1	0.77	13.09	-12.10	-14.17
70	O=C1CCCc2cc(OCCCN(Cc3ccc(C(F)(F)F)cc3)CC(c3ccccc3)c3ccccc3)ccc21	6	0.43	13.09	-12.13	-14.12
71	FC(F)(F)c1cccc(CN(CCCOc2cccc3c2CC[CH]3)CC(c2ccccc2)c2ccccc2)c1	1	0.41	13.08	-12.01	-14.25
72	O=C1CCCc2cc(OCCCN(Cc3ccc(Cl)cc3)CC(c3ccccc3)c3ccccc3)ccc21	1	0.41	13.08	-12.17	-14.06
73	NC(=O)Cc1cccc(OCCCN(Cc2ccc(Cl)c(C(F)(F)F)c2)CC(c2ccccc2)c2ccccc2)c1	23	0.66	13.07	-12.36	-13.83
74	FC(F)(F)c1cccc(CN(CCCOc2cccc3[nH]ccc23)CC(c2ccccc2)c2ccccc2)c1	1	0.41	13.07	-12.14	-14.07
75	O=C1CCc2cc(OCCCN(Cc3ccc(C(F)(F)F)cc3)CC(c3ccccc3)c3ccccc3)ccc2N1	6	0.43	13.06	-12.03	-14.18
76	O=C(C1CCCc2cc(OCCCN(Cc3cccc(C(F)(F)F)c3)CC(c3ccccc3)c3ccccc3)ccc21)N1CCCC1	1	0.43	13.06	-12.92	-13.19
77	FC(F)(F)c1cc(CN(CCCOc2ccc3c(c2)CCCC3)CC(c2ccccc2)c2ccccc2)ccc1Cl	1	0.49	13.05	-12.10	-14.07
78	CC(c1ccc(C(F)(F)F)c(Cl)c1)N(CCCOc1ccc2c(c1)CCC(C(=O)O)N2C)CC(c1ccccc1)c1ccccc1	1	0.38	13.04	-12.87	-13.22
79	CN(C(=O)N(CCCOc1cccc2c1CCCC2)Cc1cccc(C(F)(F)F)c1Cl)C(c1ccccc1)c1ccccc1	1	0.44	13.04	-12.36	-13.76
80	FC(F)(F)c1cccc(CN(CCCOc2cccc3c2CCCC3)CC(c2ccccc2)c2ccccc2)c1Cl	1	0.57	13.02	-11.49	-14.75

81	FC(F)(F)c1ccc(CN(CCCOc2ccc3c(c2)CCCC3)CC(c2ccccc2)c2ccccc2)cc1	17	0.45	13.02	-11.97	-14.15
82	CC1CCc2cc(OCCCN(Cc3ccc(Cl)c(C(F)(F)F)c3)CC(c3ccccc3)c3ccccc3)ccc21	5	0.47	13.01	-12.04	-14.06
83	FC(F)(F)c1ccc(CN(CCCOc2ccc3c(c2)CCCC3)CC(c2ccccc2)c2ccccc2)cc1Cl	3	0.49	13.01	-11.90	-14.22
84	O=C1CCc2cc(OCCCN(Cc3ccc(C(F)(F)F)c(Cl)c3)CC(c3ccccc3)c3ccccc3)ccc21	4	0.48	13.00	-11.99	-14.11
85	FC(F)(F)c1ccc(CN(CCCOc2ccc3c(c2)CCCCC3)CC(c2ccccc2)c2ccccc2)cc1Cl	1	0.49	13.00	-12.75	-13.25
86	O=C(O)Cc1cc2cc(OCCCN(Cc3cccc(C(F)(F)F)c3)CC(c3ccccc3)c3ccccc3)ccc2[nH]1	1	0.53	12.97	-11.99	-14.03
87	CN1c2ccc(OCCCN(Cc3ccc(C(F)(F)F)c(Cl)c3)CC(c3ccccc3)c3ccccc3)cc2CCC1C(=O)O	3	0.47	12.97	-12.29	-13.68
88	CN1c2ccc(OCCCN(Cc3ccc(C(F)(F)F)cc3)CC(c3ccccc3)c3ccccc3)cc2CCC1C(=O)O	10	0.42	12.97	-12.49	-13.46
89	CC1CCc2cc(OCCCN(Cc3ccc(C(F)(F)F)c(Cl)c3)CC(c3ccccc3)c3ccccc3)ccc2NC1=O	1	0.44	12.95	-12.51	-13.41
90	O=C(O)Cc1cccc2c(OCCCN(Cc3ccc(Cl)c(C(F)(F)F)c3)CC(c3ccccc3)c3ccccc3)cccc12	3	0.57	12.95	-11.79	-14.23
91	CC1CCc2cc(OCCCN(Cc3cccc(C(F)(F)F)c3)CC(c3ccccc3)c3ccccc3)ccc21	1	0.44	12.94	-12.27	-13.64
92	O=C1CCc2ccc(OCCCN(Cc3ccc(Cl)c(C(F)(F)F)c3)CC(c3ccccc3)c3ccccc3)cc21	1	0.48	12.93	-11.90	-14.06
93	O=C(O)Cc1ccccc1OCCCN(Cc1cccc(C(F)(F)F)c1Cl)CC(c1ccccc1)c1ccccc1	2	0.75	12.93	-12.31	-13.58
94	CC(C)(C)c1cccc(CN(CCCOc2ccc3c(c2)CCCC3)CC(c2ccccc2)c2ccccc2)c1	1	0.41	12.93	-12.02	-13.90
95	FC(F)(F)c1cc(CN(CCCOc2ccc3c(c2)CC[CH]3)CC(c2ccccc2)c2ccccc2)ccc1Cl	6	0.47	12.92	-11.83	-14.12
96	NC(=O)C1CCCc2cc(OCCCN(Cc3ccc(C(F)(F)F)cc3)CC(c3ccccc3)c3ccccc3)ccc21	1	0.44	12.92	-12.08	-13.82
97	O=C(O)Cc1cccc(OCCCN(Cc2ccc(Cl)c(Cl)c2)CC(c2ccccc2)c2ccccc2)c1	1	0.67	12.91	-12.55	-13.28
98	FC(F)(F)c1cccc(CN(CCCOc2ccc3c(c2)CCCCC3)CC(c2ccccc2)c2ccccc2)c1Cl	1	0.65	12.91	-11.89	-14.02
99	c1ccc(CN(CCCOc2ccc3c(c2)CCCC3)CC(c2ccccc2)c2ccccc2)cc1	4	0.43	12.91	-12.51	-13.32
100	CS(=O)(=O)c1cccc(Oc2cccc(-c3ccc(CN(Cc4ccc(C(F)(F)F)c(Cl)c4)CC(c4ccccc4)c4ccccc4)cc3)c2)c1	2	0.39	12.90	-12.31	-13.52
101	O=C1CCc2ccc(OCCCN(Cc3ccc(Cl)c(C(F)(F)F)c3)CC(c3ccccc3)c3ccccc3)cc2N1	2	0.46	12.89	-11.59	-14.33
102	O=C(O)C1CCCc2cc(OCCCN(Cc3ccc(C(F)(F)F)c(Cl)c3)CC(c3ccccc3)c3ccccc3)ccc21	16	0.49	12.88	-12.28	-13.51
103	NC(=O)Cc1cccc(OCCCN(CC2=CC=CC=CC(C(F)(F)F)=C2Cl)CC(c2ccccc2)c2ccccc2)c1	3	0.56	12.87	-11.92	-13.89
104	O=C(O)Cc1cccc(OCCCC(CC(c2ccccc2)c2ccccc2)c2ccc(C(F)(F)F)c(Cl)c2)c1	1	0.56	12.86	-11.56	-14.31
105	O=C(O)Cc1cccc(OCCCN(Cc2ccc(Cl)c(Cl)c2Cl)CC(c2ccccc2)c2ccccc2)c1	37	0.75	12.86	-12.15	-13.60
106	O=C(O)Cc1ccc(OCCCN(Cc2ccccc2C(F)(F)F)CC(c2ccccc2)c2ccccc2)cc1	1	0.70	12.85	-12.34	-13.38
107	FC(F)(F)c1cccc(CN(CCCOc2ccc3c(c2)CC[CH]3)CC(c2ccccc2)c2ccccc2)c1	3	0.45	12.85	-11.92	-13.86
108	COC(=O)C1CCc2cc(OCCCN(Cc3cccc(C(F)(F)F)c3Cl)CC(c3ccccc3)c3ccccc3)ccc21	1	0.62	12.85	-11.94	-13.83
109	CN(C)C(=O)c1ccc(N2CCC(C(c3ccccc3)c3ccc(C(F)(F)F)c(Cl)c3Cl)CC2)cc1	1	0.37	12.85	-12.16	-13.58
110	O=C(O)Cc1ccccc1OCCCN(Cc1ccc(Cl)c(C(F)(F)F)c1)CC(c1ccccc1)c1ccccc1	2	0.59	12.82	-12.08	-13.61
111	Clc1ccc(CN(CCCOc2ccc3c(c2)CCCC3)CC(c2ccccc2)c2ccccc2)cc1	2	0.42	12.82	-11.75	-13.99
112	O=C(O)Cc1cccc2c(OCCCN(Cc3cccc(C(F)(F)F)c3)Cc3ccc(C(F)(F)F)c(Cl)c3)cccc12	1	0.44	12.82	-12.28	-13.37
113	O=C(O)Cc1cccc2c(OCCCN(Cc3ccc(C(F)(F)F)c(Cl)c3)c3ccccc3)cccc12	2	0.42	12.80	-12.76	-12.85
114	CC(C)(C)OC(=O)C1CCc2cc(OCCCN(Cc3ccc(C(F)(F)F)c(Cl)c3)CC(c3ccccc3)c3ccccc3)ccc21	3	0.47	12.80	-12.07	-13.58
115	NC(=O)C1CCCc2cc(OCCCN(Cc3ccc(C(F)(F)F)c(Cl)c3)CC(c3ccccc3)c3ccccc3)ccc21	1	0.48	12.80	-11.70	-14.00
116	FC(F)(F)c1ccc(CN(CCCOc2ccc3[nH]ccc3c2)CC(c2ccccc2)c2ccccc2)cc1Cl	2	0.47	12.79	-12.10	-13.53
117	COC(=O)C1CCc2cc(OCCCN(Cc3ccc(C(F)(F)F)c(Cl)c3)CC(c3ccccc3)c3ccccc3)ccc21	3	0.48	12.76	-11.97	-13.60
118	NC(=O)Cc1cccc(OCCCN(Cc2ccc(C(F)(F)F)c(Cl)c2Cl)CC(c2ccccc2)c2ccccc2)c1	3	0.77	12.76	-12.04	-13.52
119	NC(=O)Cc1cccc(OCCCN(Cc2ccc(Cl)c(Cl)c2Cl)CC(c2ccccc2)c2ccccc2)c1	16	0.65	12.75	-12.00	-13.56
120	FC(F)(F)c1ccc(CN(CCCOc2ccc3c(c2)CC[CH]3)CC(c2ccccc2)c2ccccc2)c(Cl)c1Cl	1	0.55	12.75	-11.86	-13.71
121	O=C(O)C1CCCc2cc(OCCCN(Cc3ccc(C(F)(F)F)cc3)CC(c3ccccc3)c3ccccc3)ccc21	1	0.45	12.75	-12.30	-13.21
122	CN(C)C(=O)c1ccc(N2CCC(C(c3ccccc3)c3ccc(C(F)(F)F)c(Cl)c3)C2)cc1	1	0.38	12.75	-12.23	-13.30
123	O=C(O)Cc1ccc(OCCCN(Cc2ccc(C(F)(F)F)cc2Cl)CC(c2ccccc2)c2ccccc2)cc1	1	0.61	12.75	-11.88	-13.68
124	CC1CCc2cc(OCCCN(Cc3ccc(C(F)(F)F)cc3)CC(c3ccccc3)c3ccccc3)ccc21	5	0.43	12.74	-11.75	-13.83
125	O=C1CCCc2cc(OCCCN(Cc3ccc(Cl)c(C(F)(F)F)c3)Cc3cccc(C(F)(F)F)c3Cl)ccc21	1	0.46	12.74	-11.96	-13.58
126	CC(C)(C)C(C)(c1cccc(Oc2cccc(S(C)(=O)=O)c2)c1)c1cccc(C(F)(F)F)c1Cl	1	0.35	12.74	-12.14	-13.37
127	CC(C)(C)c1ccc(CN(CCCOc2ccc3c(c2)CCCC3)CC(c2ccccc2)c2ccccc2)cc1	2	0.39	12.74	-11.74	-13.81
128	O=C(O)CC(c1ccccc1)c1cccc(-c2ccc(CN(Cc3cccc(C(F)(F)F)c3Cl)CC(c3ccccc3)c3ccccc3)cc2)c1	1	0.58	12.72	-12.49	-12.95
129	COC(=O)C1CCc2cc(OCCCN(Cc3ccc(Cl)c(C(F)(F)F)c3)CC(c3ccccc3)c3ccccc3)ccc21	1	0.48	12.71	-11.75	-13.74
130	O=C(O)Cc1ccc(OCCCN(Cc2ccc(C(F)(F)F)c(Cl)c2Cl)CC(c2ccccc2)c2ccccc2)cc1	15	0.72	12.71	-11.74	-13.75
131	O=C(O)Cc1cccc(OCCCN(Cc2ccc(C(F)(F)F)c(Cl)c2Cl)CC(c2ccccc2)c2ccccc2Cl)c1	1	0.78	12.70	-11.63	-13.87
132	CC(C)CNC(=O)C1CCc2cc(OCCCN(Cc3ccc(C(F)(F)F)c(Cl)c3)C(c3ccccc3)c3ccccc3)ccc21	1	0.36	12.69	-12.23	-13.17
133	O=C(O)C1CCCc2cc(OCCCN(Cc3ccc(C(F)(F)F)c(Cl)c3Cl)CC(c3ccccc3)c3ccccc3)ccc21	1	0.57	12.69	-12.30	-13.09
134	O=C(NCCCOc1cccc2[nH]ccc12)C1CCc2cc(OCCCN(Cc3ccc(C(F)(F)F)c(Cl)c3)CC(c3ccccc3)c3ccccc3)ccc21	1	0.40	12.69	-11.82	-13.62
135	O=C(O)Cc1ccc(OCCCN(Cc2cccc(C(F)(F)F)c2Cl)CC(c2ccccc2)c2ccccc2Cl)cc1	1	0.74	12.67	-11.89	-13.50
136	O=C(O)Cc1ccc(OCCCN(Cc2ccc(Cl)c(Cl)c2Cl)CC(c2ccccc2)c2ccccc2)cc1	19	0.60	12.67	-11.83	-13.57
137	CC1CCc2cc(OCCCN(Cc3cccc(C(F)(F)F)c3Cl)CC(c3ccccc3)c3ccccc3)ccc21	3	0.62	12.67	-11.40	-14.09
138	CS(=O)(=O)c1cccc(Oc2cccc(-c3ccc(CN(Cc4ccccc4Cl)c4cccc(C(F)(F)F)c4Cl)cc3)c2)c1	1	0.36	12.66	-12.78	-12.54
139	O=C(O)Cc1cccc(OCCCN(Cc2cccc(Cl)c2Cl)CC(c2ccccc2)c2ccccc2)c1	1	0.79	12.66	-11.91	-13.46
140	O=C(O)Cc1cccc(OCCCN(CC(c2ccccc2)c2ccccc2)C(=O)c2ccccc2Cl)c1	1	0.57	12.66	-11.54	-13.88
141	CC(C)(C)c1cc(CN(CCCOc2cccc(CC(=O)O)c2)CC(c2ccccc2)c2ccccc2)cc(Cl)c1Cl	1	0.67	12.66	-12.16	-13.17
142	O=C(O)Cc1cccc(OCCCN(Cc2ccc(C(F)(F)F)c(Cl)c2Cl)CC(c2ccccc2)c2ccccc2)c1	21	0.89	12.65	-11.58	-13.83
143	O=C(O)Cc1cccc(OCCCN(Cc2ccc(-c3ccc(C(F)(F)F)cc3)cc2)CC(c2ccccc2)c2ccccc2)c1	1	0.67	12.65	-12.14	-13.19
144	O=C1CCCc2ccc(OCCCN(Cc3ccc(C(F)(F)F)c(Cl)c3)CC(c3ccccc3)c3ccccc3)cc21	2	0.47	12.64	-11.74	-13.61
145	CCN(CC(c1ccccc1)c1ccccc1)N(CCCOc1cccc2c1CCCC2=O)Cc1ccc(C(F)(F)F)c(Cl)c1	1	0.36	12.63	-11.78	-13.55
146	NC(=O)Cc1cccc(OCCCN(CC(c2ccccc2)c2ccccc2)C(=O)c2ccccc2Cl)c1	1	0.49	12.63	-11.61	-13.75
147	O=C(O)Cc1cccc2c(OCCCN(Cc3ccc(Cl)c(C(F)(F)F)c3)c3ccccc3)cccc12	1	0.42	12.60	-12.22	-13.01
148	CN(C)C(=O)C1CCCc2cc(OCCCN(Cc3ccc(C(F)(F)F)c(Cl)c3)C(c3ccccc3)c3ccccc3)ccc21	3	0.38	12.59	-12.84	-12.35
149	CC1CCc2cc(OCCCN(Cc3ccc(C(F)(F)F)c(Cl)c3)CC(c3ccccc3)c3ccccc3)ccc21	20	0.47	12.59	-11.73	-13.52
150	CCC(c1cccc(C(F)(F)F)c1)N(CCCOc1cccc(CC(N)=O)c1)CC(c1ccccc1)c1ccccc1	1	0.49	12.59	-11.93	-13.29
151	O=S(=O)(c1ccc(F)cc1)N(Cc1ccccc1)Cc1ccc(-c2ccc(C(O)(C(F)(F)F)C(F)(F)F)cc2)cc1	1	0.51	12.59	-13.05	-12.15
152	CC(C)(C)c1cc(N(CCCOc2cccc(CC(=O)O)c2)CC(c2ccccc2)c2ccccc2)cc(C(F)(F)F)c1Cl	2	0.65	12.59	-12.30	-12.89
153	O=C(O)C1CCCc2cc(OCCCN(Cc3cccc(C(F)(F)F)c3)CC(c3ccccc3)c3ccccc3)ccc21	6	0.46	12.59	-11.93	-13.28
154	FC(F)(F)c1ccc(CN(CCCOc2ccc3[nH]ccc3c2)CC(c2ccccc2)c2ccccc2)cc1	1	0.43	12.58	-11.71	-13.52
155	O=C(O)Cc1ccccc1OCCCN(Cc1ccc(C(F)(F)F)c(Cl)c1)CC(c1ccccc1)c1ccccc1	2	0.59	12.57	-11.73	-13.48
156	CN(C)C(=O)c1ccc(N2CCC(C(c3ccccc3)c3ccc(Cl)c(C(F)(F)F)c3)C2)cc1	1	0.38	12.57	-11.85	-13.32

157	O=C(O)Cc1ccc(OCCCN(Cc2ccc(Cl)c(Cl)c2)CC(c2ccccc2)c2ccccc2)cc1	1	0.55	12.56	-11.92	-13.24
158	O=C(O)Cc1ccc(OCCCN(CC(c2ccccc2)c2ccccc2)C(=O)c2cccc(Cl)c2Cl)cc1	2	0.46	12.55	-11.64	-13.53
159	NC(=O)Cc1cccc(OCCCN(Cc2ccc(Cl)c(C(F)(F)F)c2)CC(c2ccccc2)c2ccccc2Cl)c1	1	0.59	12.55	-11.42	-13.79
160	NC(=O)C1CCCc2cc(OCCCN(Cc3ccc(Cl)c(C(F)(F)F)c3)CC(c3ccccc3)c3ccccc3)ccc21	1	0.48	12.55	-12.27	-12.84
161	O=C1CCCc2cc(OCCCN(Cc3cc(C(F)(F)F)cc(C(F)(F)F)c3)CC(c3ccccc3)c3ccccc3)ccc21	1	0.43	12.55	-11.30	-13.93
162	O=C(O)Cc1cccc(OCCCN(Cc2ccc(C(F)(F)F)cc2Cl)CC(c2ccccc2)c2ccccc2)c1	2	0.74	12.54	-11.51	-13.67
163	CN1c2ccc(OCCCN(Cc3cccc(C(F)(F)F)c3)CC(c3ccccc3)c3ccccc3)cc2CCC1C(=O)O	2	0.45	12.53	-11.29	-13.90
164	CC(C)(CN(CCCOc1cccc(CC(=O)O)c1)CC(c1ccccc1)c1ccccc1)c1ccccc1	2	0.60	12.53	-11.81	-13.28
165	CS(=O)(=O)c1cccc(Oc2cccc(-c3ccccc3-c3ccc(-c4cc(C(F)(F)F)c(Cl)c(C(F)(F)F)c4)cc3)c2)c1	1	0.40	12.52	-14.05	-11.16
166	O=C(O)Cc1cccc(OCCCN(CC(c2ccccc2)c2ccccc2)C(=O)c2cccc(Cl)c2Cl)c1	3	0.57	12.52	-11.44	-13.70
167	O=C(O)Cc1ccc(OCCCN(Cc2cccc(C(F)(F)F)c2Cl)CC(c2ccccc2)c2ccccc2)cc1	333	0.84	12.51	-11.41	-13.72
168	CC(C)(C)c1cccc(CN(CCCOc2cccc(CC(N)=O)c2)CC(c2ccccc2)c2ccccc2)c1	2	0.54	12.51	-11.42	-13.71
169	CN(C(=O)CCC(c1ccccc1)c1ccccc1)c1ccc(C(O)(C(F)(F)F)C(F)(F)F)cc1	2	0.43	12.51	-12.77	-12.26
170	NC(=O)Cc1ccc(OCCCN(Cc2ccc(C(F)(F)F)c(Cl)c2)CC(c2ccccc2)c2ccccc2)cc1	3	0.55	12.51	-11.34	-13.79
171	OC1(C(F)(F)F)C=Cc2ccc(OCCCN(CC(c3ccccc3)c3ccccc3)CC(c3ccccc3)c3ccccc3)cc21	1	0.41	12.51	-12.77	-12.25
172	CC(C)(C)c1cc(CN(CCCOc2cccc(CC(=O)O)c2)CC(c2ccccc2)c2ccccc2)ccc1Cl	1	0.65	12.51	-11.58	-13.50
173	CN(C(=O)N(CCCOc1ccc2c(c1)CCCC2)Cc1ccc(C(F)(F)F)cc1)C(c1ccccc1)c1ccccc1	1	0.35	12.49	-11.95	-13.06
174	OC(c1ccc(N(Cc2ccccc2)CC(c2ccccc2)c2ccccc2)cc1)(C(F)(F)F)C(F)(F)F	6	0.42	12.49	-11.40	-13.68
175	NC(=O)Cc1cccc(OCCCN(Cc2ccc(C(F)(F)F)c(Cl)c2)CC(c2ccccc2)c2ccccc2)c1	50	0.66	12.48	-11.44	-13.61
176	CC(C)(C)c1ccc(CN(CCCOc2cccc(CC(=O)O)c2)CC(c2ccccc2)c2ccccc2)cc1	36	0.63	12.47	-11.75	-13.23
177	CN(C)C(=O)c1ccc(CN(CCCOc2cccc(CC(=O)O)c2)CC(c2ccccc2)c2ccccc2)cc1	2	0.59	12.46	-11.73	-13.24
178	FC(F)(F)c1cccc(CN(CCCOc2ccc3c(c2)CCCCC3)c2ccccc2)c1Cl	2	0.46	12.46	-12.00	-12.94
179	O=C(O)Cc1cccc(OCCCN(Cc2ccc(Cl)cc2Cl)CC(c2ccccc2)c2ccccc2)c1	2	0.69	12.46	-11.58	-13.42
180	O=C(O)C1CCCc2cc(OCCCN(Cc3ccc(C(F)(F)F)c(Cl)c3Cl)c3ccccc3)ccc21	1	0.41	12.46	-12.51	-12.41
181	CC(C)(C)c1ccccc1CN(CCCOc1cccc(CC(=O)O)c1)CC(c1ccccc1)c1ccccc1	7	0.75	12.46	-11.15	-13.92
182	NC(=O)Cc1ccc(OCCCN(Cc2cccc(C(F)(F)F)c2Cl)CC(c2ccccc2)c2ccccc2)cc1	8	0.73	12.45	-11.39	-13.61
183	O=C(O)Cc1ccc(OCCCN(Cc2ccc(Cl)c(C(F)(F)F)c2)CC(c2ccccc2)c2ccccc2)cc1	66	0.63	12.45	-11.19	-13.84
184	CC(C)(C)c1cccc(CN(CCCOc2ccc(CC(=O)O)cc2)CC(c2ccccc2)c2ccccc2)c1	11	0.54	12.45	-11.57	-13.39
185	NC(=O)Cc1ccc(OCCCN(Cc2ccc(C(F)(F)F)c(Cl)c2Cl)CC(c2ccccc2)c2ccccc2)cc1	1	0.63	12.44	-11.26	-13.74
186	O=C(O)Cc1cccc(OCCCN(Cc2cccc(C(F)(F)F)c2Cl)CC(c2ccccc2)c2cccc(Cl)c2Cl)c1	5	0.86	12.43	-11.89	-13.00
187	CC(C)(C)c1ccc(CN(CCCOc2ccc(CC(=O)O)cc2)CC(c2ccccc2)c2ccccc2)cc1Cl	1	0.54	12.42	-11.87	-13.01
188	CN1CCC(C(=O)N2CCN(c3ccc(CO)c(S(C)(=O)=O)c3)CC2)(c2ccccc2)CCC1C(F)(F)F	1	0.40	12.41	-12.93	-11.92
189	NC(=O)Cc1cccc(OCCCN(Cc2ccc(C(F)(F)F)cc2Cl)CC(c2ccccc2)c2ccccc2)c1	1	0.65	12.41	-11.21	-13.74
190	NC(=O)Cc1ccc(OCCCN(Cc2ccc(Cl)c(Cl)c2Cl)CC(c2ccccc2)c2ccccc2)cc1	1	0.51	12.41	-11.45	-13.45
191	NC(=O)Cc1cccc(OCCCN(Cc2ccc(-c3ccccc3)cc2)CC(c2ccccc2)c2ccccc2)c1	1	0.55	12.41	-11.88	-12.97
192	O=C(O)Cc1cccc(OCCCN(Cc2cccc(C(F)(F)F)c2)CC(c2ccccc2)c2ccccc2)c1	101	0.70	12.41	-11.23	-13.71
193	CN1c2ccc(OCCCN(Cc3ccc(C(F)(F)F)cc3)Cc3cccc(C(F)(F)F)c3Cl)cc2CCC1C(=O)O	1	0.48	12.41	-11.72	-13.13
194	CN(C)C(=O)C1CCCc2cc(OCCCN(Cc3ccc(C(F)(F)F)c(Cl)c3)CC(c3ccccc3)c3ccccc3)ccc21	3	0.47	12.39	-12.75	-12.05
195	COC(=O)C1CCc2cc(OCCCN(Cc3ccc(C(F)(F)F)cc3)CC(c3ccccc3)c3ccccc3)ccc21	1	0.44	12.38	-11.50	-13.33
196	FC(F)(F)c1cccc(CN(CCCOc2ccc3c(c2)CCCC3)c2ccccc2)c1Cl	13	0.47	12.38	-12.00	-12.78
197	O=C(O)Cc1ccc(OCCCN(CC2=CC=CC=CC(C(F)(F)F)=C2Cl)CC(c2ccccc2)c2ccccc2)cc1	4	0.52	12.38	-11.43	-13.41
198	FC(F)(F)c1ccc(CN(CCCOc2ccc3c(c2)CC[CH]3)CC(c2ccccc2)c2ccccc2)cc1	7	0.43	12.36	-11.07	-13.82
199	CN(CCC(c1ccccc1)c1ccccc1)C(=O)C1CCCc2cc(OCCCN(Cc3cccc(C(F)(F)F)c3)c3ccccc3)ccc21	1	0.34	12.36	-12.75	-11.99
200	O=C(O)Cc1ccc(OCCCNC(=O)C2CCCc3cc(OCCCN(Cc4ccc(C(F)(F)F)cc4)CC(c4ccccc4)c4ccccc4)ccc32)cc1	1	0.46	12.36	-11.76	-12.99

Table S3. Fine-tuning set I.

ID	SMILES
1	COc1ccccc1C1SC(c2ccc(Cl)cc2)=NN1C(=O)c1c(F)cc(F)cc1F
2	COc1cc(F)ccc1C1SC(c2ccc(F)cc2)=NN1C(=O)c1c(F)cc(F)cc1F
3	COc1ncccc1C1SC(c2ccc(F)cc2)=NN1C(=O)c1c(F)cc(F)cc1F
4	COc1cc(F)c(C(=O)N2N=C(c3ccc(F)cc3)SC2c2cccc(OC)c2OC)c(F)c1
5	COc1cccc(C2SC(c3ccc(F)cc3)=NN2C(=O)c2cc(F)c(F)c(OC)c2F)c1OC
6	O=S(=O)(c1ccccc1)N(CC(F)(F)F)c1ccc(C(O)(C(F)(F)F)C(F)(F)F)cc1
7	COc1ccc(NC2=C(c3ccccc3)C(=O)N(Cc3ccccc3)C2=O)cc1
8	O=C1C(Nc2ccccc2)=C(c2ccccc2)C(=O)N1Cc1ccccc1
9	CCN1C(=O)C(Nc2ccc(OC)cc2)=C(c2ccccc2)C1=O
10	COc1ccc(N=C2C(=O)N(c3ccccc3)C(=O)C2c2ccccc2)cc1
11	COc1ccc(NC2=C(c3ccccc3)C(=O)N(C)C2=O)cc1
12	CCN1C(=O)C(Nc2ccc(Cl)cc2)=C(c2ccccc2)C1=O
13	C[CH](CC[CH]1OC1(C)C)[CH]1CC[CH]2[CH]3CC=C4C[CH](O)CC[C]4(C)[CH]3CC[C]21C
14	COc1ccc(CN(CCCOc2cccc(C(N)=O)c2)CC(c2ccccc2)c2ccccc2)cc1OC
15	COc1ccc(CN(CCCOc2cccc(CC(N)=O)c2)CC(c2ccccc2)c2ccccc2)cc1
16	COc1ccc(CN(CCCOc2cccc(CC(N)=O)c2)CC(c2ccccc2)c2ccccc2)cc1F
17	COc1ccc(CN(CCCOc2cccc(CC(N)=O)c2)CC(c2ccccc2)c2ccccc2)c(F)c1

18	NC(=O)Cc1cccc(OCCCN(Cc2cccc(C(F)(F)F)c2)CC(c2ccccc2)c2ccccc2)c1
19	NC(=O)Cc1cccc(OCCCN(Cc2ccc(F)c(C(F)(F)F)c2)CC(c2ccccc2)c2ccccc2)c1
20	NC(=O)Cc1cccc(OCCCN(Cc2cccc(C(F)(F)F)c2Cl)CC(c2ccccc2)c2ccccc2)c1
21	CC(C)CCC[C](C)(O)[CH]1CC[CH]2[CH]3CC=C4C[CH](O)CC[C]4(C)[CH]3CC[C]21C
22	CC(C)CC[CH](O)[CH](C)[CH]1CC[CH]2[CH]3CC=C4C[CH](O)CC[C]4(C)[CH]3CC[C]21C
23	CC(C)[CH](O)CC[CH](C)[CH]1CC[CH]2[CH]3CC=C4C[CH](O)CC[C]4(C)[CH]3CC[C]21C
24	C[CH](CO)CCC[CH](C)[CH]1CC[CH]2[CH]3CC=C4C[CH](O)CC[C]4(C)[CH]3CC[C]21C
25	CC(C)C(=O)CC[CH](C)[CH]1CC[CH]2[CH]3CC=C4C[CH](O)CC[C]4(C)[CH]3CC[C]21C
26	C[CH](CCC(=O)N(C)C)[CH]1CC[CH]2[CH]3CC=C4C[CH](O)CC[C]4(C)[CH]3CC[C]21C
27	CNC(=O)CC[CH](C)[CH]1CC[CH]2[CH]3CC=C4C[CH](O)CC[C]4(C)[CH]3CC[C]21C
28	COC(=O)CC[CH](C)[CH]1CC[CH]2[CH]3CC=C4C[CH](O)CC[C]4(C)[CH]3CC[C]21C
29	Cc1cc(OCCCN(Cc2cccc(C(F)(F)F)c2Cl)CC(c2ccccc2)c2ccccc2)[nH]n1
30	COC(=O)c1cc(OCCCN(Cc2cccc(C(F)(F)F)c2Cl)CC(c2ccccc2)c2ccccc2)no1
31	FC(F)(F)c1cccc(CN(CCCOc2n[nH]c3ccccc23)CC(c2ccccc2)c2ccccc2)c1Cl
32	Cc1cc(OCCCN(Cc2cccc(C(F)(F)F)c2Cl)CC(c2ccccc2)c2ccccc2)n2ncnc2n1
33	COC(=O)Cc1c[nH]c2ccc(OCCCN(Cc3cccc(C(F)(F)F)c3Cl)CC(c3ccccc3)c3ccccc3)cc12
34	FC(F)(F)c1cccc(CN(CCCOc2ccc3[nH]ccc3c2)CC(c2ccccc2)c2ccccc2)c1Cl
35	FC(F)(F)c1cccc(CN(CCCOc2cccc3[nH]ccc23)CC(c2ccccc2)c2ccccc2)c1Cl
36	Cn1ccc2c(OCCCN(Cc3cccc(C(F)(F)F)c3Cl)CC(c3ccccc3)c3ccccc3)cccc21
37	CS(=O)(=O)n1ccc2c(OCCCN(Cc3cccc(C(F)(F)F)c3Cl)CC(c3ccccc3)c3ccccc3)cccc21
38	O=C(O)Cn1ccc2c(OCCCN(Cc3cccc(C(F)(F)F)c3Cl)CC(c3ccccc3)c3ccccc3)cccc21
39	N#CCCc1ccc(-c2cc3ccccc3n2C(=O)c2ccccc2)cc1
40	CC(C)(C)OC(=O)n1c(-c2ccc3c(c2)CC(NS(=O)(=O)c2cccs2)C3)cc2ccccc21
41	CC(C)(C)OC(=O)n1c(-c2ccc3c(c2)CC(NS(=O)(=O)c2c(F)cccc2F)C3)cc2ccccc21
42	CC(C)[CH]1CN(c2cccc(S(C)(=O)=O)c2)CCN1C(=O)OC(C)(C)C
43	CC(C)(C)OC(=O)N1CCN(c2cccc(S(C)(=O)=O)c2)C[CH]1Cc1ccccc1
44	CC(C)(C)OC(=O)N1CCN(c2cccc(S(C)(=O)=O)c2)CC1c1ccc(Br)cc1
45	CCC(C)[CH]1CN(c2cccc(S(C)(=O)=O)c2)CCN1Cc1ccccc1
46	CC(C)[CH]1CN(c2ccc(F)c(S(C)(=O)=O)c2)CCN1C(=O)OC(C)(C)C
47	CC(C)[CH]1CN(c2ccc(C(N)=O)c(S(C)(=O)=O)c2)CCN1c1nccc(C(F)(F)F)n1
48	CC(C)[CH]1CN(c2cccc(S(C)(=O)=O)c2)CCN1C(=O)OCC(C)(C)C
49	CCC(C)OC(=O)N1CCN(c2cccc(S(C)(=O)=O)c2)C[CH]1C(C)C
50	CC(C)[CH]1CN(c2ccc(F)c(S(C)(=O)=O)c2)CCN1c1nccc(C(F)(F)F)n1
51	Cc1cc(C)nc(N2CCN(c3cccc(S(C)(=O)=O)c3)C[CH]2C(C)C)n1
52	CC(C)[CH]1CN(c2cccc(S(C)(=O)=O)c2)CCN1c1ncc(Cl)cn1
53	CC(C)[CH]1CN(c2cccc(S(C)(=O)=O)c2)CCN1c1ncc(C2CC2)cn1
54	COC(=O)c1cnc(N2CCN(c3cccc(S(C)(=O)=O)c3)C[CH]2C(C)C)nc1
55	CC(C)[CH]1CN(c2ccc(F)c(S(C)(=O)=O)c2)CCN1c1nccc(C2CC2)n1
56	Cc1nc(N2CCN(c3ccc(F)c(S(C)(=O)=O)c3)C[CH]2C(C)C)ncc1Cl
57	CC(C)(O)[CH]1CN(c2cccc(S(C)(=O)=O)c2)CCN1c1nccc(C(F)(F)F)n1
58	CC(C)(C)C1CN(c2ccc(F)c(S(C)(=O)=O)c2)CCN1c1nccc(C(F)(F)F)n1
59	CC(C)[CH]1CN(c2cccc(S(C)(=O)=O)c2)CCN1c1ccc(C(F)(F)F)cn1
60	CC(C)[CH]1CN(c2ccc(F)c(S(C)(=O)=O)c2)CCN1c1ccc(C(F)(F)F)cn1
61	CC(C)[CH]1CN(c2cccc(S(C)(=O)=O)c2)CCN1c1ccnc(C(F)(F)F)n1
62	CC(C)[CH]1CN(c2cccc(S(C)(=O)=O)c2)CCN1c1nsc(C(F)(F)F)n1
63	CC(C)[CH]1CN(c2cccc(S(N)(=O)=O)c2)CCN1c1nccc(C(F)(F)F)n1

64	CS(=O)(=O)c1cccc(N2CCN(c3nccc(C(F)(F)F)n3)[CH](c3ccc(F)cc3)C2)c1
65	CC(C)[CH]1CN(c2ccc(F)c(S(C)(=O)=O)c2)CCN1c1ncc(C(C)(C)O)c(C(F)(F)F)n1
66	CS(=O)(=O)c1cc(N2CCN(C[CH]3CC[CH](C(F)(F)F)CC3)[CH](c3ccccc3)C2)ccc1C(N)=O
67	CC(C)[CH]1CN(c2ccc(F)c(S(C)(=O)=O)c2Cl)CCN1c1ncc(Cl)c(C2CC2)n1
68	CS(=O)(=O)c1cccc(N2CCN(Cc3ccc(C(F)(F)F)c(Cl)c3)[CH](Cc3ccccc3)C2)c1
69	CS(=O)(=O)c1cccc(N2CCN(Cc3ccc(C(F)(F)F)c(Cl)c3)[CH](c3ccccc3)C2)c1
70	CC(C)c1ccc(CN2CCN(c3cccc(S(C)(=O)=O)c3)C[CH]2c2ccccc2)cc1
71	CS(=O)(=O)c1cccc(N2CCN(Cc3ccc(C(F)(F)F)c(F)c3)[CH](C3CCCCC3)C2)c1
72	C[C]1(c2ccccc2)CN(c2cccc(S(C)(=O)=O)c2)CCN1Cc1ccc(C(F)(F)F)c(Cl)c1
73	CC(C)c1ccc(CN2CCN(c3cccc(S(C)(=O)=O)c3)CC2c2ccccc2)cn1
74	Cc1cc(CN2CCN(c3cccc(S(C)(=O)=O)c3)CC2(C)c2ccccc2)cnc1C(F)(F)F
75	CS(=O)(=O)c1cccc(N2CCN(Cc3ccc(C(F)(F)F)nc3)[CH](c3ccccc3Cl)C2)c1
76	CS(=O)(=O)c1cc(N2CCN(Cc3ccc(C(F)(F)F)c(Cl)c3)C(c3ccccc3)C2)ccc1CO
77	CC1(C)CCC(C(=O)N2CCN(c3cccc(S(C)(=O)=O)c3)C[CH]2c2ccccc2)CC1
78	CS(=O)(=O)c1cccc(N2CCN(C(=O)C3CCC(C(F)F)CC3)[CH](c3ccccc3)C2)c1
79	CS(=O)(=O)c1cccc(N2CCN(C(=O)C3CCC(C(F)(F)F)CC3)[CH](c3ccccc3)C2)c1
80	CS(=O)(=O)c1cccc(N2CCN(C[CH]3CC[CH](C(F)(F)F)CC3)[CH](c3ccc(F)cc3)C2)c1
81	CS(=O)(=O)c1cccc(N2CCN(C[CH]3CCN(CC(F)(F)F)C3)[CH](c3ccccc3)C2)c1
82	CC(CCOc1cccc(CC(=O)O)c1)N(Cc1cccc(C(F)(F)F)c1Cl)CC(c1ccccc1)c1ccccc1
83	Fc1ccc(-c2c3cccc(C(F)(F)F)c3nn2Cc2ccc(F)cc2Cl)cc1
84	CCN(Cc1ccccc1)c1ccc(C(O)(C(F)(F)F)C(F)(F)F)cc1Cl
85	CCN(Cc1nc(-c2cccc(C(F)(F)F)c2)oc1C)c1ccc(C(O)(C(F)(F)F)C(F)(F)F)cc1Cl
86	CCN(c1ccc(C(O)(C(F)(F)F)C(F)(F)F)cc1)C(COC)c1ccccc1
87	CCN(c1ccc(C(O)(C(F)(F)F)C(F)(F)F)cc1)C(C)(C(=O)N(C)C)c1ccccc1
88	CCN(c1ccc(C(O)(C(F)(F)F)C(F)(F)F)cc1Cl)C(CO)c1ccccc1
89	CCN(c1ccc(C(O)(C(F)(F)F)C(F)(F)F)cc1)C(C)(C(=O)OC)c1ccccc1
90	CCN(Cc1ccccn1)c1ccc(C(O)(C(F)(F)F)C(F)(F)F)cc1
91	CCN(Cc1cscn1)c1ccc(C(O)(C(F)(F)F)C(F)(F)F)cc1Cl
92	OC(c1ccc(N(Cc2ccccc2)CC(F)(F)F)cc1)(C(F)(F)F)C(F)(F)F
93	CCN(Cc1nc(-c2cccc(Cl)c2)oc1C)c1ccc(C(O)(C(F)(F)F)C(F)(F)F)cc1
94	CCN(CCc1ccccc1)c1ccc(C(O)(C(F)(F)F)C(F)(F)F)cc1Cl
95	C[C]1(C(=O)NC(=O)C2(c3ccccc3)CCCCC2)CCC[C]2(C)c3ccccc3CC[CH]12
96	CC1(C(=O)NC(=O)[C]2(C)CCC[C]3(C)c4cc(O)ccc4CC[CH]23)CCC[C]2(C)c3cc(O)ccc3CC[CH]12
97	CCOC(=O)C12CC3CC(CC(NC(=O)[C]4(C)CCC[C]5(C)c6cc(O)ccc6CC[CH]45)(C3)C1)C2
98	C[C]1(C(=O)NCC23CC4CC(CC(C4)C2)C3)CCC[C]2(C)c3cc(O)ccc3CC[CH]12
99	C[C]1(C(=O)NC2C3CC4CC(C3)CC2C4)CCC[C]2(C)c3cc(O)ccc3CC[CH]12
100	CCCc1c(OCCCN(C)c2ccc(CC(=O)O)c(Cl)c2)ccc2c(C(F)(F)F)noc12
101	CCCc1c(OCCCn2ccc3cc(CC(=O)O)ccc32)ccc2c(C(F)(F)F)noc12
102	CCCc1c(OCCCN2C(=O)Cc3cc(CC(=O)O)ccc32)ccc2c(C(F)(F)F)noc12
103	CCCc1c(OCCCN(C)c2ccc(CC(=O)O)cc2)ccc2c(C(F)(F)F)noc12
104	O=C(O)Cc1ccc(COc2cccc(-c3c(Cc4ccccc4)cnc4c(C(F)(F)F)cccc34)c2)cc1
105	C=CCN(c1ccc(NC(=O)C(C)(O)C(F)(F)F)c(Cl)c1)S(=O)(=O)c1ccccc1Cl
106	CCCc1c(OCCCSc2ccc(CC(=O)O)cc2Cl)ccc2c(C(F)(F)F)noc12
107	CC1(C(=O)OC(=O)C2(C)CCC[C]3(C)c4cc(O)ccc4CC[CH]23)CCCC2(C)c3cc(O)ccc3CCC12
108	CC1(C(=O)CC(=O)[C]2(C)CCC[C]3(C)c4cc(O)ccc4CC[CH]23)CCC[C]2(C)c3cc(O)ccc3CC[CH]12
109	CS(=O)(=O)c1cccc(Oc2cccc(-c3c(-n4ccnc4)nc4c(C(F)(F)F)cccn34)c2)c1

110	CS(=O)(=O)c1cccc(Oc2cccc(-c3c(CN4CCCCC4)nc4c(C(F)(F)F)cccn34)c2)c1
111	CS(=O)(=O)c1cccc(Oc2cccc(-c3c(CN4CCSC4)nc4c(C(F)(F)F)cccn34)c2)c1
112	CS(=O)(=O)c1cccc(Oc2cccc(-c3c(-c4ccccc4)nc4c(C(F)(F)F)cccn34)c2)c1
113	CS(=O)(=O)c1cccc(Oc2cccc(-c3c(Cc4ccccc4)nc4c(C(F)(F)F)cccn34)c2)c1
114	CC(C)c1nc2c(C(F)(F)F)cccn2c1-c1cccc(Oc2cccc(S(C)(=O)=O)c2)c1
115	CC(C)(C)c1nc2c(C(F)(F)F)cccn2c1-c1cccc(Oc2cccc(S(C)(=O)=O)c2)c1
116	Cc1nc2c(C(F)(F)F)cccn2c1-c1cccc(Oc2cccc(S(=O)(=O)C(C)C)c2)c1
117	CC(C)(O)c1cn(-c2ccc(-c3cccc(S(C)(=O)=O)c3)cc2)c(Cc2ccccc2Cl)n1
118	CC(C)(O)c1cn(-c2ccc(-c3cccc(S(C)(=O)=O)c3)cc2)c(Cc2ccccc2C(F)(F)F)n1
119	CS(=O)(=O)c1cccc(-c2ccc(-n3cc(C(F)(F)F)nc3-c3ccccc3Cl)cc2)c1
120	COC(=O)c1cc(-c2ccc(-c3cccc(S(C)(=O)=O)c3)s2)n(-c2ccccc2Cl)n1
121	CS(=O)(=O)c1cccc(-c2ccc(-c3cc(C(F)(F)F)nn3-c3ccccc3Cl)cc2)c1
122	CS(=O)(=O)c1cccc(-c2ccc(-c3cc(C(F)(F)F)nn3-c3ccccc3C(F)(F)F)s2)c1
123	CS(=O)(=O)c1cccc(-c2ccc(-c3cc(C(F)(F)F)nn3-c3ccccc3Cl)s2)c1
124	CS(=O)(=O)c1cccc(-c2ccc(-c3cc(C(F)(F)F)nn3Cc3ccc(F)cc3F)s2)c1
125	Cc1cc(CC(=O)O)ccc1-c1ccc(-c2cc(C(F)(F)F)c(C#N)c(=O)n2Cc2ccc(F)cc2F)s1
126	N#Cc1c(C(F)(F)F)cc(-c2ccc(Oc3ccccc3)cc2)n(Cc2ccc(F)cc2F)c1=O
127	CS(=O)(=O)c1cccc(-c2ccc(-c3cc(C(F)(F)F)c(C#N)c(=O)n3Cc3ccc(F)cc3F)s2)c1
128	C=C[C]1(C)CC=C2[CH](CC[CH]3[C](C)(C(=O)O)CCC[C]23C)C1
129	C=C[C]1(C)CC=C2[CH](CC[CH]3[C](C)(CO)CCC[C]23C)C1
130	CC(C)=CCC[CH](C)[CH]1C[CH](O)[C]2(C)C3=CC[CH]4C(C)(C)[CH](O)CC[C]4(C)C3=CC[C]12C
131	C=C(CC[CH](C)[CH]1CC[CH]2[CH]3[CH](CC[C]21C)[C]1(C)CC[CH](O)C[C]1(O)[CH](O)[CH]3O)C(C)C
132	CC(C)[CH](C)[C]1(C)C[CH]1[CH](C)[CH]1CC[CH]2[CH]3CC=C4C[CH](O)CC[C]4(C)[C]3(O)[CH](O)C[C]21C
133	CC(C)[CH](C)[C]1(C)C[CH]1[CH](C)[CH]1CC[CH]2[CH]3C[CH](O)[C]4(O)C[CH](O)CC[C]4(C)[C]3(O)[CH](O)C[C]21C
134	CC(=O)Oc1ccc2c(c1)[C]1(C)CCC[C](C)(C(=O)OC(=O)[C]3(C)CCC[C]4(C)c5cc(OC(C)=O)ccc5CC[CH]34)[CH]1CC2
135	C[C]1(C(=O)NC23CC4CC(CC(C4)C2)C3)CCC[C]2(C)c3cc(O)ccc3CC[CH]12
136	CC(CNC(=O)[C]1(C)CCC[C]2(C)c3ccccc3CC[CH]12)(c1ccccc1)c1ccccc1
137	CC1(C)C2CCC(NC(=O)[C]3(C)CCC[C]4(C)c5cc(O)ccc5CC[CH]34)C1C2
138	CC(NC(=O)[C]1(C)CCC[C]2(C)c3cc(O)ccc3CC[CH]12)C12CC3CC(CC(C3)C1)C2
139	C[C]1(C(=O)NCC2(c3ccccc3)CCCCC2)CCC[C]2(C)c3cc(O)ccc3CC[CH]12
140	C[C]1(C(=O)N2CCC3(C=Cc4ccccc43)CC2)CCC[C]2(C)c3ccccc3CC[CH]12
141	C[C]1(C(=O)N2CCC(c3ccccc3)CC2)CCC[C]2(C)c3ccccc3CC[CH]12
142	C[C]1(C(=O)N2C3CCCCC3C3CCCCC32)CCC[C]2(C)c3cc(O)ccc3CC[CH]12
143	O=S(=O)(c1ccccc1)N1CCCCc2cc(C(O)(C(F)(F)F)C(F)(F)F)ccc21
144	CC(C)COC(=O)N(C)c1ccc(C(O)(C(F)(F)F)C(F)(F)F)cc1
145	O=S(=O)(c1ccccc1)N(c1ccc(C(O)(C(F)(F)F)C(F)(F)F)cc1)C1CCCC1
146	CN(C(=O)c1ccccc1)c1ccc(C(O)(C(F)(F)F)C(F)(F)F)cc1
147	CN(c1ccc(C(O)(C(F)(F)F)C(F)(F)F)cc1)S(=O)(=O)c1cccc(C#N)c1
148	CN(c1ccc(C(O)(C(F)(F)F)C(F)(F)F)cc1)S(=O)(=O)c1ccccc1C#N
149	CC(C)CN(c1ccc(C(O)(C(F)(F)F)C(F)(F)F)cc1)S(=O)(=O)c1cccs1
150	CN(c1ccc(C(O)(C(F)(F)F)C(F)(F)F)cc1)S(=O)(=O)c1cccc(S(N)(=O)=O)c1
151	CN(c1ccc(C(O)(C(F)(F)F)C(F)(F)F)cc1)S(=O)(=O)c1cccc(-c2ccccc2)c1
152	O=S(=O)(c1ccccc1)N1CCOc2cc(C(O)(C(F)(F)F)C(F)(F)F)ccc21
153	CN(c1ccc(C(O)(C(F)(F)F)C(F)(F)F)cc1)S(=O)(=O)c1cccs1
154	CN(c1ccc(C(O)(C(F)(F)F)C(F)(F)F)cc1Cl)S(=O)(=O)c1ccccc1
155	CN(c1ccccc1)S(=O)(=O)c1ccc(C(O)(C(F)(F)F)C(F)(F)F)cc1

156	CCCc1c(OCCCn2ncc3cc(CC(=O)O)ccc32)ccc2c(C(F)(F)F)noc12
157	CCCc1c(OCCCN(C)c2cnc(CC(=O)O)cn2)ccc2c(C(F)(F)F)noc12
158	CCCc1cc(C(O)(C(F)(F)F)C(F)(F)F)cc(CCC)c1OCCCCN1C(=O)N(C)C(C)(C)C1=O
159	CCCc1cc(C(O)(C(F)(F)F)C(F)(F)F)cc(CCC)c1OCCCCN1C(=O)NC(C)(c2ccc3c(c2)OCO3)C1=O
160	CCCc1cc(C(O)(C(F)(F)F)C(F)(F)F)cc(CCC)c1OCCCCN1C(=O)NC(C)(c2ccc(OCC)cc2)C1=O
161	CCN(c1ccc(C(O)(C(F)(F)F)C(F)(F)F)cc1)C(COCC(=O)N(C)C)c1ccccc1
162	OC(c1ccc(N(Cc2ccccc2)CC(F)(F)F)c(Cl)c1)(C(F)(F)F)C(F)(F)F
163	COC(=O)C1(c2ccccc2)CCCCN1c1ccc(C(O)(C(F)(F)F)C(F)(F)F)cc1
164	CCN(c1ccc(C(O)(C(F)(F)F)C(F)(F)F)cc1)[CH](CO)c1ccccc1
165	CCN(c1ccc(C(O)(C(F)(F)F)C(F)(F)F)cc1Cl)C(CC)(C(=O)OC)c1ccccc1
166	CCN(c1ccc(C(O)(C(F)(F)F)C(F)(F)F)cc1Cl)C(COc1ccc(C(=O)OC)cc1)c1ccccc1
167	CCN(c1ccc(C(O)(C(F)(F)F)C(F)(F)F)cc1)C(CC)(C(=O)OC)c1ccccc1
168	CC(C)(C)OC(=O)c1c(COc2ccc(-c3ccc(CC(=O)O)cc3)cc2)ccc(C(F)(F)F)c1O
169	CC(=O)N(C)c1ccc(OCc2ccc(C(F)(F)F)c(O)c2C(=O)OC(C)(C)C)cc1
170	CC(=O)N(C)c1ccc(OCc2ccc(C(F)(F)F)cc2C(=O)OC(C)(C)C)cc1
171	O=C(O)c1cccc(OCCCN(Cc2cccc(C(F)(F)F)c2Cl)CC(c2ccccc2)c2ccccc2)c1
172	O=C(O)Cc1cccc2c(NCc3cccc(-c4c(Cc5ccccc5)cnc5c(C(F)(F)F)cccc45)c3)cccc12
173	CC(C)[C](O)(C(=O)N1CCC(C2CCN(c3ccc(C(=O)N(C)C)c(Cl)c3)CC2)CC1)C(F)(F)F
174	CN(C)C(=O)c1ccc(-c2cnc(N3CCN(C(=O)[C](O)(c4ccccc4)C(F)(F)F)CC3)c(C#N)c2)cc1Cl
175	CN(C)C(=O)c1ccc(-c2cnc(N3CCN(C(=O)OC(C)(C)C)CC3)c(C#N)c2)cc1Cl
176	CN(C)C(=O)c1ccc(N2CCC(C3CCN(C(=O)[C](O)(c4ccccc4)C(F)(F)F)CC3)CC2)cc1Cl
177	CS(=O)(=O)c1cc(-c2ccc(CN(Cc3ccccc3)S(=O)(=O)c3ccccc3C(F)(F)F)s2)ccc1CO
178	CC(C)[CH]1CN(c2ccc(CO)c(S(C)(=O)=O)c2)CCN1c1ncc(CO)c(C(F)(F)F)n1
179	CC(C)[CH]1CN(c2ccc(F)c(S(C)(=O)=O)c2)CCN1c1ncc(Cl)c(C(F)(F)F)n1
180	CCCc1cc(C(O)(C(F)(F)F)C(F)(F)F)cc(CCC)c1OCC#CCN1C(=O)NC(C)(c2ccc(OC(C)C)cc2)C1=O
181	CCCc1cc(C(O)(C(F)(F)F)C(F)(F)F)ccc1Oc1ccc(C(=O)CN2C(=O)NC(C)(c3ccc(OC(C)C)cn3)C2=O)c(O)c1
182	COc1cc(N2Cc3cn(-c4cccc(S(C)(=O)=O)c4)nc3[CH]2C(C)C)cc(C)n1
183	CC(C)[CH]1c2nn(-c3cccc(S(C)(=O)=O)c3)cc2CN1c1ncc(CO)c(C(F)(F)F)n1
184	CC(C)[CH]1c2nn(-c3cccc(S(C)(=O)=O)c3)cc2CN1c1nccc(C(F)(F)F)n1
185	CC(C)[CH]1c2nn(-c3cccc(S(C)(=O)=O)c3)cc2CN1c1cc(C(F)(F)F)ncn1
186	COc1cc(N2Cc3cn(-c4cccc(S(C)(=O)=O)c4)nc3[CH]2C(C)C)ccn1
187	Cc1nc(N2Cc3cn(-c4cccc(S(C)(=O)=O)c4)nc3[CH]2C(C)C)cc(C(F)(F)F)n1
188	COc1cc(N2Cc3cn(-c4cccc(S(C)(=O)=O)c4)nc3[CH]2C(C)(C)C)ccn1
189	COc1ncnc(N2Cc3cn(-c4cccc(S(C)(=O)=O)c4)nc3[CH]2C(C)C)c1F
190	CC(C)[CH]1c2nn(-c3ccc(CO)c(S(C)(=O)=O)c3)cc2CN1c1ncc(CO)c(C(F)(F)F)n1
191	CC(C)[CH]1c2nn(-c3cccc(S(C)(=O)=O)c3)cc2CN1c1ccnc(C(F)(F)F)n1
192	COc1cc(N2Cc3cn(-c4cccc(S(C)(=O)=O)c4)nc3[CH]2C(C)C)nc(C)n1
193	COc1ncc(F)c(N2Cc3cn(-c4cccc(S(C)(=O)=O)c4)nc3[CH]2C(C)C)n1
194	COc1nc(C)cc(N2Cc3cn(-c4cccc(S(C)(=O)=O)c4)nc3[CH]2C(C)C)n1
195	CC(C)(O)c1cn(-c2ccc(-c3cc(F)c(CO)c(S(C)(=O)=O)c3)cc2F)c(C(C)(C)c2c(Cl)cccc2Cl)n1
196	CC(O)c1ccc(-c2ccc(-n3cc(C(C)(C)O)nc3C(C)(C)c3ccccc3Cl)cc2)cc1S(C)(=O)=O
197	CC(c1c(Cl)cccc1Cl)c1nc(C(C)(C)O)cn1-c1ccc(-c2cc(F)c(CO)c(S(C)(=O)=O)c2)cc1F
198	CC(C)(O)c1cn(-c2ccc(-c3cccc(S(C)(=O)=O)c3)cc2)c(C(C)(C)c2c(Cl)cccc2Cl)n1
199	CC(C)(O)c1cn(-c2ccc(-c3cc(F)c(CO)c(S(C)(=O)=O)c3)cc2)c(C(C)(C)c2cccc(F)c2Cl)n1
200	CC(C)(O)c1cn(-c2ccc(-c3cc(F)c(CO)c(S(C)(=O)=O)c3)cc2)c(Cc2c(Cl)cccc2Cl)n1
201	CC(C)(O)c1cn(-c2ccc(-c3ccc(CO)c(S(C)(=O)=O)c3)cc2)c(Cc2ccccc2Cl)n1

202	CC(C)(O)c1cn(-c2ccc(-c3ccc(CO)c(S(C)(=O)=O)c3)cc2)c(C(C)(C)c2ccccc2Cl)n1
203	O=C(O)C1CCCc2c(OCCCN(Cc3cccc(C(F)(F)F)c3Cl)CC(c3ccccc3)c3ccccc3)cccc21
204	C[CH](CCOc1cccc(C(C)(C)C(=O)O)c1)N(Cc1cccc(C(F)(F)F)c1Cl)CC(c1ccccc1)c1ccccc1
205	CC(C(=O)O)c1cccc(OCCCN(Cc2cccc(C(F)(F)F)c2Cl)CC(c2ccccc2)c2ccccc2)c1
206	CC(C)(C(=O)O)c1cccc(OCCCN(Cc2cccc(C(F)(F)F)c2Cl)CC(c2ccccc2)c2ccccc2)c1
207	O=C(O)Cc1ccc2oc(CCN(Cc3cccc(C(F)(F)F)c3Cl)CC(c3ccccc3)c3ccccc3)cc2c1
208	C[CH](CCN(Cc1cccc(C(F)(F)F)c1Cl)CC(c1ccccc1)c1ccccc1)Oc1cccc(CC(=O)O)c1
209	C[CH](COc1cccc(CC(=O)O)c1)CN(Cc1cccc(C(F)(F)F)c1Cl)CC(c1ccccc1)c1ccccc1
210	C=C(c1ccc(C(=O)O)cc1)c1cc2c(cc1C)C(C)(C)CCC2(C)C
211	COc1ccc(CN(C)S(=O)(=O)c2cccc(Oc3cccc(-c4c(C(C)C)nc5c(Cl)cccn45)c3)c2)cc1
212	CNS(=O)(=O)c1cccc(Oc2cccc(-c3c(C(C)C)nc4c(Cl)cccn34)c2)c1
213	Cc1cnc2c(C(F)(F)F)cccc2c1-c1cccc(Oc2cccc(S(C)(=O)=O)c2)c1
214	Cc1nc2c(C(F)(F)F)cccc2n1-c1cccc(Oc2cccc(S(C)(=O)=O)c2)c1
215	CCS(=O)(=O)c1cccc(Oc2cccc(-c3c(C)nc4c(C(F)(F)F)cccn34)c2)c1
216	CN1C(=O)c2ccccc2CCc2cc(C(O)(C(F)(F)F)C(F)(F)F)ccc21
217	CS(=O)(=O)c1cccc(-c2ccc(CN(Cc3ccc(F)cc3Cl)S(=O)(=O)c3ccccc3)cc2)c1
218	CS(=O)(=O)c1cccc(-c2ccc(CN(Cc3c(F)cccc3Cl)S(=O)(=O)c3ccccc3)cc2)c1
219	CS(=O)(=O)c1cccc(-c2ccc(CN(Cc3c(F)cccc3Cl)S(=O)(=O)C3CC3)cc2)c1
220	Cc1cc(C)c(S(=O)(=O)N(Cc2ccc(-c3cccc(S(C)(=O)=O)c3)cc2)Cc2c(F)cccc2Cl)c(C)c1
221	Cc1cc(C)c(S(=O)(=O)N(Cc2ccc(-c3cccc(S(C)(=O)=O)c3)cc2)Cc2ccc(F)cc2Cl)c(C)c1
222	Cn1cnc(S(=O)(=O)N(Cc2ccc(-c3cccc(S(C)(=O)=O)c3)cc2)Cc2c(F)cccc2Cl)c1
223	CCN(c1ccc(C(O)(C(F)(F)F)C(F)(F)F)cc1)S(=O)(=O)c1ccccc1
224	NC(=O)CC1CN(S(=O)(=O)c2ccc(F)cc2)c2ccc(C(O)(C(F)(F)F)C(F)(F)F)cc2S1
225	CC(C)(O)CNC(=O)CC1CSc2cc(C(O)(C(F)(F)F)C(F)(F)F)ccc2N1S(=O)(=O)c1ccc(F)cc1
226	CC1CCN(S(=O)(=O)c2ccc(F)cc2)c2ccc(C(O)(C(F)(F)F)C(F)(F)F)cc21
227	CC1(C)CCN(S(=O)(=O)c2ccc(F)cc2)c2ccc(C(O)(C(F)(F)F)C(F)(F)F)cc21
228	O=C(CC1CN(S(=O)(=O)c2ccc(F)cc2)c2ccc(C(O)(C(F)(F)F)C(F)(F)F)cc2S1)Nc1nncs1
229	O=C(CC1CN(S(=O)(=O)c2ccc(F)cc2)c2ccc(C(O)(C(F)(F)F)C(F)(F)F)cc2S1)N1CCOCC1
230	O=S(=O)(c1ccc(F)cc1)N1CCSc2cc(C(O)(C(F)(F)F)C(F)(F)F)ccc21
231	COC(=O)CC1CN(S(=O)(=O)c2ccc(F)cc2)c2ccc(C(O)(C(F)(F)F)C(F)(F)F)cc2S1
232	O=S(=O)(c1ccc(F)cc1)N1CCNc2cc(C(O)(C(F)(F)F)C(F)(F)F)ccc21
233	CC(C)(O)CC(=O)NCC1CN(S(=O)(=O)c2ccc(F)cc2)c2ccc(C(O)(C(F)(F)F)C(F)(F)F)cc2S1
234	CC(C)(O)CNC(=O)C[CH]1CCc2cc(C(O)(C(F)(F)F)C(F)(F)F)ccc2N1S(=O)(=O)c1ccc(F)cc1
235	CN1CCN(S(=O)(=O)c2ccc(F)cc2)c2ccc(C(O)(C(F)(F)F)C(F)(F)F)cc21
236	O=C1c2c(Cl)c(Cl)c(Cl)c(Cl)c2C(=O)N1c1ccccc1C=Cc1cccs1
237	CN(CC(=O)Nc1ccc(F)c(F)c1F)C(=O)c1ccc2c(c1)OCCO2
238	COC1CCN(C(=O)CC[CH](C)[CH]2CC[CH]3[CH]4CC=C5C[CH](O)CC[C]5(C)[CH]4CC[C]32C)CC1
239	COC(=O)N1CCC(c2ccc(C(O)(C(F)(F)F)C(F)(F)F)cc2)(S(=O)(=O)c2ccc(F)cc2)C1
240	c1ccc(-c2n[nH]c(-c3ccccc3)n2)cc1
241	CC(=O)n1nc(Cc2ccccc2)nc1-c1ccccc1
242	CC(=O)n1nc(-c2ccc(Br)cc2)nc1-c1ccccc1
243	Clc1ccccc1-c1n[nH]c(Cc2ccccc2)n1
244	COc1cccc(-c2n[nH]c(-c3ccccc3)n2)c1
245	Brc1ccc(-c2n[nH]c(-c3ccccc3)n2)cc1
246	Clc1ccc(-c2n[nH]c(-c3ccccc3)n2)cc1
247	Brc1ccccc1-c1n[nH]c(-c2ccccc2)n1

248	COc1cccc(-c2nc(-c3ccccc3)n(C(C)=O)n2)c1
249	COc1cccc(-c2nc(-c3ccccc3)n(Cc3ccccc3)n2)c1
250	Clc1ccccc1-c1n[nH]c(-c2ccccc2)n1
251	Cc1ccc(-c2n[nH]c(-c3ccccc3)n2)cc1
252	CC(=O)n1nc(-c2ccc(C)cc2)nc1-c1ccccc1

Table S4. Fine-tuning set II.

ID	SMILES
1	ClC1=C(C(N(C)C)=O)C=CC(N2CCC(C3CCN(C([C@@](O)(C(F)(F)F)C(C)C)=O)CC3)CC2)=C1
2	OCC1=C(F)C=C(C2=CC=C(N3C(C(C4=C(Cl)C=CC=C4Cl)(C)C)=NC(C(C)(O)C)=C3)C(F)=C2)C=C1S(C)(=O)=O
3	OCC1=CC=C(N2CCN(C3=NC(C(F)(F)F)=C(CO)C=N3)[C@H](C(C)C)C2)C=C1S(C)(=O)=O
4	O=C(O)CC1=CC(OCCCN(CC2=C(Cl)C(C(F)(F)F)=CC=C2)CC(C3=CC=CC=C3)C4=CC=CC=C4)=CC=C1
5	OC(C(F)(F)F)(C(F)(F)F)C1=CC=C(N(CC(F)(F)F)S(=O)(C2=CC=CC=C2)=O)C=C1
6	FC(C=C1)=CC=C1C2=C3C=CC=C(C(F)(F)F)C3=NN2CC4=CC=C(F)C=C4Cl
7	CS(C1=CC(C2=CC=C(C3=C(C)N=C(C(C(F)(F)F)=CC=C4)C4=N3)C=C2)=CC=C1)(=O)=O
8	ClC(C=C1)=CC=C1C2=NN(C(C3=C(F)C=C(F)C=C3F)=O)[C@@H](C4=C(OC)C(OC)=CC=C4)S2
9	O=C(O)CC(C=C1)=CC=C1COC2=CC=CC(C3=C4C(C(C(F)(F)F)=CC=C4)=NC=C3CC5=CC=CC=C5)=C2
10	ClC1=C2C(C(N(C3=CC=CC=C3/C=C/C4=CC=CC=C4)C2=O)=O)=C(Cl)C(Cl)=C1Cl
11	CN1C=CC2=CC=CC(OC3=CC=CC(C4=C5C(C(C(F)(F)F)=CC=C5)=NN=C4CC6=CC=CC=C6)=C3)=C21
12	CCCC1=C(OC2=CC(O)=C(C(CN3C(NC(C)(C4=NC=C(OC(C)C)C=C4)C3=O)=O)=O)C=C2)C=CC(C(O)(C(F)(F)F)C(F)(F)F)=C1

3. NMR Spectra & HPLC Chromatograms

2-(4-[Ethylamino]phenyl)-1,1,1,3,3,3-hexafluoropropan-2-ol (6)

^{1}H NMR (400 MHz, $CDCl_3$)

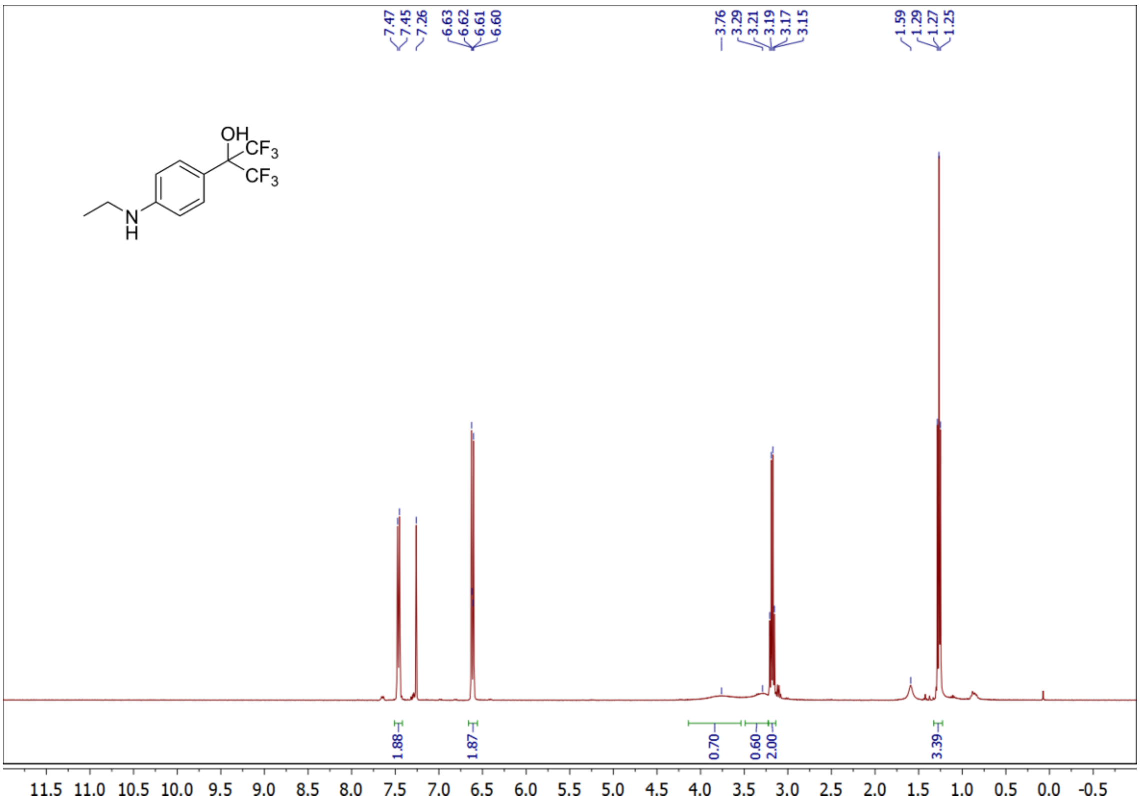

^{13}C NMR (101 MHz, $CDCl_3$)

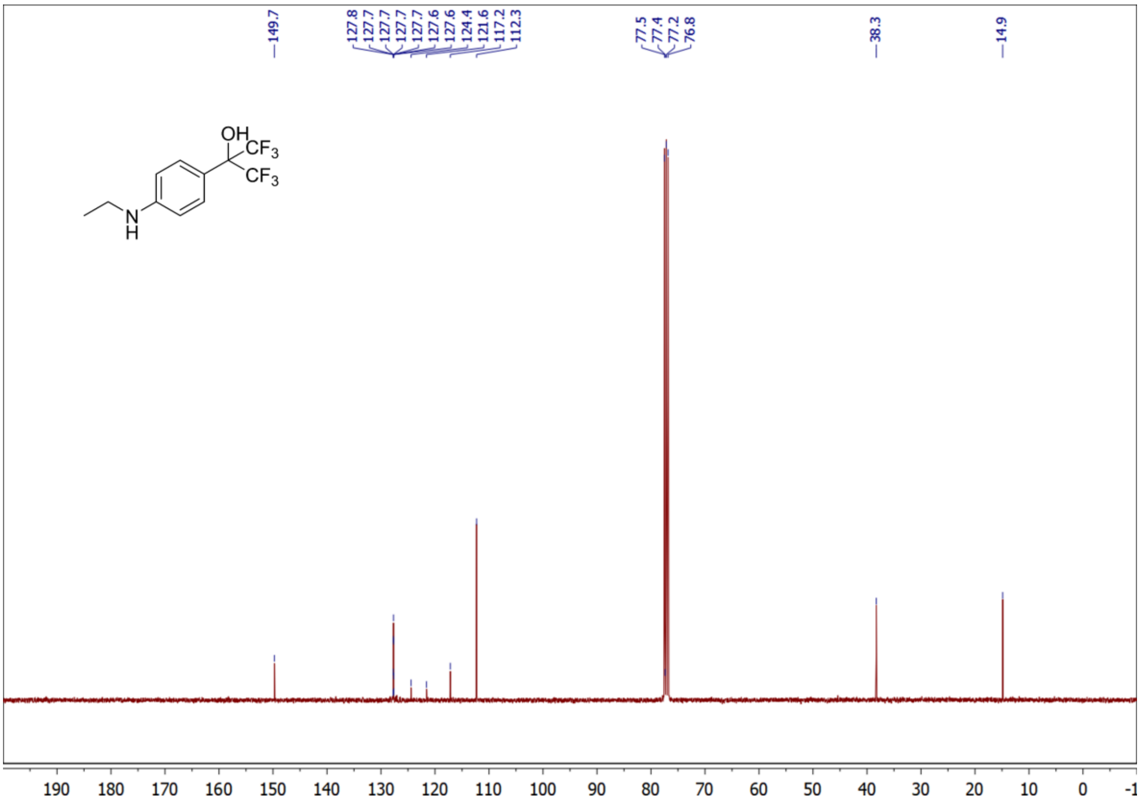

^{19}F-NMR (377 MHz, $CDCl_3$)

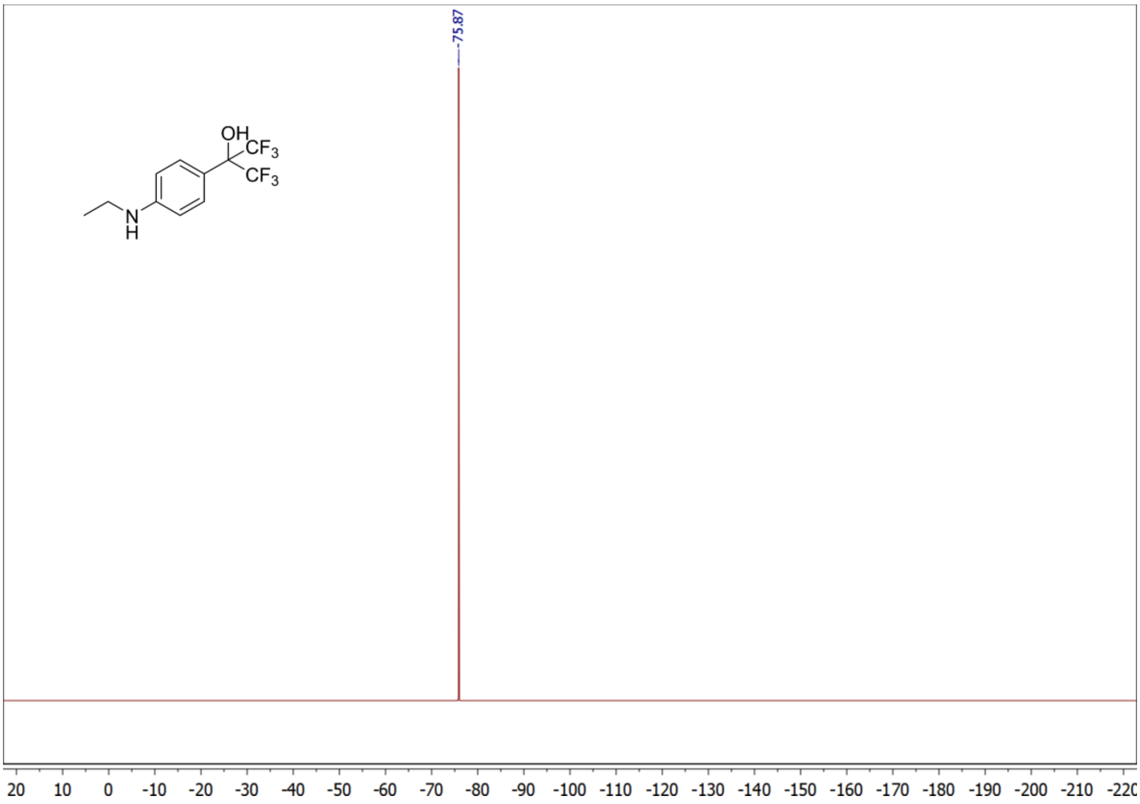

N-Ethyl-N-(4-[1,1,1,3,3,3-hexafluoro-2-hydroxypropan-2-yl]phenyl)benzamide (1)

^{1}H NMR (600 MHz, $CDCl_3$)

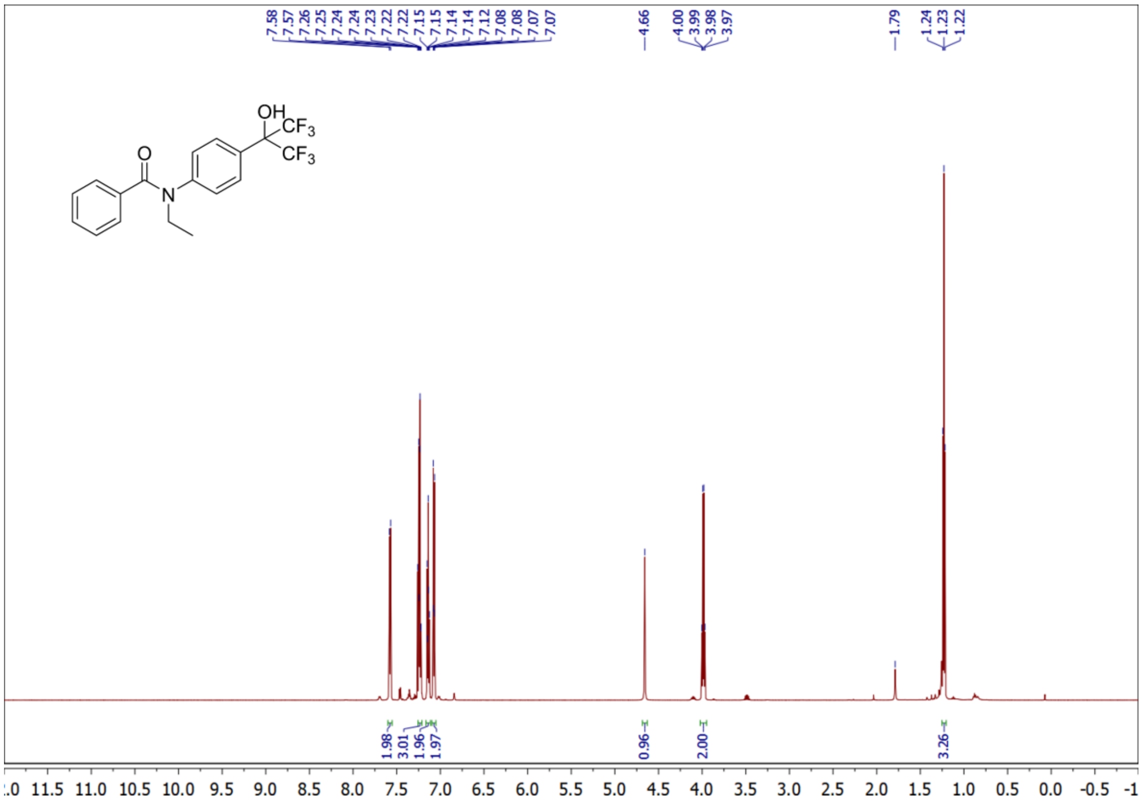

^{13}C NMR (151 MHz, $CDCl_3$)

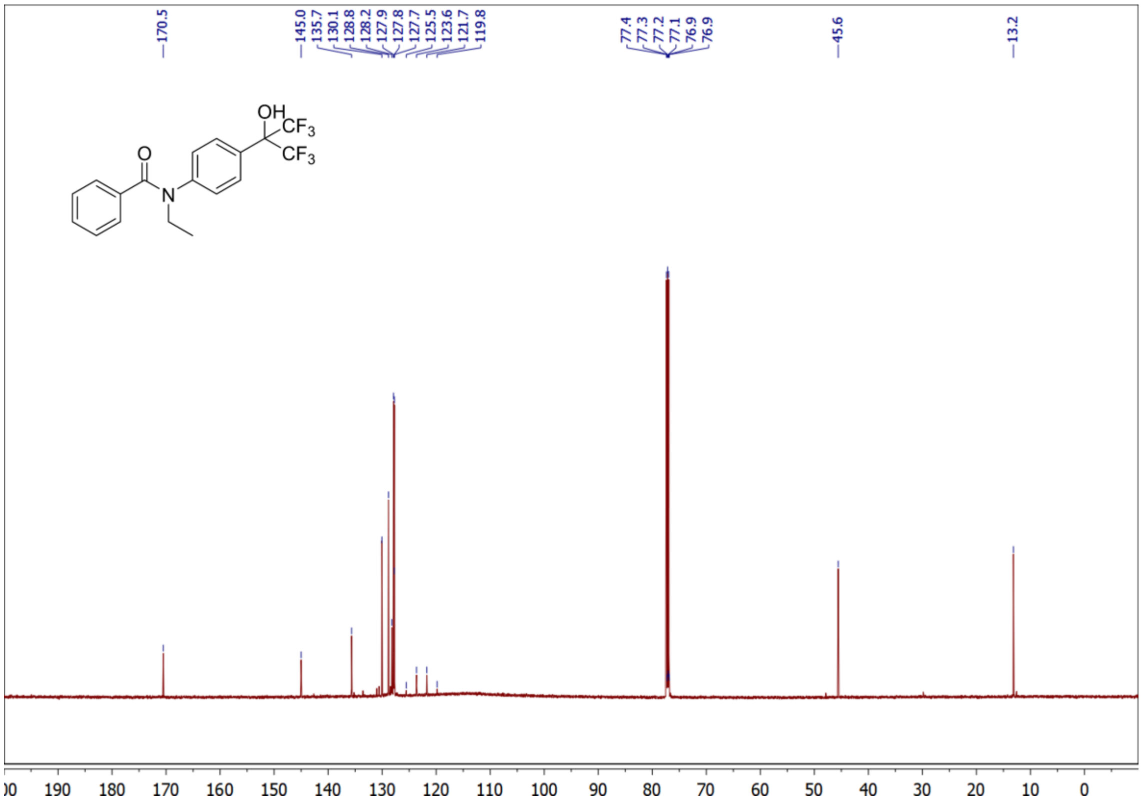

^{19}F-NMR (282 MHz, $CDCl_3$)

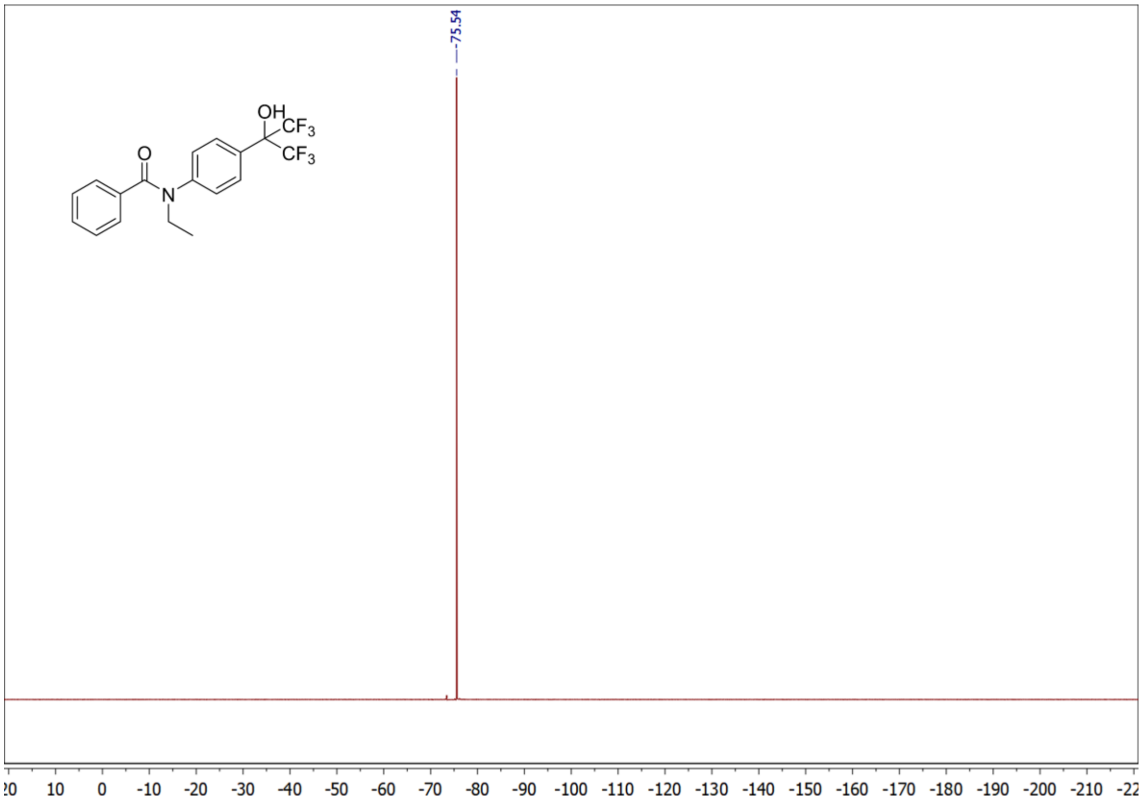

HPLC Traces

Chrom Type: Fixed WL Chromatogram, 254 nm

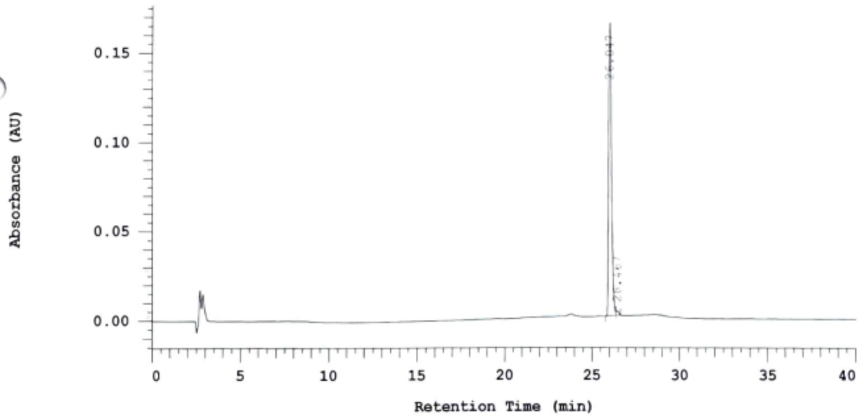

No.	RT	Area	Area %	Height
1	26.047	918370	98.632	82690
2	26.467	12742	1.368	1214
		931112	100.000	83904

Peak rejection level: 0

Chrom Type: Fixed WL Chromatogram, 280 nm

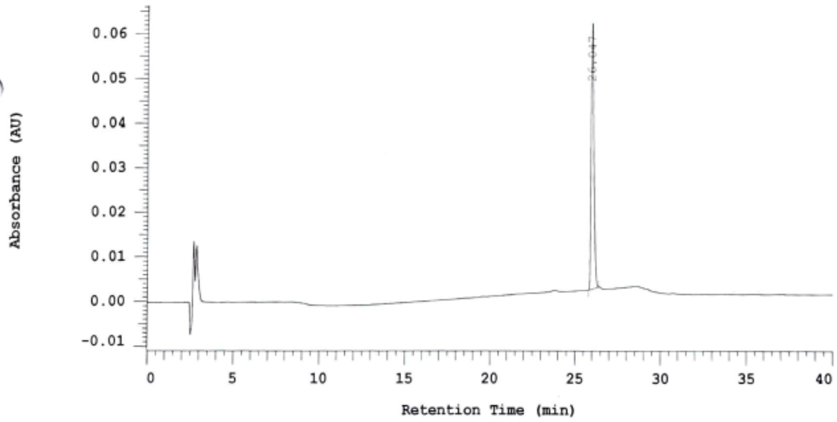

No.	RT	Area	Area %	Height
1	26.047	329164	100.000	29904
		329164	100.000	29904

Peak rejection level: 0

***tert*-Butyl 4-(3-[methylsulfonyl]phenyl)piperazine-1-carboxylate (10)**

^{1}H NMR (300 MHz, $CDCl_3$)

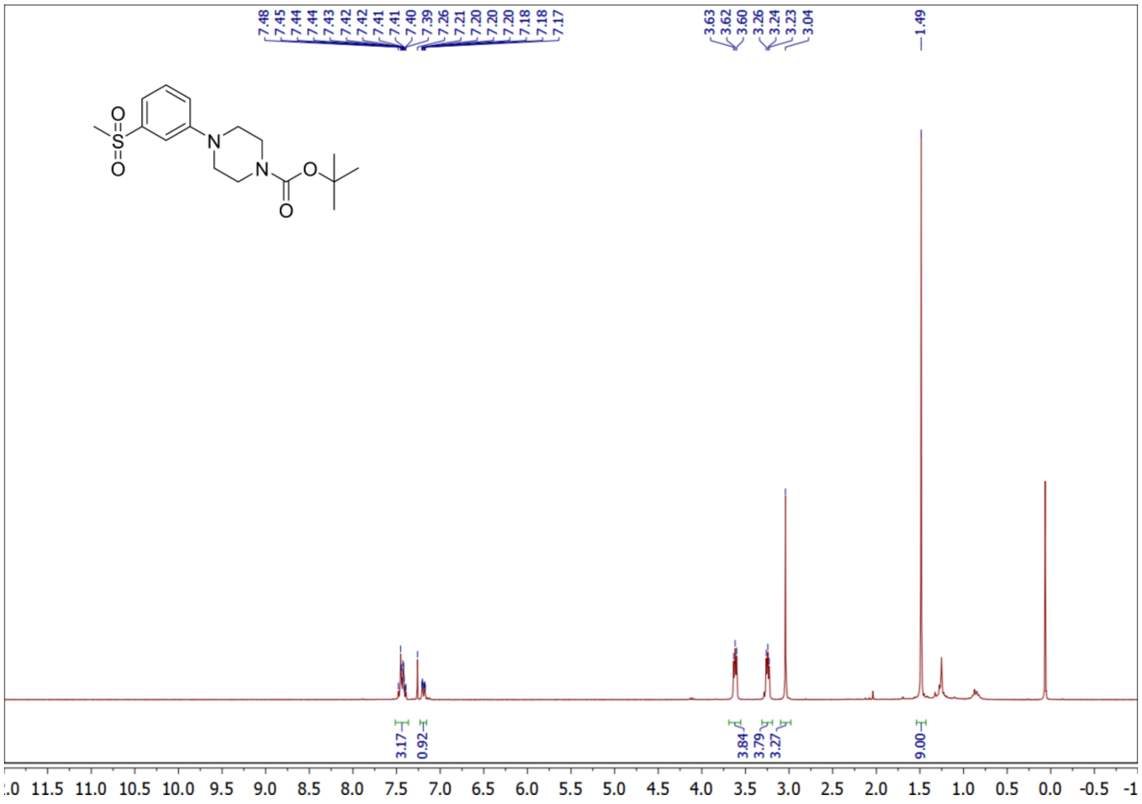

^{13}C NMR (75 MHz, $CDCl_3$)

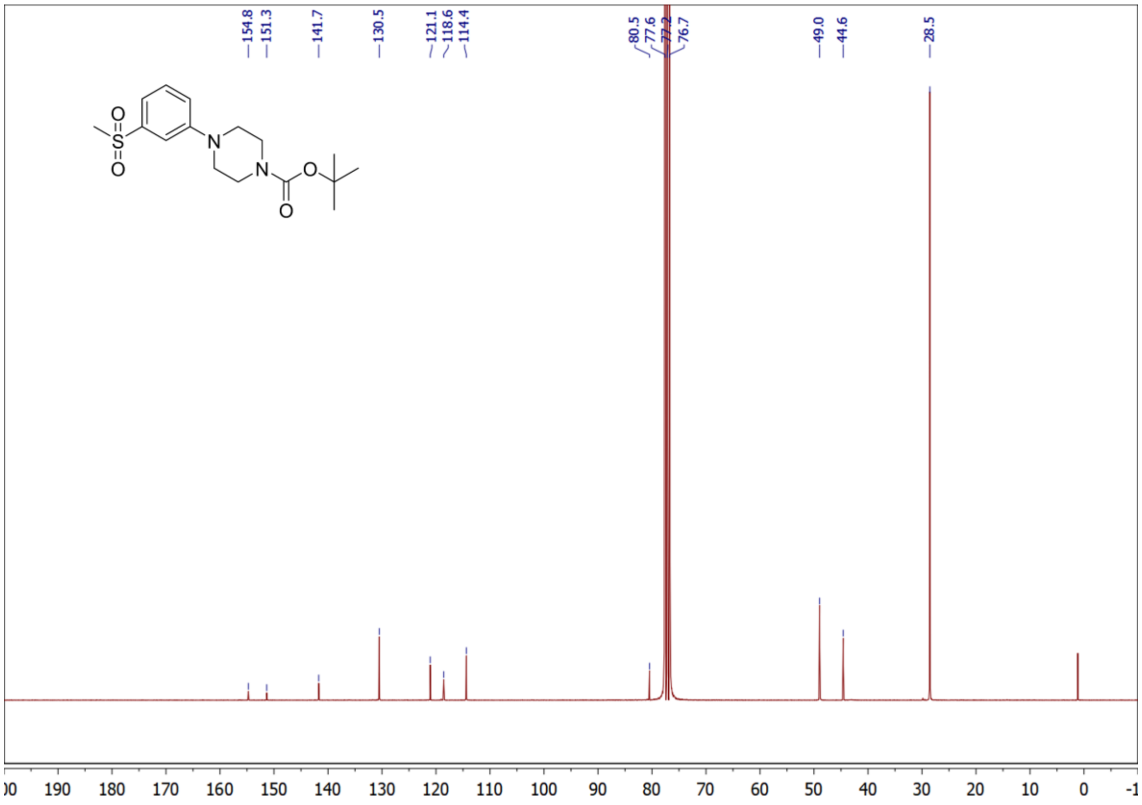

4-(3-[Methylsulfonyl]phenyl)piperazin-1-ium trifluoroacetate (11)

^{1}H NMR (300 MHz, DMSO-d_6)

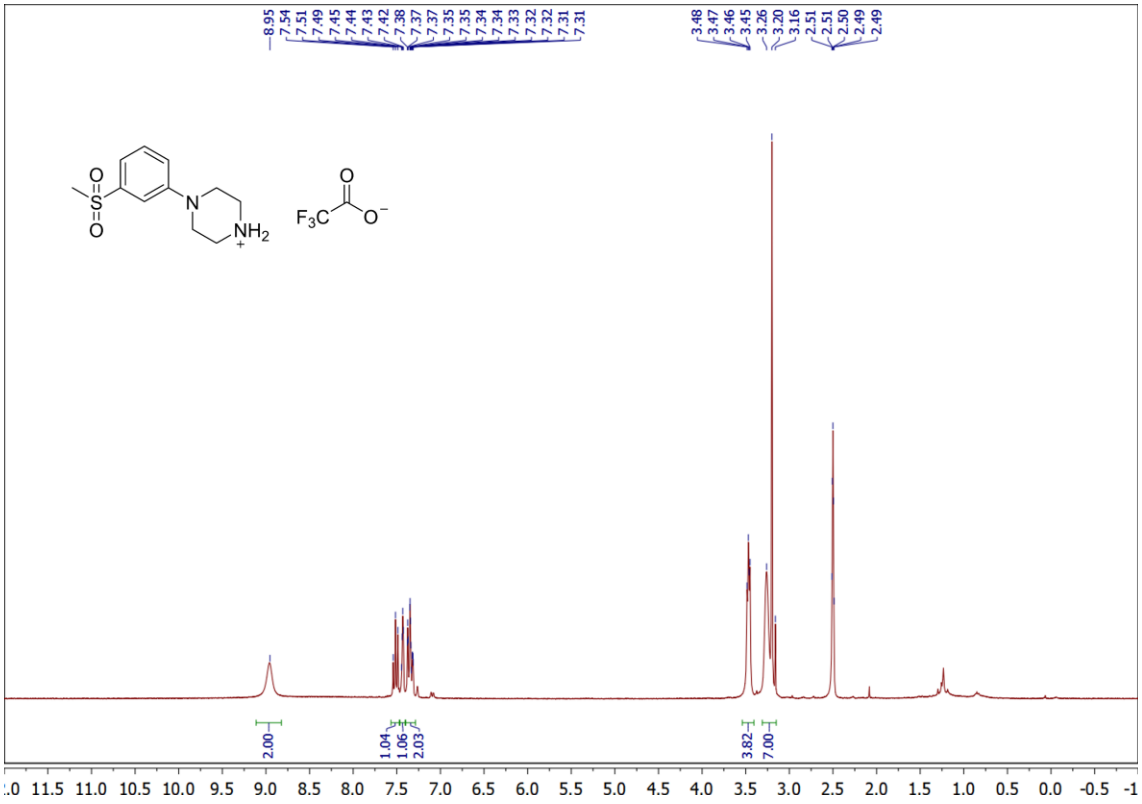

^{13}C NMR (75 MHz, DMSO-d_6)

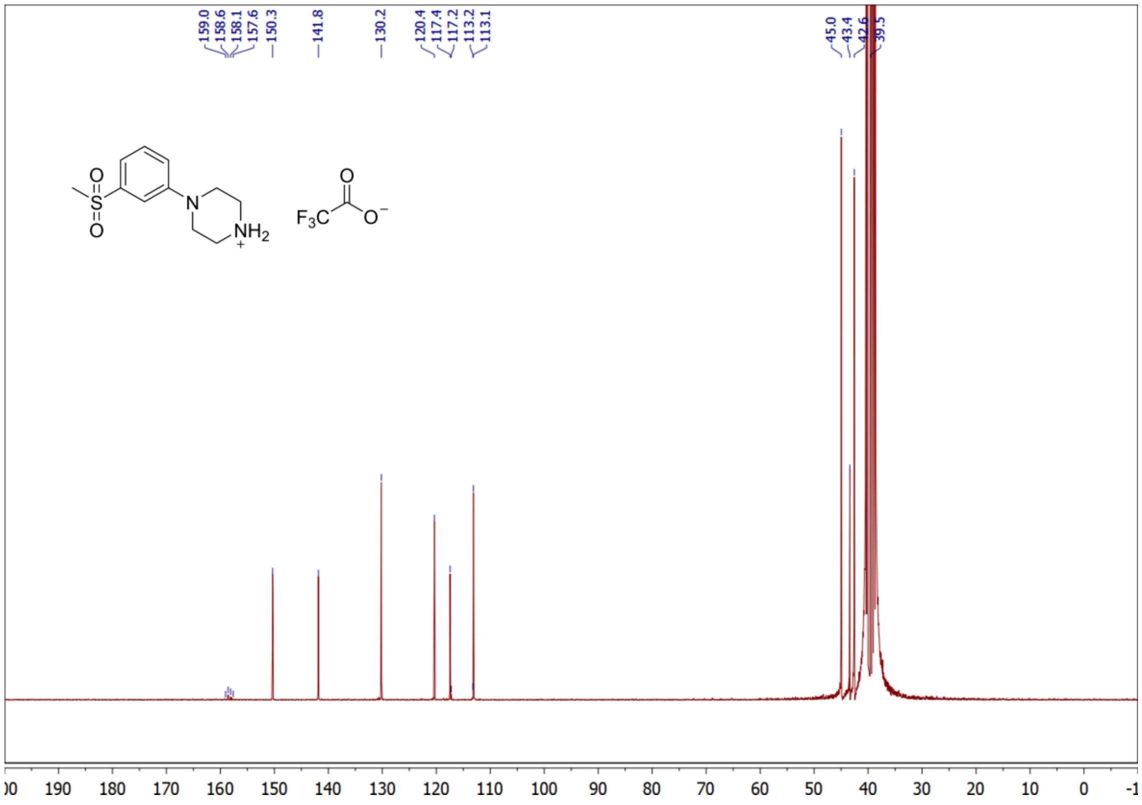

1-(4-Chloro-3-[trifluoromethyl]phenyl)-4-(3-[methylsulfonyl]phenyl)piperazine (2)

^{1}H NMR (500 MHz, $CDCl_3$)

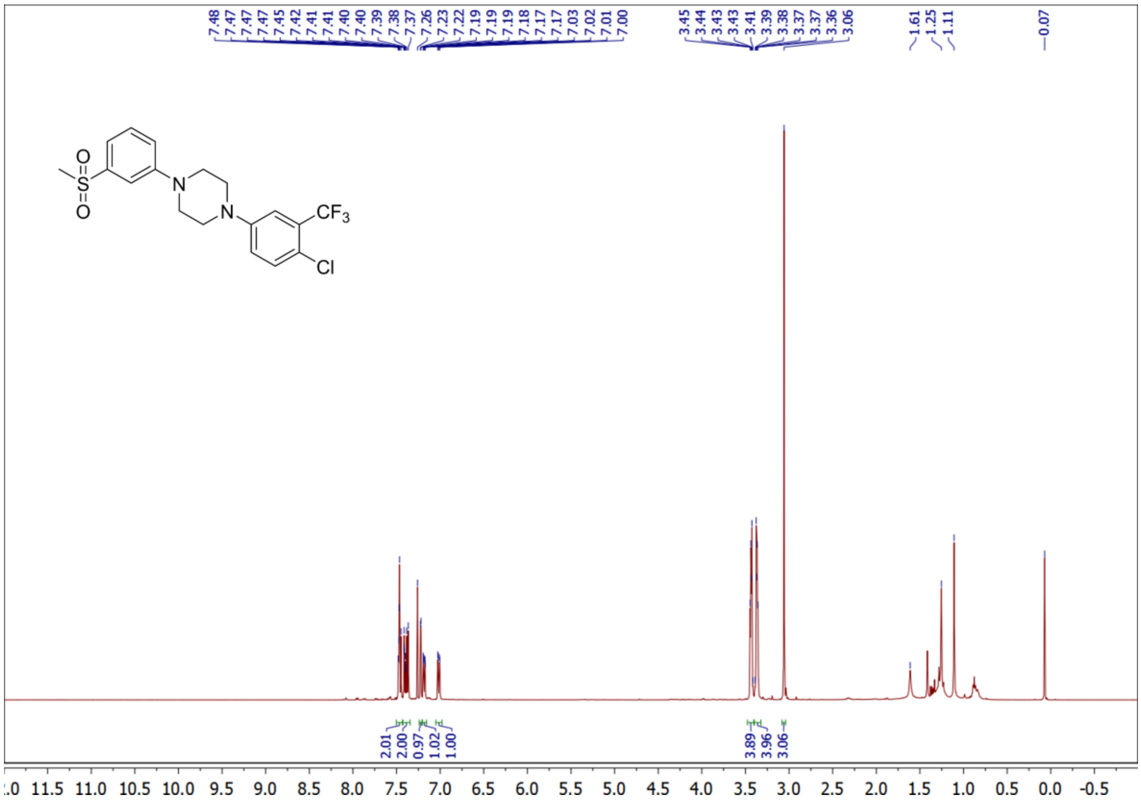

^{13}C NMR (126 MHz, $CDCl_3$)

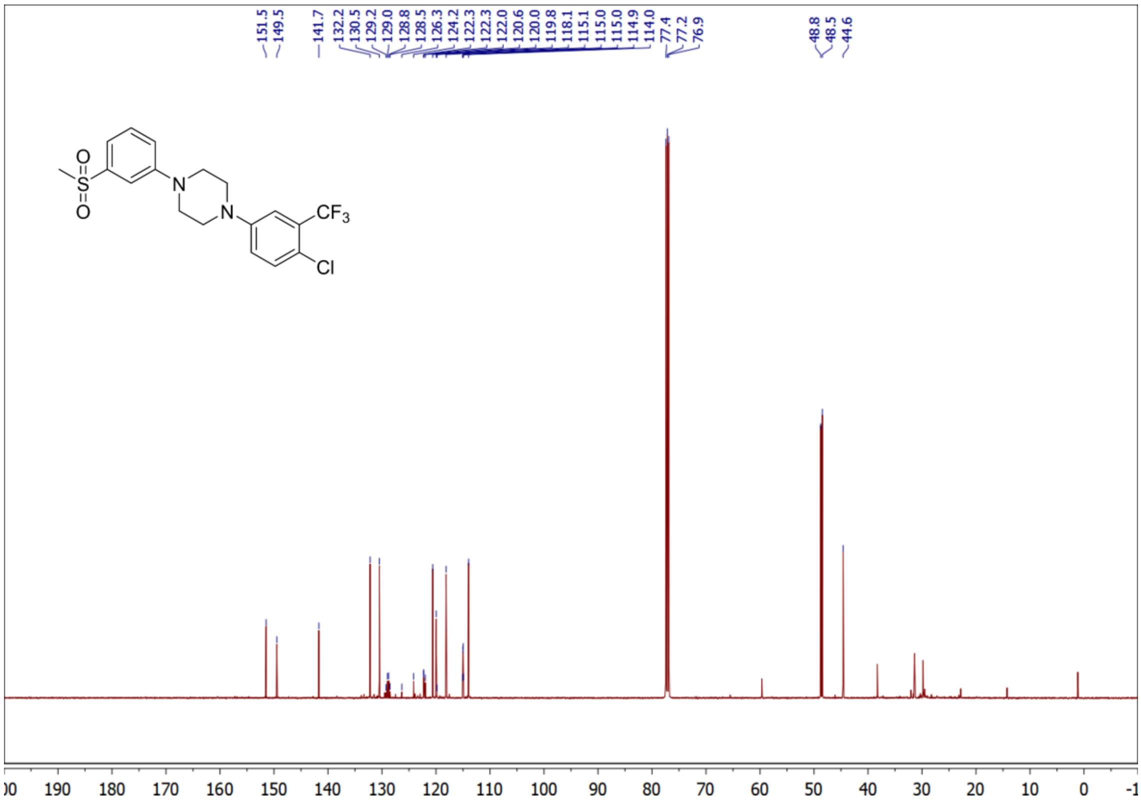

^{19}F-NMR (471 MHz, $CDCl_3$)

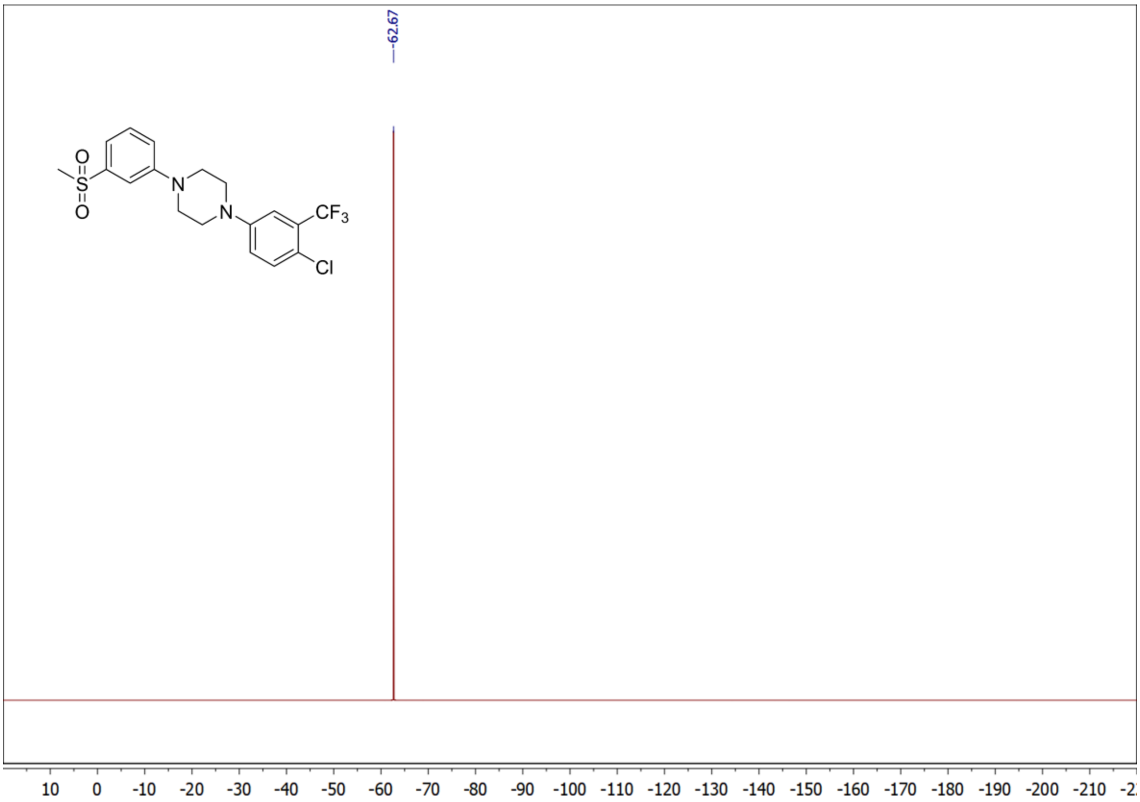

HPLC Traces

Chrom Type: Fixed WL Chromatogram, 254 nm

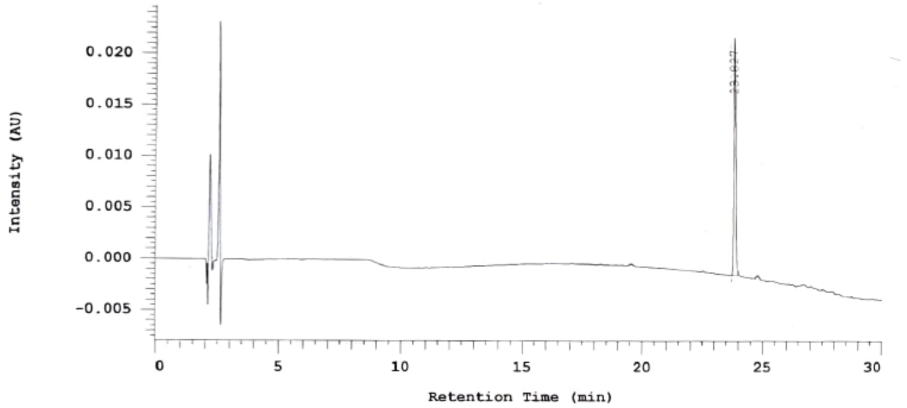

No.	RT	Area	Area %	Height
1	23.827	61247	100.000	11707
		61247	100.000	11707

Peak rejection level: 0

Chrom Type: Fixed WL Chromatogram, 280 nm

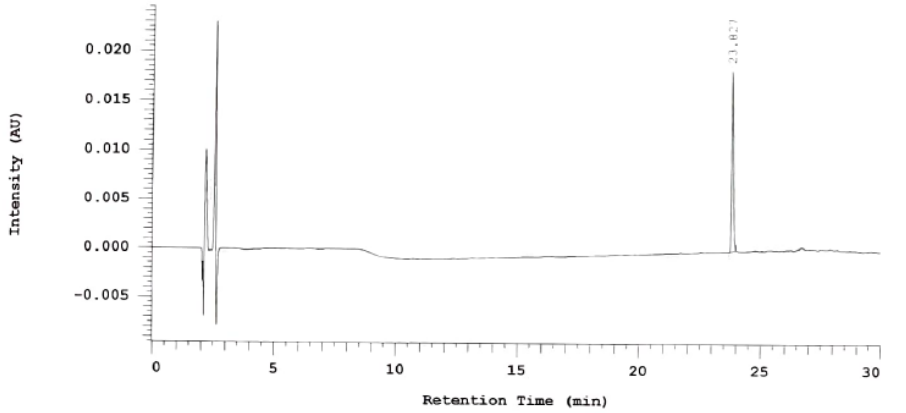

No.	RT	Area	Area %	Height
1	23.827	47099	100.000	9049
		47099	100.000	9049

Peak rejection level: 0

(3'-[Methylsulfonyl]-[1,1'-biphenyl]-4-yl)methanol (14)

^{1}H NMR (300 MHz, $CDCl_3$)

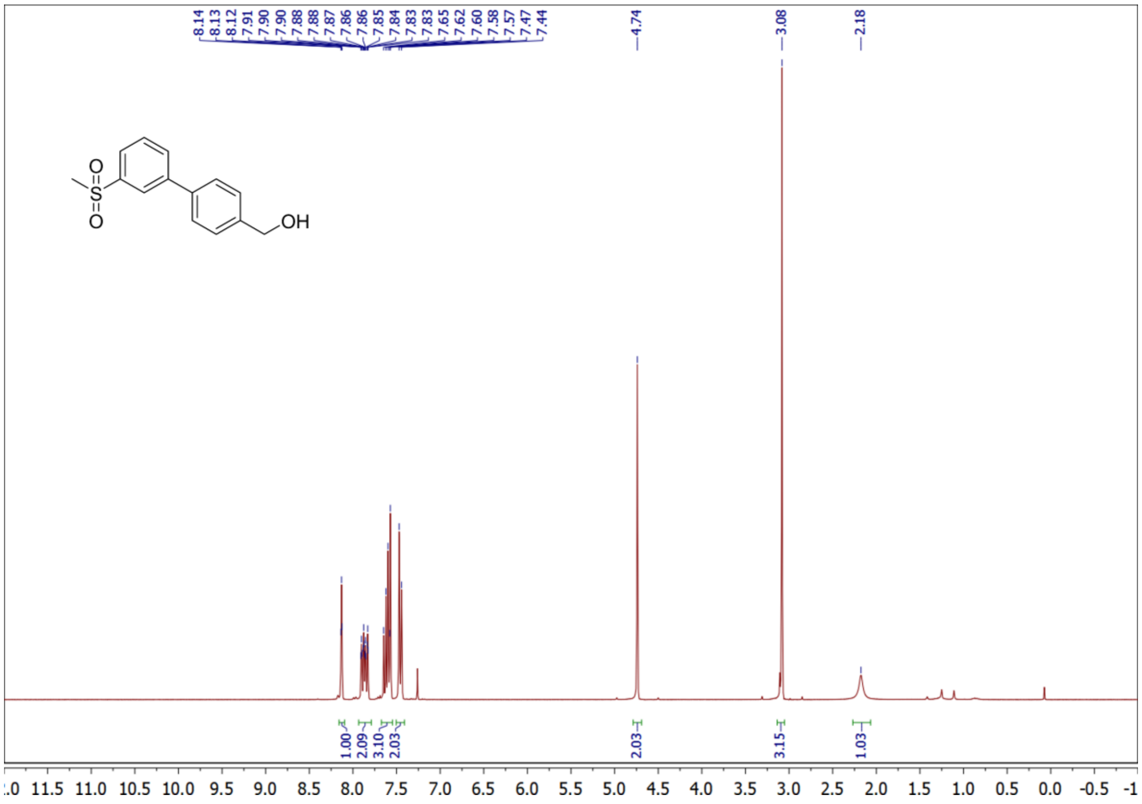

^{13}C NMR (75 MHz, $CDCl_3$)

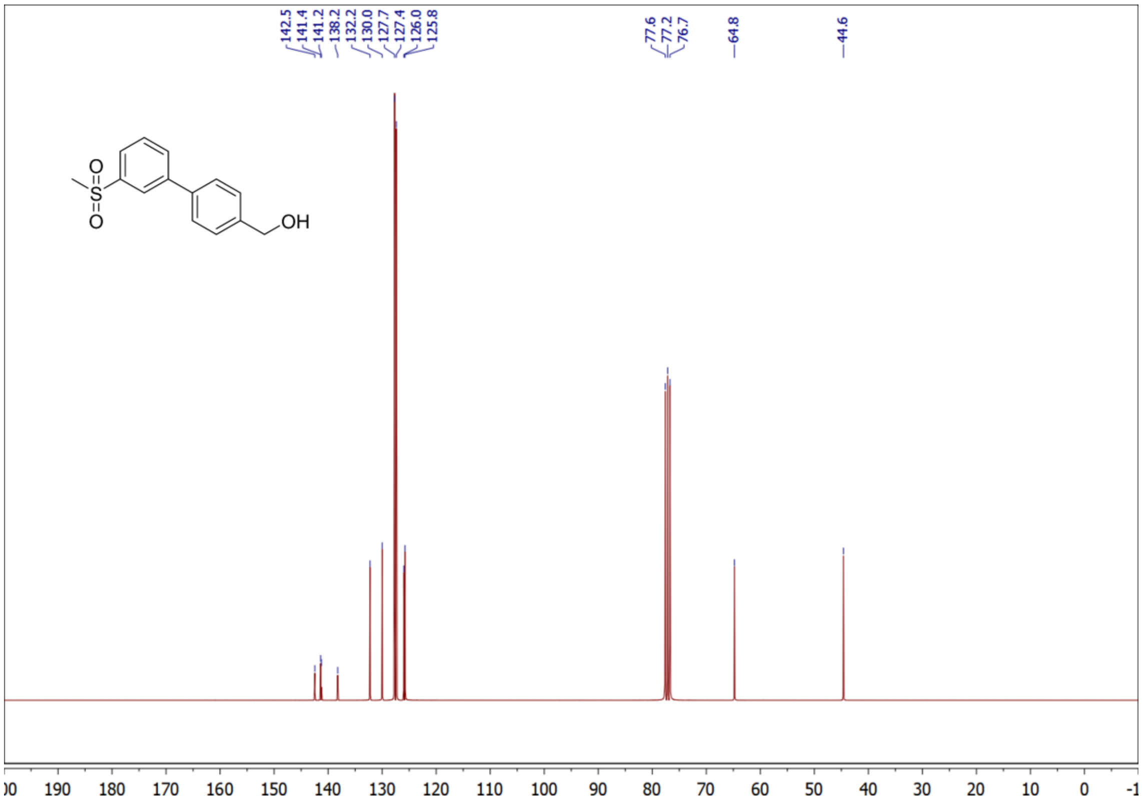

4'-(Bromomethyl)-3-(methylsulfonyl)-1,1'-biphenyl (15)

^{1}H NMR (300 MHz, $CDCl_3$)

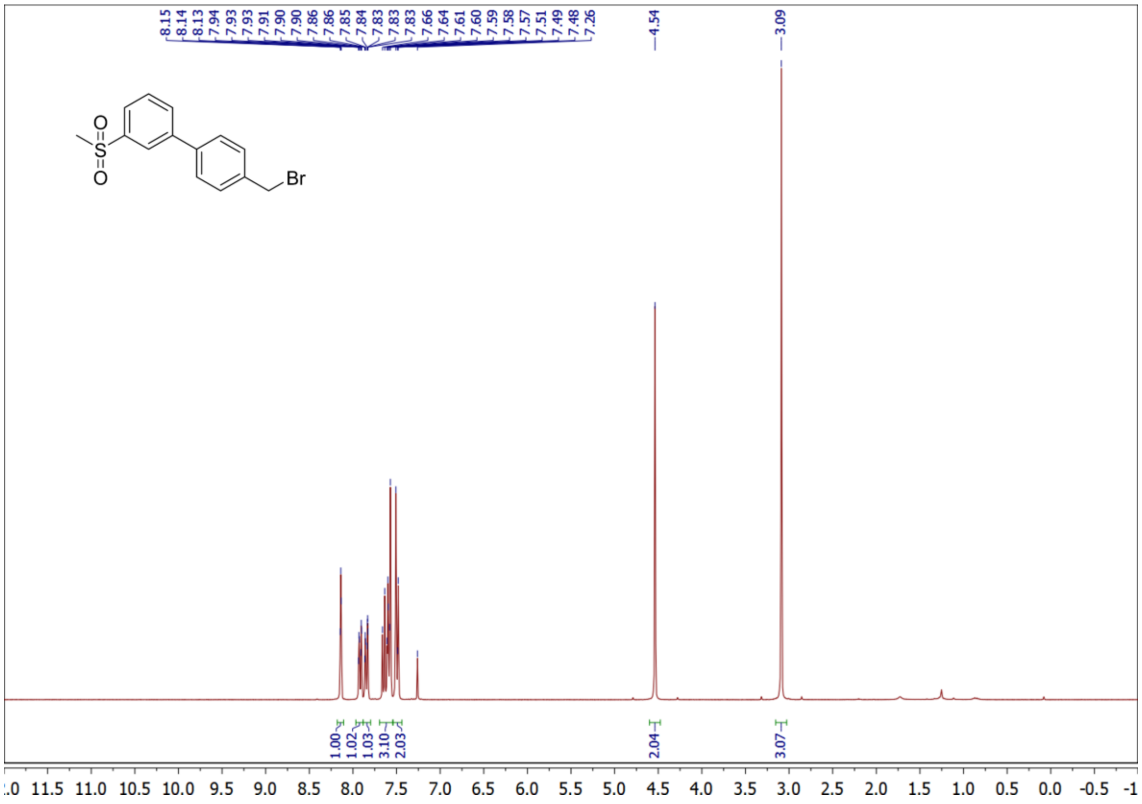

^{13}C NMR (75 MHz, $CDCl_3$)

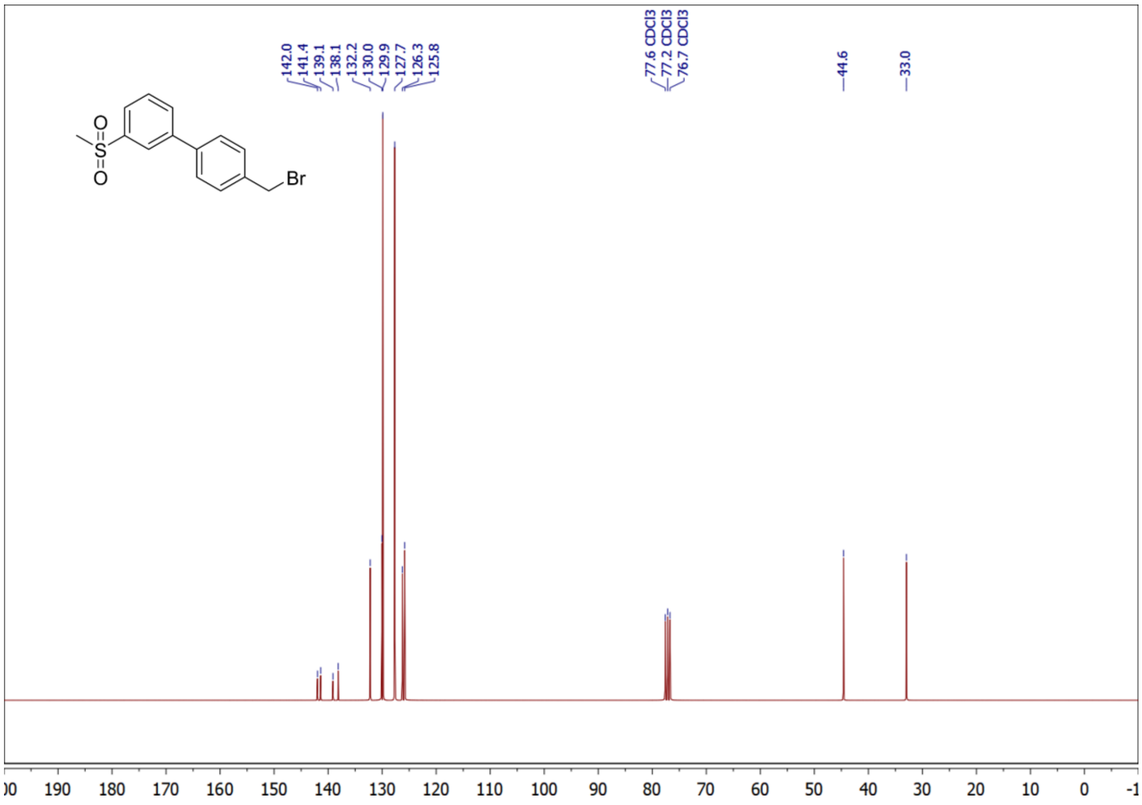

N-([3'-{Methylsulfonyl}-{1,1'-biphenyl}-4-yl]methyl)-2,2-diphenylethan-1-amine (17)

^{1}H NMR (300 MHz, $CDCl_3$)

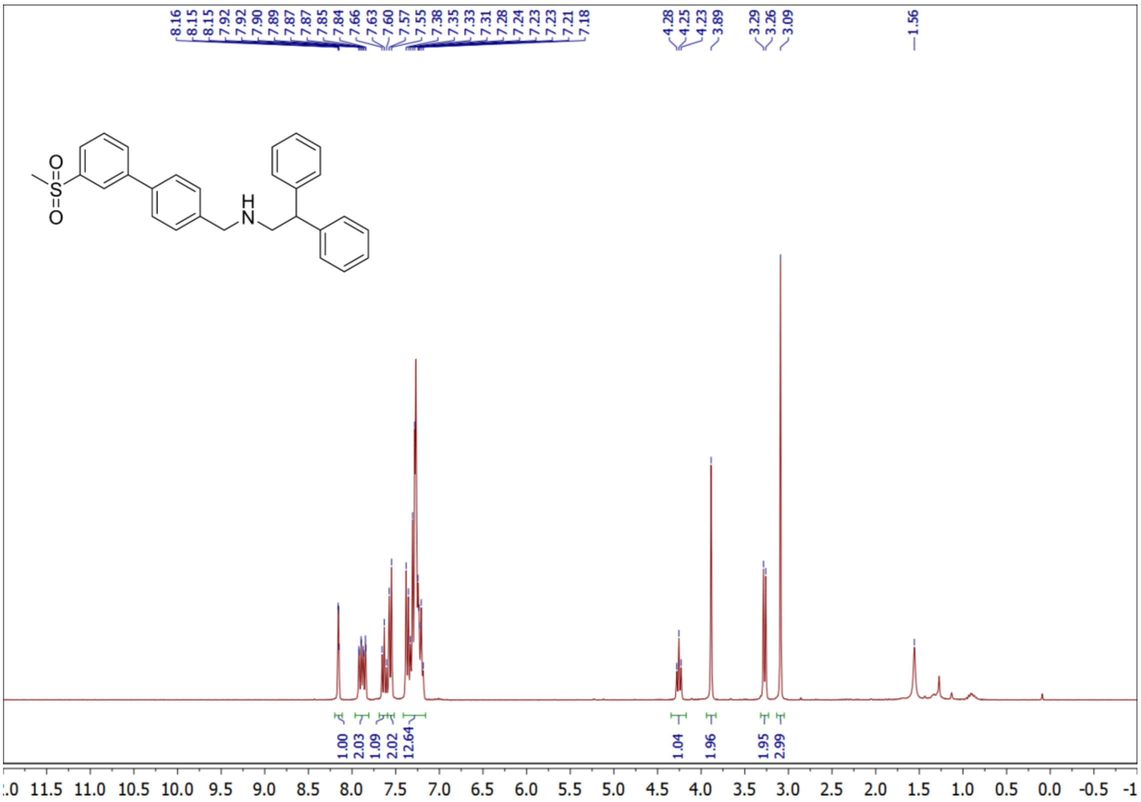

^{13}C NMR (75 MHz, $CDCl_3$)

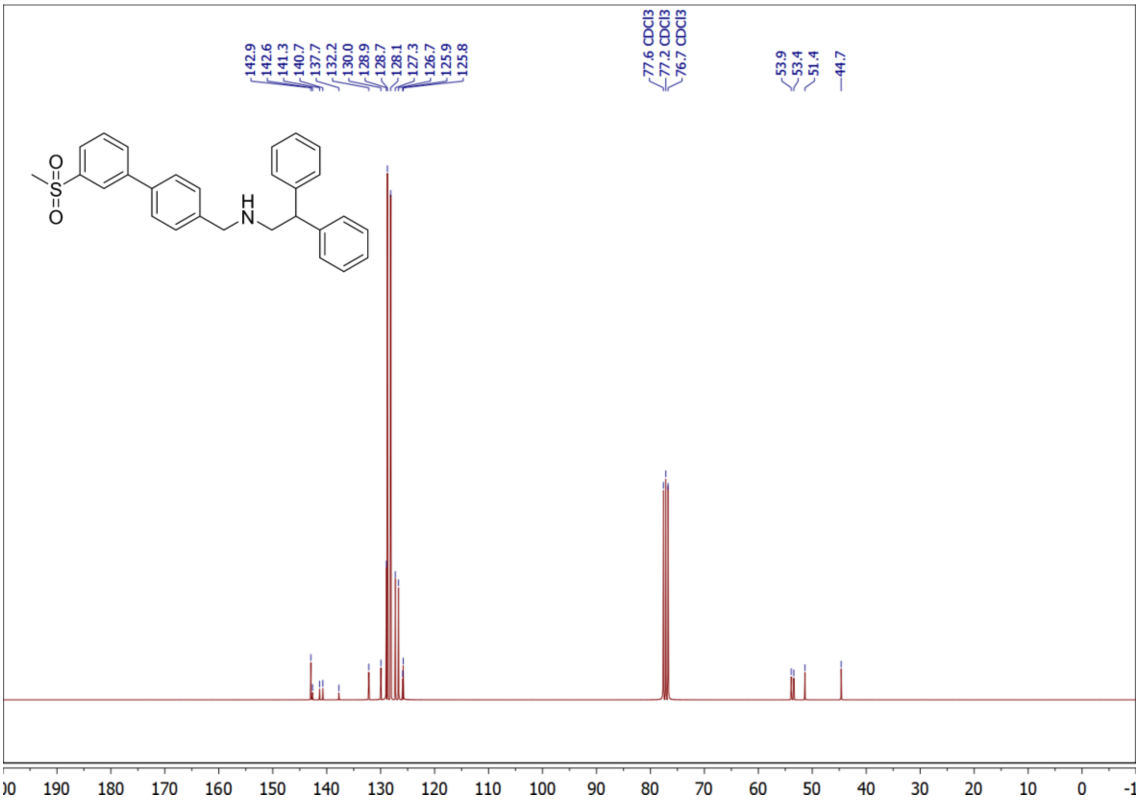

N-(2-Chloro-3-[trifluoromethyl]benzyl)-N-([3'-{methylsulfonyl}-{1,1'-biphenyl}-4-yl]methyl)-2,2-diphenylethan-1-amine (3)

^{1}H NMR (500 MHz, $CDCl_3$)

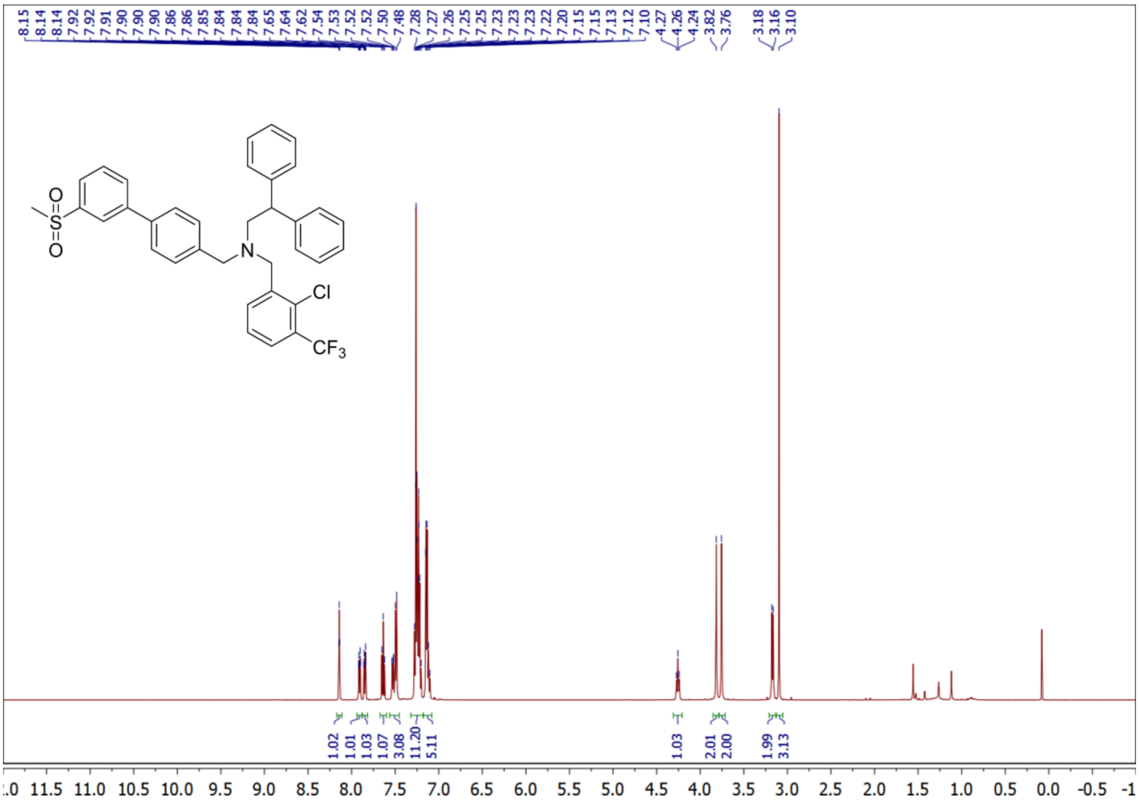

^{13}C NMR (126 MHz, $CDCl_3$)

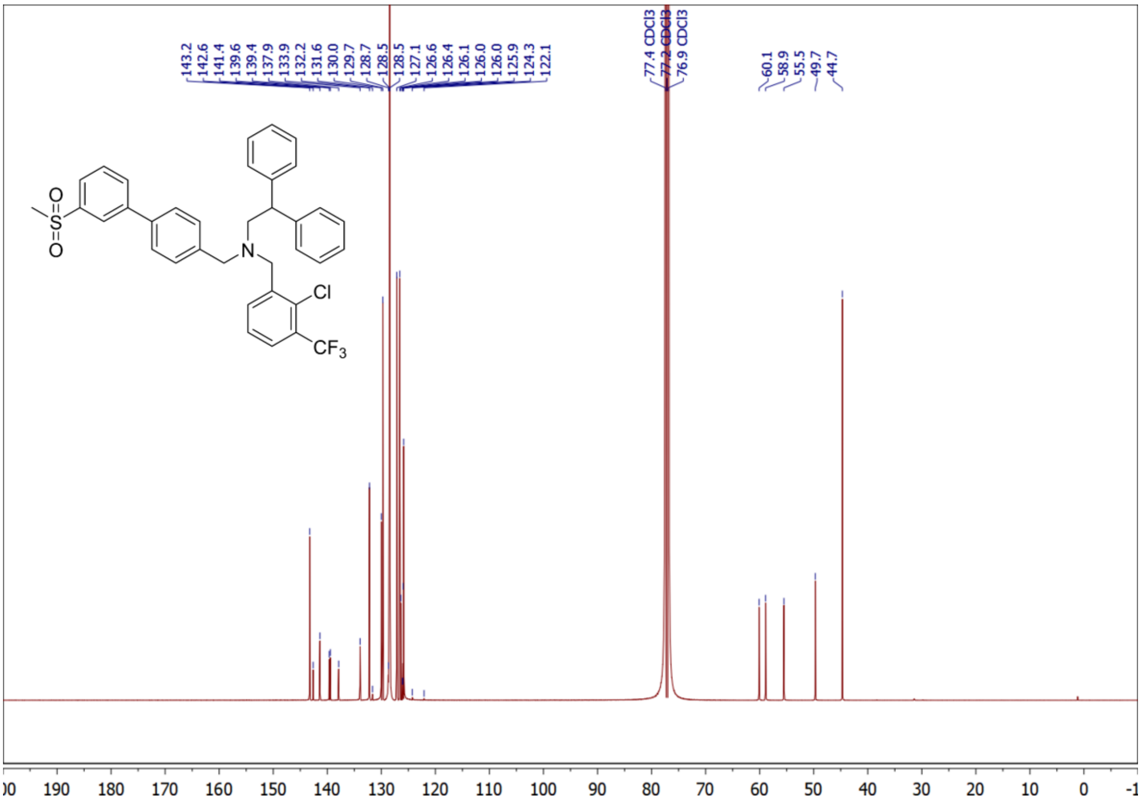

^{19}F-NMR (471 MHz, $CDCl_3$)

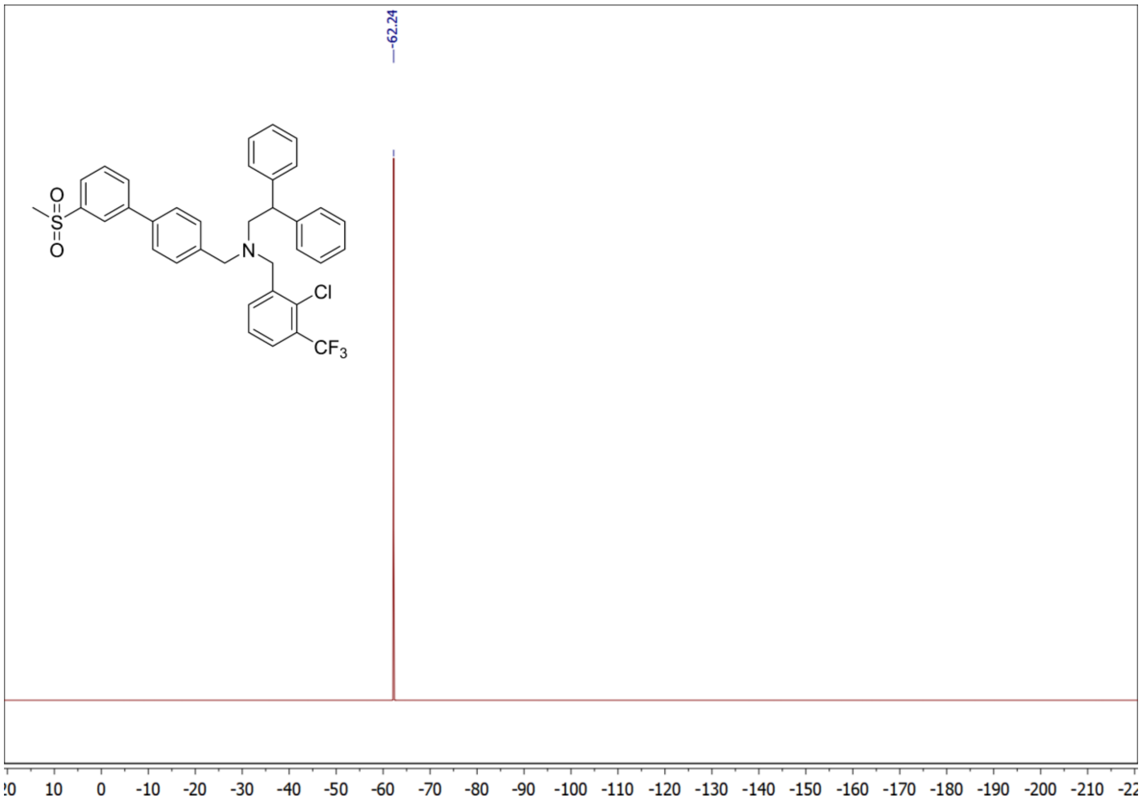

HPLC Traces

Chrom Type: Fixed WL Chromatogram, 254 nm

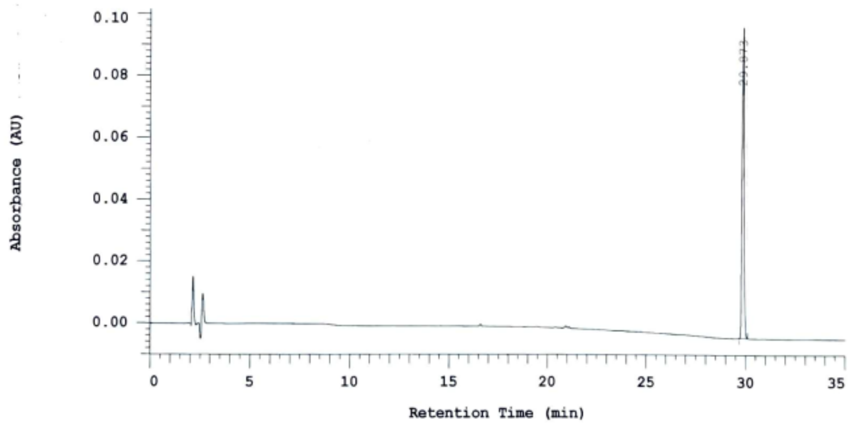

No.	RT	Area	Area %	Height
1	29.873	330200	100.000	50385
		330200	100.000	50385

Peak rejection level: 0

Chrom Type: Fixed WL Chromatogram, 280 nm

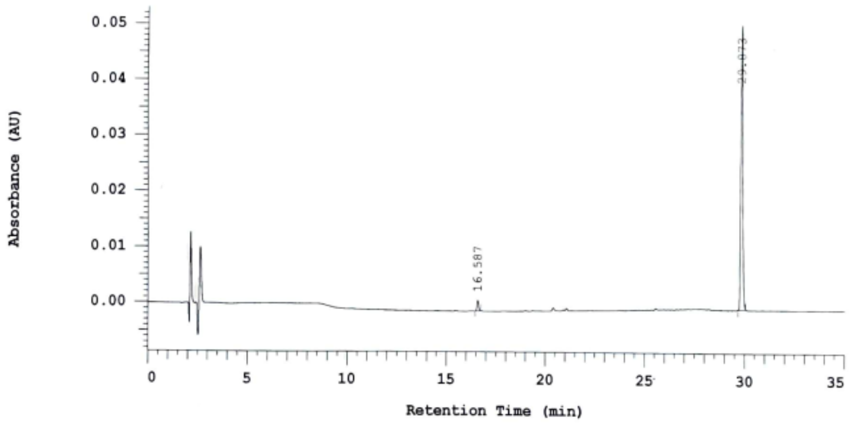

No.	RT	Area	Area %	Height
1	16.587	5143	2.989	929
2	29.873	166950	97.011	25470
		172093	100.000	26399

Peak rejection level: 0

4. Supplementary References

(1) Budzyński, M. A.; Puustinen, M. C.; Joutsen, J.; Sistonen, L. Uncoupling Stress-Inducible Phosphorylation of Heat Shock Factor 1 from Its Activation. *Mol. Cell. Biol.* **2015**, *35* (14), 2530–2540. https://doi.org/10.1128/MCB.00816-14.

(2) Wildman, S. A.; Crippen, G. M. Prediction of Physicochemical Parameters by Atomic Contributions. *J. Chem. Inf. Comput. Sci.* **1999**, *39* (5), 868–873. https://doi.org/10.1021/ci990307l.

(3) Zdrazil, B.; Felix, E.; Hunter, F.; Manners, E. J.; Blackshaw, J.; Corbett, S.; de Veij, M.; Ioannidis, H.; Lopez, D. M.; Mosquera, J. F.; Magarinos, M. P.; Bosc, N.; Arcila, R.; Kizilören, T.; Gaulton, A.; Bento, A. P.; Adasme, M. F.; Monecke, P.; Landrum, G. A.; Leach, A. R. The ChEMBL Database in 2023: A Drug Discovery Platform Spanning Multiple Bioactivity Data Types and Time Periods. *Nucleic Acids Res.* **2024**, *52* (D1), D1180–D1192. https://doi.org/10.1093/NAR/GKAD1004.

(4) Moret, M.; Helmstädter, M.; Grisoni, F.; Schneider, G.; Merk, D. Beam Search for Automated Design and Scoring of Novel ROR Ligands with Machine Intelligence. *Angew. Chem. Int. Ed.* **2021**, *60* (35), 19477–19482. https://doi.org/10.1002/ANIE.202104405.

(5) Kick, E. K.; Busch, B. B.; Martin, R.; Stevens, W. C.; Bollu, V.; Xie, Y.; Boren, B. C.; Nyman, M. C.; Nanao, M. H.; Nguyen, L.; Plonowski, A.; Schulman, I. G.; Yan, G.; Zhang, H.; Hou, X.; Valente, M. N.; Narayanan, R.; Behnia, K.; Rodrigues, A. D.; Brock, B.; Smalley, J.; Cantor, G. H.; Lupisella, J.; Sleph, P.; Grimm, D.; Ostrowski, J.; Wexler, R. R.; Kirchgessner, T.; Mohan, R. Discovery of Highly Potent Liver X Receptor β Agonists. *ACS Med. Chem. Lett.* **2016**, *7* (12), 1207–1212. https://doi.org/10.1021/acsmedchemlett.6b00234.

(6) Fradera, X.; Vu, D.; Nimz, O.; Skene, R.; Hosfield, D.; Wynands, R.; Cooke, A. J.; Haunsø, A.; King, A.; Bennett, D. J.; McGuire, R.; Uitdehaag, J. C. M. X-Ray Structures of the LXRα LBD in Its Homodimeric Form and Implications for Heterodimer Signaling. *J. Mol. Biol.* **2010**, *399* (1), 120–132. https://doi.org/10.1016/J.JMB.2010.04.005.
